# Are many sex/gender differences really power differences?

**DOI:** 10.1093/pnasnexus/pgae025

**Published:** 2024-02-27

**Authors:** Adam D Galinsky, Aurora Turek, Grusha Agarwal, Eric M Anicich, Derek D Rucker, Hannah R Bowles, Nira Liberman, Chloe Levin, Joe C Magee

**Affiliations:** Management Division, Columbia University, New York City, NY 10027, USA; Organizational Behavior Unit, Harvard University, Boston, MA 02163, USA; Organizational Behaviour & Human Resource Management Department, University of Toronto, Toronto, ON M5T 1P5, Canada; Management & Organization Department, University of Southern California, Los Angeles, CA 90089, USA; Marketing Department, Northwestern University, Evanston, IL 60208, USA; Organizational Behavior Unit, Harvard University, Boston, MA 02163, USA; School of Psychological Sciences, Tel Aviv University, Tel Aviv 6997801, Israel; Management Division, Columbia University, New York City, NY 10027, USA; Management & Organizations Department, New York University, New York City, NY 10012, USA

## Abstract

This research addresses the long-standing debate about the determinants of sex/gender differences. Evolutionary theorists trace many sex/gender differences back to natural selection and sex-specific adaptations. Sociocultural and biosocial theorists, in contrast, emphasize how societal roles and social power contribute to sex/gender differences beyond any biological distinctions. By connecting two empirical advances over the past two decades—6-fold increases in sex/gender difference meta-analyses and in experiments conducted on the psychological effects of power—the current research offers a novel empirical examination of whether power differences play an explanatory role in sex/gender differences. Our analyses assessed whether experimental manipulations of power and sex/gender differences produce similar psychological and behavioral effects. We first identified 59 findings from published experiments on power. We then conducted a *P*-curve of the experimental power literature and established that it contained evidential value. We next subsumed these effects of power into 11 broad categories and compared them to 102 similar meta-analytic sex/gender differences. We found that high-power individuals and men generally display higher agency, lower communion, more positive self-evaluations, and similar cognitive processes. Overall, 71% (72/102) of the sex/gender differences were consistent with the effects of experimental power differences, whereas only 8% (8/102) were opposite, representing a 9:1 ratio of consistent-to-inconsistent effects. We also tested for discriminant validity by analyzing whether power corresponds more strongly to sex/gender differences than extraversion: although extraversion correlates with power, it has different relationships with sex/gender differences. These results offer novel evidence that many sex/gender differences may be explained, in part, by power differences.

**Significance Statement**

Compared to men, women earn less money, are less likely to be Chief Executive Officers (CEOs) or world leaders, and control only 1% of executive-held equity. Numerous theories highlight how gender gaps in power contribute to sex/gender differences in psychological tendencies and social behavior. The current empirical analysis compares 102 sex/gender meta-analyses with 59 experimental effects of power to offer novel evidence that many sex/gender differences may be explained, in part, by power differences between men and women. Underappreciating the role of power in observed sex/gender differences adds false credence to gendered expectations and reinforces inequality between men and women. Further establishing that power differences contribute, in part, to sex/gender differences is critical for both understanding and reducing sex/gender-based discrimination.

## Introduction

There is a longstanding debate in the social sciences about the existence and determinants of psychological and behavioral differences between men and women.^a^ Although recent research suggests that men and women are more psychologically similar than they are different (2–5), research also reveals important distinctions between them. For example, research shows that men tend to be more risk-taking (6) and better at mental rotation (7), whereas women tend to be more susceptible to social influence (8) and better at face (9) and emotion recognition (10).

Despite these well-documented sex/gender differences, scientists have not reached a consensus about the underlying causes of those differences. Evolutionary theorists emphasize that many differences between men and women can be traced back to anatomical and physiological roots that emerged through natural selection and sex-specific adaptations (5, 11). According to this perspective, males and females developed distinct physical and psychological attributes that proved adaptive throughout human evolution (5, 11). Paramount among these adaptive forces are the different roles that men and women play in reproduction (12). Competitiveness, risk-taking, and aggressiveness supported male reproductive success because these traits allowed men to secure a greater number of mates (13–15). For women, their essential roles in gestation and lactation led to attributes related to nurturance and communion (11). According to these theories, many psychological and behavioral sex/gender differences are biologically determined and rooted in anatomy and physiology.

In contrast, sociocultural and biosocial theorists across the social sciences highlight how socialization processes, societal roles, and social power have contributed to sex/gender differences that go beyond any anatomical or physiological distinctions (16, 17). Building on psychological and sociological perspectives that gender is socially constructed (18), these theories specify how sex/gender differences can arise from and are reinforced through hierarchical structures, different allocations of resources and tasks (and the values associated with those tasks), and patterns of social interaction between men and women (19). For instance, West and Zimmerman (20) theorize that people enact gender by displaying behaviors in social interactions that have come to be associated with their apparent sex categories through cultural transmission, and they do so in ways that “simultaneously sustain, reproduce, and render legitimate the institutional arrangements that are based on sex category” (p. 146).

Sociocultural and biosocial perspectives acknowledge that the division of males and females into roles is partly based on biological differences between the sexes. Because males are bigger and stronger, historically they have taken on hunter and warrior roles, whereas because women carry fetuses and lactate, they have taken on more nurturing roles (21). However, proponents of these sociocultural perspectives argue that sex-based role divisions have become socially reinforced in ways that go beyond any anatomical or physiological differences between men and women. Furthermore, they note that advances in technology (e.g. birth control pills, formula, breast pumps, surrogacy) have reduced the need for women to be placed in caretaker roles. Similarly, the rise of office professions has reduced the importance of physical strength for many tasks, especially for high-status jobs. More broadly, contemporary organizational life makes biologically based sex/gender differences less relevant.

One of the differences between evolutionary and biosocial theories is how they treat variability between and within males and females. Evolutionary psychology treats males and females as discrete kinds with categorically distinct behavioral repertoires, i.e. males are more likely to share common characteristics with each other than they are with females (22, 23). In contrast, sociocultural and biosocial perspectives view sex/gender differences as dimensional, where men and women vary by degree, not by kind (24, 25). To explore the categorical vs. dimensional views of sex/gender differences, Reis and Carothers (26) examined twenty-two data sets that included 122 self-report measures of attributes with noted sex/gender differences. Supporting the dimensional view, their analyses found that the clustering of attributes within men or women did not reflect a pattern representing separable psychological makeups. The authors concluded that “there is little reason to focus on explanations for categorical distinctions, much less to reify them in essentialist terms” (26). Recent neuroimaging work lends further support to this perspective. Joel and colleagues (27) analyzed more than 1,400 MRIs from four datasets and found that although many features can be classified as predominantly male or predominantly female, brains for both men and women tend to contain features of both types, reflecting gender mosaics (c.f. Del Giudice (28)). This dimensional evidence suggests there may be important variables beyond biological sex that can shift where men and women stand along a variety of characteristics.

Sociocultural and biosocial theories propose that one variable that plays a role in producing sex/gender differences is the psychological experience of power, defined as the actual or perceived control over valuable resources (29–31). Ridgeway and Smith-Lovin (29) note that power differences are often confounded with sex/gender, and Ridgeway and Diekema (32) argue further that “(sex/gender) differences are not produced by any gender-based differences in personality or ability. They are caused by men’s greater power and status in society as a whole” (p. 159). Across domains in society, men frequently have greater power than women within organizations, politics, the economy, and even marital relationships (24). For example, women earn 83.7% of what men earn in the United States and 87% in Europe (33, 34), account for just over 13% of world leaders (35), occupy only 10.4% of Fortune 500 Chief Executive Officers (CEOs) roles (36), hold only 20% of all company board seats globally (37), and control only 1% of executive-held S&P 500 stock (38). Similarly, Eagly and Wood (21) suggest that previously found sex/gender differences in mate preferences across 37 countries could be explained by the fact that women control fewer resources than men in all 37 countries. As these examples make clear, men tend to have more power than women in society. These findings also raise the possibility that power may contribute to observable sex/gender differences (39, 40).

Power differences between men and women are likely to be psychologically consequential because research reveals that having vs. lacking power fundamentally affects individuals’ psychological orientation and behavioral propensities (41, 42). Individuals and groups who possess greater power are structurally advantaged: they have greater access to resources and opportunities, are evaluated more positively, and are deferred to more frequently (43). Furthermore, powerful individuals and groups are often motivated, psychologically and materially, to maintain their advantages and to keep less powerful members in their disadvantaged places (44). As a result of these two processes—one structural and one motivational—having high- vs. low-power produces cognitive, affective, and behavioral effects. The psychological effects of power are so deeply ingrained that randomly assigning individuals to a high- vs. low-power role alters their psychological and behavioral tendencies in that context.

In the current research, we systematically compare the effects of having vs. lacking the power to research sex/gender differences on similar psychological outcomes. For example, research has established that people with greater power exhibit more agency during group tasks while also being less interpersonally sensitive toward others (45–47). Independently, sex/gender difference research has found that men tend to display more agency than women and women tend to be more interpersonally sensitive than men. We propose that these documented differences between men and women might be driven, at least in part, by the fact that men generally have more power in society than women (30, 31, 48).

To explore the link between power differences and sex/gender differences, we connect two empirical advances that have occurred over the past two decades. First, the study of sex/gender differences between males and females has moved beyond single studies to analyses that identify the size of sex/gender differences across many studies, i.e. meta-analyses (16). Published meta-analyses of sex/gender differences increased more than 6-fold from 2001–2005 to 2016–2020 (*N* = 132 in 2001–2005 and *N* = 883 in 2016–2020 using the search terms in Google Scholar “meta-analysis” OR “meta-analytic” AND “sex” OR “gender” in the title). This proliferation of sex/gender difference meta-analyses has even led scholars to conduct meta-syntheses of these existing meta-analyses (4, 5). However, as Eagly and Wood (21) cautioned, the “use of meta-analysis helped to clarify sex difference findings [but] not the causal explanations for these effects.”

**Fig. 1.**
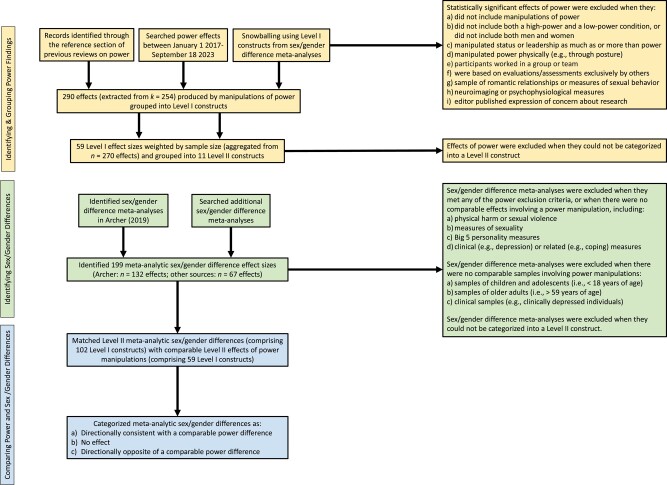
Process for selecting and grouping power-related effects and sex-difference meta-analyses. The Level III categories are not depicted as they were created for ease of presentation.

This brings us to the second empirical advance: the explosion of experimental research on the psychological effects of power. Motivated by theoretical and methodological innovations, the number of papers with experiments on power increased 7-fold from 2001–2005 to 2016–2020 (*N* = 59 in 2001–2005 and *N* = 412 in 2016–2020, using the search terms in Google Scholar “manipulation of power” OR “power manipulation” OR “power was manipulated” AND “high power” AND NOT “power distance” anywhere in the article) (41, 42, 49). Researchers using experimental manipulations of power have documented dozens of distinct effects of power on cognition, affect, and behavior (41).

## Overview and analytic set-up

To examine the similarity between power differences and sex/gender differences, we first identified experiments that demonstrated a causal role of power. We then compared these findings to the results of meta-analyses of sex/gender differences on conceptually similar outcomes. We predicted that when participants in high-power experimental conditions score higher than participants in low-power experimental conditions on a particular outcome, men will score higher than women on the same or similar outcome. Conversely, when participants in low-power conditions score higher on an outcome than participants in high-power conditions, we predict females will score higher than males on the same or a similar outcome.

Given there are more documented sex/gender differences than the effects of experimental power differences, our project scope was determined by experimental research on the effects of power. We began by identifying outcomes significantly affected by experimental manipulations of power, consistent with a *P*-curve approach (50, 51). We then examined whether conceptually related constructs had been meta-analytically tested for sex/gender differences (see Methods section and Fig. 1 for the detailed steps of this process).

To identify outcomes based on power differences, we took the following steps (outlined in our flow chart in Fig. 1 and following Preferred Reporting Items for Systematic Reviews and Meta-Analyses (PRISMA) guidelines described in Table S11). First, we identified power effects from the reference lists of two reviews of the power literature, Galinsky et al. (41) and Guinote (52). We selected these reviews because they comprehensively surveyed 155 unique empirical journal articles them. We then supplemented these papers with searches for more recent papers that experimentally manipulated power. For our analyses, we only included effects from papers involving at least one experiment (i.e. manipulation of power), included both a high-power and low-power condition, and produced a significant effect. The decision to require both high- and low-power conditions was made to match sex/gender difference analyses; that is, our core proposition connects men with high-power conditions and women with low-power conditions. We only included experiments with significant results, consistent with a *P*-curve approach, because we were interested in comparing sex/gender differences to already established causal effects of experimental manipulations of power.

After applying additional exclusion rules (detailed in the Methods section and outlined in Fig. 1), we identified 290 effects of power. Next, we used an iterative process to combine effects that involved measures that were conceptually related, if not identical. This aggregation process produced 69 effects of power. For each effect, we computed an effect size, weighted by the sample size of each study (for more details see the Methods section).

We then identified a recent meta-synthesis of the sex/gender differences literature (5) that provided a thorough review of meta-analyzed sex/gender differences (we relabeled some constructs; see Table S4). For all sex/gender differences, we also searched for more recent meta-analyses (see Table S1). This process yielded 199 sex/gender difference meta-analytic effects to be considered for inclusion. After applying our exclusion rules, we included 126 sex/gender difference effects for further consideration (see the flow chart Fig. 1 and the Methods section for exclusion rules).

One challenge with comparing the experimental effects of power with meta-analytic sex/gender differences is that these literatures took shape independently, with researchers studying conceptually similar but not always identical constructs, while often using different measures to study similar phenomena. To compare constructs across the power and sex/gender differences literatures, we took the following steps (detailed in the Methods section). First, we compiled a list of constructs from the experimental power effects and the sex/gender difference meta-analytic effects. We refer to these as Level I constructs. Second, we used an iterative process to develop broader and more inclusive constructs (referred to as Level II constructs) into which Level I constructs could be categorized. These Level II constructs were crucial for our comparison of power effects to sex/gender differences and maximized a specificity-generality tradeoff: the constructs were not so specific that there would be too few findings to compare and not so general that comparisons would be meaningless. We then had three field experts independently categorize Level I constructs into Level II constructs, eliminating any Level I constructs for which there was insufficient agreement or rationale for which Level II construct it belonged.

We were able to categorize 59 of the 69 Level I power constructs and 102 of the 126 Level I sex/gender constructs. For the other 34 constructs, there was insufficient agreement between the independent judges, and thus an applicable Level II construct could not be identified in the literature; as a result, these constructs were excluded from further analysis. Tables 1–4 depict the Level I power effects and sex/gender difference effects categorized within the 11 Level II constructs. Finally, for ease of presentation, we organized Level II constructs into four broader categories, which we refer to as Level III categories (agency, communion, self-evaluation, and cognitive processes).

### Categorization of sex/gender effects as consistent with or opposite of power effects

Both Hyde (2) and Funder and Ozer (53) argue that any Cohen's |*d*| > 0.10 is psychologically meaningful. Funder and Ozer (53) explain why this standard is particularly appropriate for independent variables that are relatively fixed and experienced repeatedly over time by the same individuals. Sex/gender, as a demographic and identity-based construct, is a quintessential example of a relatively fixed and repeatedly experienced variable. As such, even a very small sex/gender difference can have a substantial impact on life outcomes. For example, one study found that even when only 1% of the variance in performance ratings could be attributed to sex/gender differences, it led to 65% of the highest level positions being filled by men and only 35% being filled by women (54). In a review of sex/gender difference meta-analyses, Archer (5) also classified |*d*| > 0.10 as a nonzero effect.

For our analyses, we considered a documented meta-analytic sex/gender difference to be consistent with or opposite of a conceptually related experimental effect of power when both effects were |*d*| > 0.10 (and similarly Hedges’ |*g*| > 0.10). More specifically, we considered a meta-analytic sex/gender difference to be consistent with a conceptually related power effect when both effects were either *d* > 0.10 or *d* < −0.10. We considered a sex/gender difference to be directionally opposite of a conceptually related power differences when one *d* > 0.10 and the other was *d* < −0.10, i.e. the *d*s had opposite signs and the absolute value of each was greater than 0.10. We considered any meta-analytic sex/gender difference to be a null effect if |*d*| ≤ 0.10.

## Results

We first examined whether the effects from experimental manipulations of power contain an evidential value. We next compared the correspondence between meta-analytic sex/gender differences and the effects of experimental manipulations of power. We first present the overall results and then separately for our Level III categories (agency, communion, self-evaluation, and cognitive processes). For ease of reading, we omit citations in the text and provide them as references in Tables 1–4.

### *P*-curve of experimental power effects

**Fig. 2.**
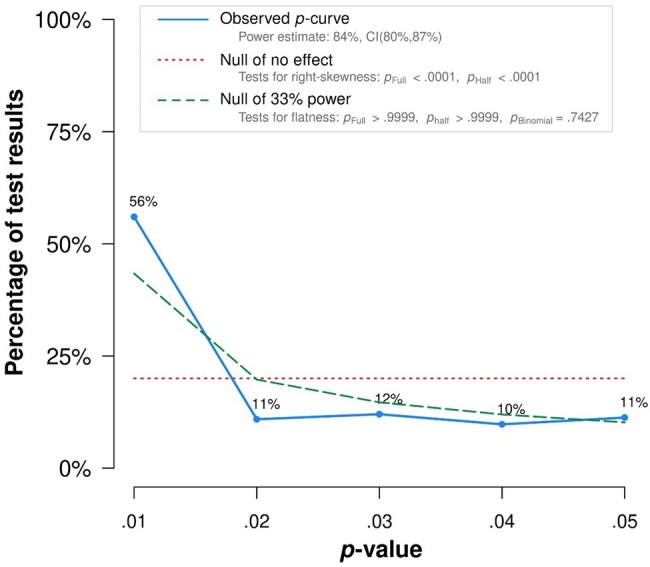
*P*-curve for effects of power. This figure was generated by *P*-curve app 4.06, available at *P*-curve.com by inputting the 270 significants effects from the power litereature. The *P*-curve analyses demonstrate that the power literature contains evidential value as both the half and full *P*-curves pass the right-skew tests, *P*s < 0.0001. In addition, the results do not support an absence of evidential value, i.e. a *P*-curve that is flatter than what we would expect for studies with an average power of 33% (*P*_full_ = 0.9999; *P*_half_ > 0.9999). Finally, the average power estimate for these experimental studies was 84%. Altogether, these results suggest that the experimental literature on power has evidential value.

To test for evidential value in the power experiments considered for our analysis, we conducted a *P*-curve analysis of the experimental power literature (*k* = 270). As Fig. 2 details, both the half and full *P*-curves pass the right-skew tests, *P* < 0.0001. Furthermore, the results do not support an absence of evidential value, i.e. a *P*-curve that is flatter than what we would expect for studies with an average power of 33% (*P*_full_ = 0.9999; *P*_half_ > 0.9999). Finally, the average power estimate for these studies was very high at 84%. Altogether, these results suggest that the literature on experimentally induced power differences has evidential value.

We also conducted separate *P*-curves for each of our Level III categories. For agency, communion, and self-evaluation, the half and full *P*-curves pass the right-skew tests, *P* < 0.0001 and the *P*-curve is not flatter than what we would expect for studies with an average power of 33% (*P*_full_ = 0.9999; *P*_half_ > 0.9999). For cognitive processes, the half (*P* < 0.0001) and full (*P* = 0.008) *P*-curves pass the right-skew tests and the *P*-curve is not flatter than what we would expect for studies with an average power of 33% (*P*_full_ = 0.5829; *P*_half_ > 0.9999). These *P*-curve results demonstrate that the power literature contains an evidential value for agency, communion, self-evaluation, and cognitive processes.

### Experimental Power differences and sex/gender differences overall

**Fig. 3.**
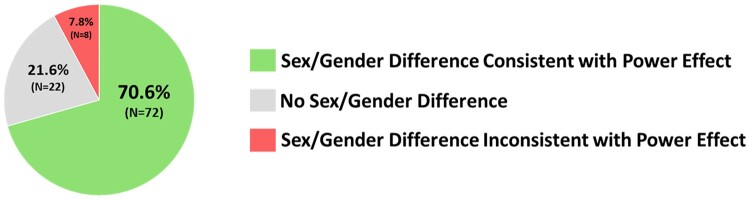
Overall comparison of the experimental power literature and sex/gender difference meta-analyses. Overall, 70.59% (72 of 102 of Level I constructs) of sex/gender differences were consistent with the effects of experimentally induced power differences, whereas only 7.84% were inconsistent.

Our analysis found that 70.59% (72 of 102 Level I constructs) of sex/gender differences were consistent with the effects of experimentally induced power differences, while only 7.84% (8 of 102 Level I constructs) were inconsistent (see Fig. 3). High-power individuals and men tend to display higher agency, more positive self-evaluations, lower communion, and similar cognitive processes compared to low-power individuals and women.

### Experimental Power differences and sex/gender differences in agency

Agency represents the striving to be independent, to control one's environment, and to assert the self; agency captures the tendencies to be active, efficient, and assertive (55). Within the agency category, we included dominance, goal approach/disinhibition, and risk-seeking as Level II constructs. As summarized in Table 1, 66% (27 of 41 Level I constructs) of the agency-related sex/gender difference meta-analyses were directionally consistent with the effects of experimental manipulations of power, whereas only 5% (2 of 41 Level I constructs) of these sex/gender difference meta-analyses were opposite of power differences. Overall, high-power individuals and men display more dominance, more goal approach and disinhibition, and greater risk-seeking than low-power individuals and women.

**Table 1.** Comparison of agency effect sizes from the power literature and sex/gender difference meta-analyses.

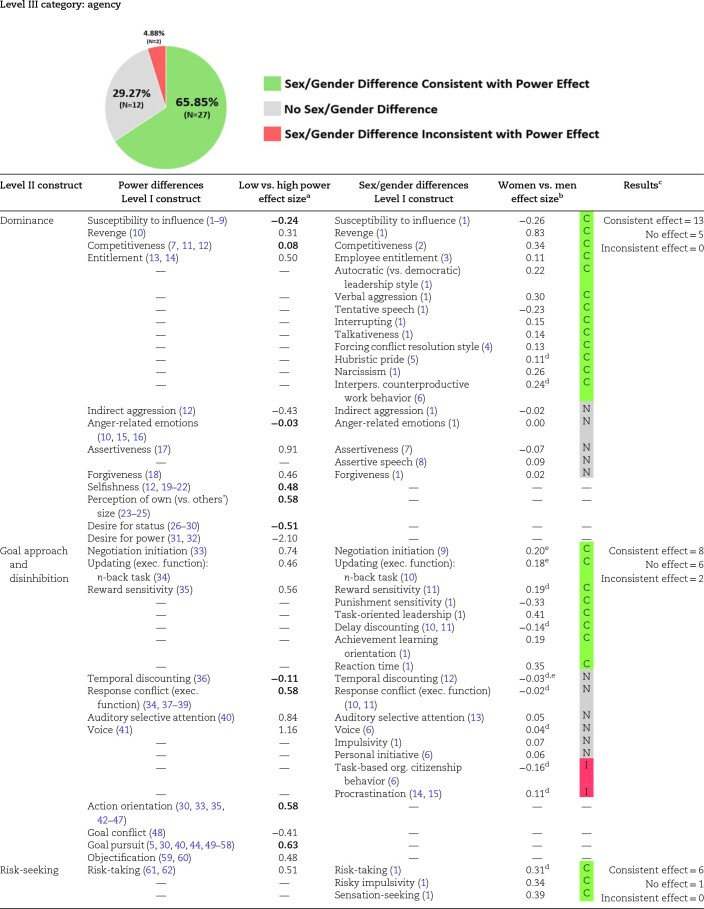 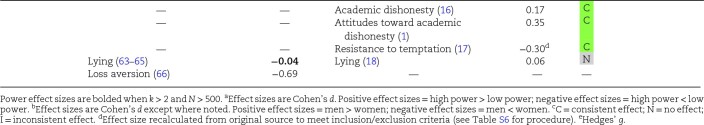

Looking at the components of agency, high-power individuals tend to be more dominant, selfish, vengeful, assertive, and entitled and are more likely to see themselves as physically larger than others. In contrast, low-power individuals tend to display greater susceptibility to influence, greater desire for status and power (presumably to address their lack of power), more indirect (e.g. covert) aggression, and less forgiveness. Power was unrelated to anger and competitiveness, i.e. *d* < 0.10. In terms of goal approach and disinhibition, power affected all eleven of these outcomes. With respect to risk-seeking, high-power individuals tend to take more risks and be less loss-averse; however, power is unrelated to lying.

Meta-analytic sex/gender differences on agency-related constructs show that men are overwhelmingly more dominant and risk-seeking, and somewhat more oriented toward goal approach and disinhibition. Although there was no sex/gender difference (i.e. |*d*| < 0.10) for more than one-third of goal approach and disinhibition outcomes, the only effects that were opposite of power's positive relationship were that men procrastinated more and showed less task-based organizational citizenship behavior than women.

### Experimental power differences and sex/gender differences in communion

Communion represents how people think about themselves in relation to others and behave toward others in a way that emphasizes social connection and cooperation; communion captures the tendencies to be helpful, understanding, empathic, and sociable (31, 47). Within the communion category, we included interpersonal sensitivity, sociability, and prejudice and dehumanization as Level II constructs; we predicted that prejudice/dehumanization effects would have the opposite sign to the effects in interpersonal sensitivity and sociability. As summarized in Table 2, 80% (20 of 25 Level I constructs) of the communion-related sex/gender difference meta-analyses were directionally consistent with experimental effects of power, whereas only 4% (1 of 25 Level I constructs) of these sex/gender difference meta-analyses were opposite of power differences. Overall, high-power individuals and men tend to display less interpersonal sensitivity, less sociability, and more prejudice/dehumanization than low-power individuals and women.

**Table 2.** Comparison of communion effect sizes from the power literature and sex/gender difference meta-analyses.

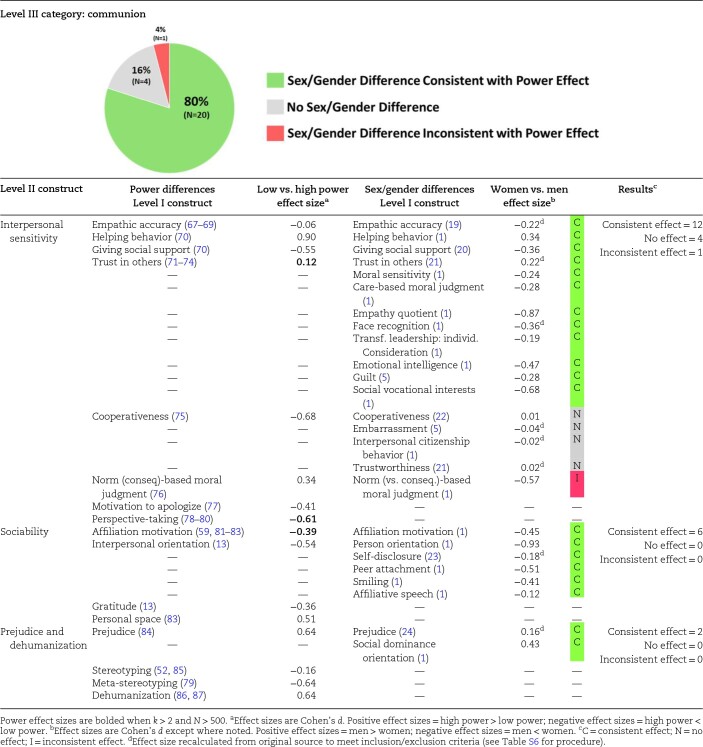

Examining the components of communion, power has a negative effect on interpersonal sensitivity; low-power individuals tend to be more cooperative, more likely to take others’ perspectives, more motivated to apologize, and to offer more social support. Unexpectedly, high-power individuals tend to display more trust in others, moral judgment based on social norms, and helping behavior. Power is unrelated to empathic accuracy, i.e. |*d*| < 0.10. Power also shows a negative effect on sociability; low-power individuals tend to have stronger affiliation motivation and interpersonal orientation, show a preference for less personal space, and express more gratitude. Finally, high-power individuals display more prejudice and dehumanization but less stereotyping (both stereotyping others and meta-stereotyping).

Meta-analyses of sex/gender effects show that women tend to be more communal across all three components: women tend to be more interpersonally sensitive, more sociable, and less prejudiced. When compared with the power effects, the only directionally opposite effect is that women generally rely more on social norms on their moral judgments.

It should also be noted that although the sex/gender effect for helping behavior is directionally consistent with the effect of power, both are surprising because men and high-power individuals tend to be more likely to help others. Probing the literature on helping behavior reveals that a great deal of helping behavior requires agency and risk-taking (e.g. overcoming bystander nonresponsiveness and taking assistive action, sometimes in the face of danger) (56, 57) and thus this construct also reflects a high degree of goal approach/disinhibition and risk-seeking. This point is reinforced by the fact that the two studies responsible for the effect size of power on helping behavior measured intention to confront a perpetrator of workplace incivility (58).

### Experimental power differences and sex/gender differences in self-evaluation

Self-evaluation represents an assessment of one's ability and an evaluation of one's experiences. Within the self-evaluation category, we included positive views of the self and well-being as Level II constructs. As summarized in Table 3, 65% (17 of 26 Level I constructs) of the self-evaluation sex/gender difference meta-analyses were directionally consistent with experimental effects of power, whereas only 12% (3 of 26 Level I constructs) of these sex/gender difference meta-analyses were opposite of power differences. Overall, high-power individuals and men tend to have more positive views of the self and higher well-being than low-power individuals and women.

**Table 3.** Comparison of self-evaluation effect sizes from the power literature and sex/gender difference meta-analyses.

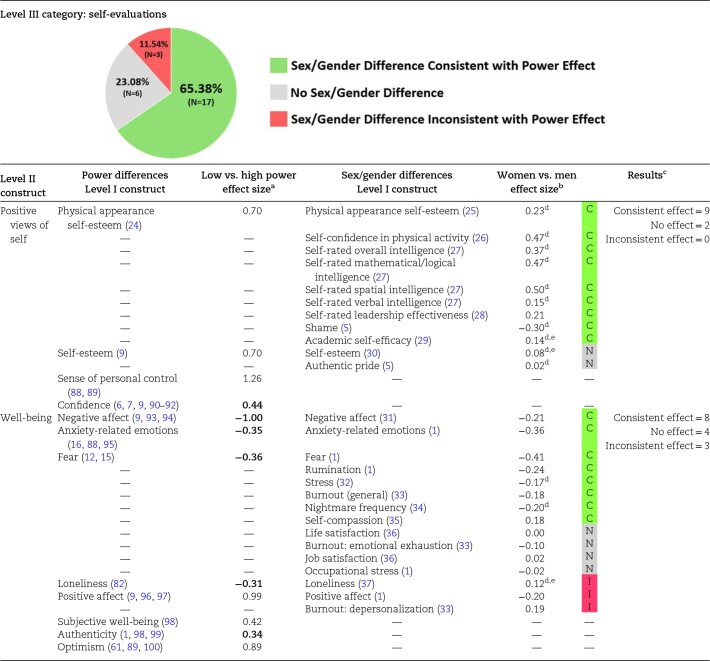

In examining our components of self-evaluation, we found no exceptions to the positive relationships between power and positive views of the self and well-being.

Meta-analyses on sex/gender differences in positive views of the self-reveal that men tend to have higher academic self-efficacy, physical appearance self-esteem, self-rated intelligence across several domains, self-rated leadership effectiveness, and lower shame; there is no sex/gender difference in global self-esteem or authentic pride, i.e. *d* < 0.10. With respect to well-being, men tend to fare better than women overall; however, women tend to experience more positive affect, less loneliness, and less depersonalized burnout. There are no sex/gender differences in life satisfaction, job satisfaction, occupational stress, or emotional burnout, i.e. |*d*| < 0.10.

### Experimental power differences and sex/gender differences on cognitive processes

Cognitive processes include attention, perception, memory, reasoning, problem-solving, and decision-making. Within the cognitive processes category, we included spatial ability/performance, creative performance, and abstract cognition as Level II constructs. As summarized in Table 4, 80% (8 of 10 Level I constructs) of the cognitive processes sex/gender difference meta-analyses were directionally consistent with experimental effects of power, whereas only 20% (2 of 10 Level I constructs) of these sex/gender difference meta-analyses were opposite of power differences. Overall, high-power individuals and men tend to have higher performance on spatial tasks and creative tasks, and their cognition tends to be more abstract than low-power individuals and women.

**Table 4.** Comparison of cognitive processes effect sizes from the power literature and sex/gender difference meta-analyses.

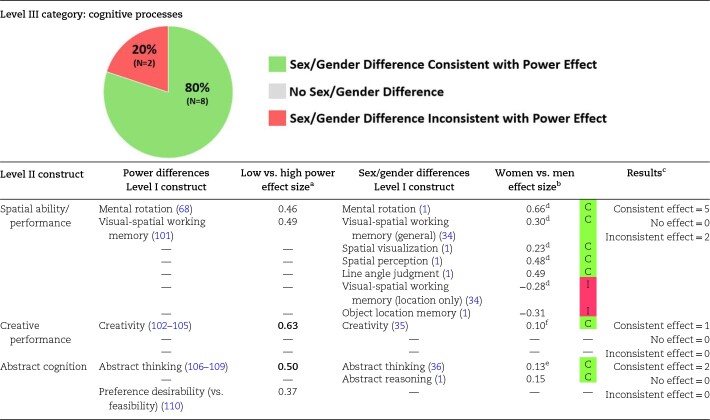

Research on power is relatively sparse in this area, but experiments have shown that high-power individuals tend to exhibit better mental rotation, greater creativity, and more abstract thinking than low-power individuals.

For sex/gender differences related to spatial ability, men tend to score higher than women on all tasks except those that involve exclusively location memory, where women tend to score higher than men. Men also tend to score higher than women on measures of creative performance and abstract cognition.

## Discriminant validity: the case of extraversion

We have proposed that the parallel findings between experimental manipulations of power and meta-analyses of sex/gender differences offer suggestive evidence that power may play an explanatory role in producing sex/gender differences. However, the parallels between power differences and sex/gender differences only support but do not fully confirm the theoretically well-justified proposition that power has a particularly strong association with sex/gender differences. Indeed, it is possible that other variables show a similar correspondence with sex/gender differences, even if there is not a strong theoretical basis for their correspondence. According to this line of reasoning, the parallel findings we have found between power differences and sex differences could be merely artifactual.

To address this potential issue that the power-sex/gender association is artifactual, we explored discriminant validity by identifying a comparison variable with three specific properties. The comparison variable needed to (i) have documented associations with a wide range of variables, similar to the over 100 constructs in the power-sex/gender analysis; (ii) exhibit a positive empirical association with power but (iii) not exhibit the same relationship with sex/gender. If such a variable turns out to correspond with sex/gender differences in a similar direction and strength as power, it would constitute a threat to our claim that power has a particularly strong connection to sex/gender differences. However, if the correspondence of sex/gender effects with the effects of this variable is substantively distinct from the correspondence between power and sex/gender, this would provide additional support to our claims.

Before describing the variable that we used, we note that we considered other potential variables to test for discriminant validity but could not identify any others that met our three criteria. For example, as one of the big five personality variables (59), conscientiousness satisfies our first criterion because it has been studied extensively, yielding a substantial empirical basis for its associations with a wide range of outcomes. However, conscientiousness fails to satisfy our second criterion as it does not have a consistent relationship with power (60, 61). Similarly, money satisfies our second criterion because it is a conceptual and material correlate of power, but it fails to satisfy our first criterion as research on the effects of priming money covers a more narrow set of dependent measures compared to sex/gender differences (62, 63).

Extraversion is one variable that satisfies these three criteria well. First, extraversion has been studied extensively and is associated with a wide range of variables. Second, extraversion is correlated with individuals’ power and influence (64, 65). Thus, it is plausible that extraversion might behave similarly to power in a comparative analysis with sex/gender differences. Third, in contrast to power, women are modestly more extraverted than men (66, 67). Furthermore, any overall sex/gender difference in extraversion (0.10 ≤ *r*s ≤ 0.30) is weaker than the association between extraversion and power/influence (0.29 ≤ *r*s ≤ 0.46) (60, 64–67). Moreover, the subfacets of extraversion suggest that extraversion's overall associations with our outcome variables would not all go in the same direction as power. In fact, across the extraversion subfacets, sex/gender differences go in opposing directions, with women tending to be higher on warmth but men tending to be higher on assertiveness (68). In contrast to power, which is *negatively* related to warmth, extraversion is *positively* related to warmth. Therefore, we expected that extraversion would exhibit a different pattern with sex/gender differences than does power with our outcome variables.

Similar to our search for sex/gender differences, we located a meta-synthesis of extraversion's effects (we relabeled some constructs; see Table S5) (69). We also conducted a search for more recent extraversion meta-analyses involving the Level I constructs from the power experimental effects and sex/gender difference meta-analyses. This search strategy was comparable in its comprehensiveness to our search strategy for identifying sex/gender difference meta-analyses (see Table S1). We located 201 meta-analytic effects of extraversion and applied the same exclusion rules and categorization process that we applied to the sex/gender meta-analyses. Ultimately, we identified 85 extraversion meta-analytic effects to compare to sex/gender differences.

**Fig. 4.**
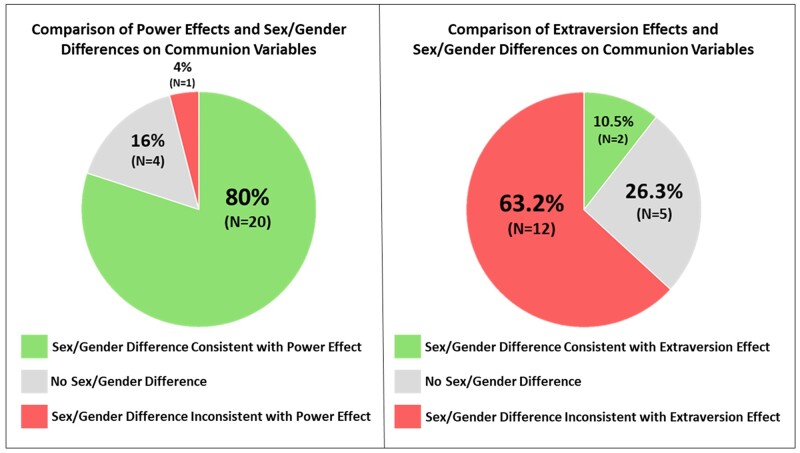
Sex/gender differences comparisons with experimental power effects and extraversion. Comparison of power effects and sex/gender differences on communion-related outcome variables (left panel) and comparison of extraversion effects and sex/gender differences on communion-related outcome variables (right panel). Sex/gender difference meta-analyses were coded as consistent with power effects when both men and high-power conditions scored higher on a construct. Sex/gender difference meta-analyses were coded as consistent with extraversion effects when both men and high extraverts scored higher on a construct.

Similar to the effects of power, extraversion was positively related to agency and self-evaluations (see Table S7). For cognitive processes, the pattern was less clear; extraversion was positively related to creative performance and abstract cognition, but not to spatial ability. In contrast to power, however, extraversion was *positively* related to communion. Recall that, for our communion constructs, sex/gender difference meta-analytic effects were consistent with power effects 80% of the time, with men and high-power experimental conditions scoring lower on communion. In contrast, men scored lower, whereas extraverts scored higher on communion; only 11% of communion effects were in the same direction for men and extraverts (see Fig. 4). These results establish that there is a greater consistency in the overall association between power and sex/gender differences than between extraversion and sex/gender differences.

## Discussion

The current analyses revealed a striking correspondence between the psychological and behavioral effects produced by experimental power differences and meta-analytic sex/gender differences. Our analytic approach capitalized on two empirical advances in the last two decades. One advance was a dramatic increase in experimental work that has manipulated social power to understand its causal effects on a wide range of psychological and behavioral variables. We documented for the first time that this body of work has evidential value by conducting a *P*-curve analysis. The other advance is the explosion of meta-analyses of sex/gender differences. We reasoned that sex/gender differences that are directionally consistent with the effects produced by experimental manipulations of power—with men and high-power individuals showing similar tendencies, and, likewise, women and low-power individuals showing similar tendencies—would offer suggestive evidence that those sex/gender differences may be driven, at least in part, by power differences between men and women.

We found robust evidence that many well-documented sex/gender differences in the domains of agency, communion, self-evaluation, and cognitive processes are directionally consistent with experimentally created power differences. Overall, sex/gender differences are nine times more likely to show a pattern consistent (70.59%, 72 of 102) with experimental power differences than to show the opposite pattern (7.84%, 8 of 102). The sex/gender difference meta-analyses are directionally consistent with experimentally created power differences in each category: agency (66%), communion (80%), self-evaluation (65%), and cognitive processes (80%). High-power individuals and men generally display higher agency, lower communion, more positive self-evaluations, and similar cognitive processes.

Of the few findings that are in the opposite direction of our hypotheses, two are for multifaceted behaviors that involve a mix of agency, communion, and/or well-being. Men scored higher than women on helping behavior; although our coders categorized helping as communal, Eagly and Crowley (56) point out the behavior itself often comprises heroic and assertive actions. Similarly, women experience less loneliness, a measure of well-being that taps into the subjective experience of social connection. Through the lens of women being more communal in relationships, their lower loneliness is not very surprising. A similar explanation exists for the null effect of power on lying, which is driven by a number of experiments studying a particular type of lying—self-promotional deception—which involves trying to look good in the eyes of others. Given that we found that low-power individuals have a higher desire for status, it makes sense that low power increases self-promotional lying.

As an exploratory analysis, we examined the association between sex/gender difference effect sizes and power difference effect sizes for Level I constructs wherever there was an exact match. This analysis revealed a significant positive association between power and sex/gender difference effect sizes, *r*(34) = 0.41, *P* = 0.012. This correlation should be interpreted with caution because of the lack of stability caused by the relatively low *N* and upward bias in the power effect sizes given that we only included significant power effects in our analysis. However, this positive correlation is a notable contrast to the correlation between extraversion and sex/gender difference effect sizes, which was nonsignificant and opposite in sign, *r*(35) = −0.25, *P* = 0.128. These correlations provide additional empirical support for the hypothesized connection between sex/gender differences and power differences.

### Limitations and future directions

One limitation of the current research is that our mapping of power to sex/gender differences is inherently correlational. Nonetheless, we believe this research offers a framework to conduct more specific and focused causal tests of the relationship between power and sex differences that we have observed. One analysis we hoped to conduct was to test whether sex/gender differences are weaker in high-power conditions. We coded every power experiment for whether the researchers tested an interaction between power and sex/gender and found that <5% did so. Thus, we did not have enough statistical power to adequately explore this possibility. Future research should conduct sex/gender stratified experiments to directly test whether the psychological experience of power moderates documented sex/gender differences.

We acknowledge that potential biases could have been introduced at different points of the research process. For example, our own theoretical predispositions or cultural perspectives may have constrained the set of constructs that we considered and analyzed. Similarly, although we took precautions to mitigate against author bias in the classifications of the power and sex/gender differences by having independent judges categorize constructs, it is possible that implicit theories about power and sex/gender held by the judges could have influenced their decisions. Furthermore, our reliance on meta-analyses could have amplified any sampling biases, cultural biases, or methodological limitations present in the original studies. Authors of meta-analyses should both highlight the methodological limitations of their underlying data and offer a roadmap for future research to use more rigorous methods. Broadening the diversity of the research team can also increase the cultural perspectives and insights brought to bear on the research question.

Another limitation of the current research is that we purposely limited our scope to significant power effects, i.e. we only explored a sex/gender difference for constructs that have been affected by power manipulations in published experiments. Thus, we are not claiming that every sex/gender difference is driven, even in part, by power differences, nor are we arguing that all the variance in sex/gender differences is accounted for by power. Rather, we have offered one of the most comprehensive cataloging of evidence to date that social power is likely one important explanatory factor that has contributed to observed differences between men and women. Future research should select known sex/gender differences that have not been explored in the power literature and causally test whether experimental manipulations of power produce differences that correspond with the documented sex-gender differences. For example, researchers of the effects of power on cognitive processes have focused on male-favoring cognitive differences; it is important for future research on power to explore female-favoring cognitive differences (e.g. in the verbal domain). Similarly, researchers can explore how power affects the varied documented forms of sex/gender differences in risk-seeking (70, 71).

Future work could also examine the conditions under which individuals display the characteristics or propensities that are typically associated with the opposite sex/gender or power profile. For example, Eagly (72) highlights that women are more likely than men to take risky action to help others in communal situations (e.g. women are more likely to be living organ donors and were more likely to rescue Jews during the Holocaust). More generally, future work could consider when and why individuals exhibit certain cognitive and behavioral propensities that are associated with vs. opposite their sex/gender or power level.

## Conclusion

Recognizing the potential influence of power in the interpretation of sex/gender differences is significant not only for theory but also for practice. As many scholars have long argued, underappreciating the role of power in observed sex/gender differences risks adding false credence to sex-gendered expectations for behavior and reinforces inequality between men and women (16, 48, 73). Moreover, disentangling the role of power from biological sex is critical to theorizing about intersectional effects involving sex/gender on social behavior (74). A clearer understanding of how power contributes to sex/gender differences is essential for producing truly level playing fields for men and women.

## Methods

This section details the steps we took that are outlined in our flow chart in Fig. 1 and provides additional detail to the Overview and analytic set-up section.

### Identifying the effects of power

To supplement the power effects identified in the two review papers (41, 52), we performed a targeted search for experimental papers on power published in 2017–2023 (i.e. since these reviews) and located 42 such papers with clearly identifiable high- and low-power conditions to be considered further for inclusion (see Table S1). Moreover, we used a snowballing approach from the papers previously identified to locate 18 additional papers on power that met the same requirements for consideration. Because we were interested in the demonstrated effects of power, we only included experiments with significant results (25 experiments were excluded for nonsignificant results), similar to a *P*-curve approach. In addition, three authors reviewed each paper and excluded experiments based on the criteria in Fig. 1. Below we list the reasons why statistically significant effects of power were excluded (see Table S3). We excluded:

- studies that did not include manipulations of participants’ power, because we were interested in the causal effects of power (*k* = 144);
- studies that did not include both a high-power and a low-power condition, because we wanted to align with the comparison between men and women (*k* = 72); similarly, studies that did not include both men and women (*k* = 10);
- studies that manipulated status or leadership as much as, or more than, power (i.e. the manipulation of power was conflated with leadership/status), because we were specifically interested in the effects of power (*k* = 5);
- studies that manipulated power physically (e.g. through posture), because these manipulations do not conform to the definition of power that we use in the current research (*k* = 5);
- studies in which participants worked in a group or team, because we were interested in the effects of power on individual psychological propensities and tendencies and not on group processes (*k* = 12);
- studies based on evaluations/assessments exclusively by others, because these may reflect stereotypes and expectations and not the effects of power on individual psychological propensities and individual tendencies (*k* = 1);
- studies based on samples of romantic relationships or measures of sexual behavior, because evolutionary explanations are equally plausible for these effects (*k* = 22);
- studies based on neuroimaging or psychophysiological measures, because evolutionary explanations are equally plausible for these effects (*k* = 6); and
- studies for which an editor expressed concern about the research (*k* = 10).

For the included experiments, we typically extracted one effect comparing the high- to the low-power condition. If measures of multiple constructs were reported, we tried to extract separate effects for each construct if those constructs belonged to different Level II constructs to avoid issues of nonindependence. If repeated measures of the same construct were taken at multiple time points, we extracted the measure at the first time point if it was available, or otherwise took the average across time points. With two exceptions, the extracted effects involved no independent variables other than high- vs. low-power. The first exception was when the low-power condition was conflated with a control/baseline condition in the reporting of statistics; in this case, we extracted the effect by comparing the combination of those conditions to the high-power condition. Also, in factorial designs, we extracted a statistically significant main effect of power, or, failing that, a statistically significant simple effect of power within one level (or combination of levels) of one of the other independent variables (e.g. within an “active goal” rather than an “inactive goal” condition of an experiment investigating the effect of power on goal pursuit, or within a “stable power” rather than an “unstable power” condition of an experiment investigating the effect of stability on power). Ninety-one experiments were excluded because it was not possible to derive either a main effect of power or a simple effect that could reasonably be expected to generalize beyond the specific condition of another independent variable.

For the effects extracted, we recorded the dependent measure(s), the direction of the effect(s), sample size (*n*), and effect size(s), if provided (*d*, *η*^2^, *g*, *Φ*, *r*, odds ratio). If the effect size was not provided (which was true in the majority of experiments), we recorded an inferential statistic value (*F*, *t*, *χ*^2^, or *z*) and *df*s; if the test statistic and *df*s were not provided, we recorded *M*s and SDs. For those experiments that did not provide an effect size, we used the remaining meta-data to compute Cohen's *d* using (https://lbecker.uccs.edu/) and https://www.psychometrica.de/effect_size.html, assuming equal *n* across conditions when sample size information within condition was missing (which was true in nearly all cases). For one effect, the meta-data provided was inadequate to compute a precise effect size, and we approximated this effect size from a standardized regression coefficient, which could be done because it was between −0.5 and +0.5 (75). Thirteen experiments were excluded for providing inadequate statistics from which to derive an effect size. We then converted all effect sizes to *d* (except for Hedges’ *g*, which was treated like *d*) as needed using the effect size calculators at https://www.psychometrica.de/effect_size.html.

### Categorizing the effects of power

We used an iterative coding process to initially categorize the power effects. Two co-authors reviewed the effects and created initial groupings of similar findings (e.g. findings on “conformity” and “ability to be persuaded” were grouped together under “susceptibility to influence”). Next, three other co-authors discussed the initial categories to gain agreement on which power effects should be grouped together before beginning the comparative analysis with the sex/gender difference literature. Through this process, we consolidated and recombined power constructs until we had 69 effects (for a complete list of included effects, see Table S2). We computed the sample-size-weighted average *d* for 50 of these 69 effects and obtained a single-study *d* for the remaining 19 effects. These effect sizes should be treated with caution because all of them are based on statistically significant effects (i.e. they omit null effects) and most of them are based on low *N* or *k* (i.e. *N* ≤ 500 and/or *k* ≤ 2), which are minimal thresholds according to Wilmot et al. (69). Thus, the effect sizes are likely unstable and biased upwards. To conduct our *P*-curve analysis, we followed the official user guide by Simonsohn et al. (76) which can be found at *p*-curve.com.

### Identifying sex/gender differences

As noted in Fig. 1, we began our search using the Archer meta-analysis on sex differences and then searched additional sex/gender difference meta-analyses. We excluded all sex/gender meta-analyses that met any of the exclusion criteria for the power experiments. To ensure comparability to the power literature we also excluded meta-analyses involving the following: (i) measures of physical harm or sexual violence, (ii) measures related to sexuality, (iii) Big-Five measures of personality, (iv) measures closely related to clinical outcomes (e.g. coping behaviors, depressive symptoms), (v) a sample majority of clinical patients, (vi) a sample majority of children and/or adolescents, or (vii) a sample majority of older adults (i.e. >59 years of age). In some cases, we were able to drop clinical, child, adolescent, and/or older adult samples and recompute effect sizes so that we did not have to exclude a meta-analysis that otherwise met our criteria (see Table S6 for details about these and other modified effect sizes). For meta-analyses that provided multiple effect sizes, we took the effect size for the broadest construct(s), unless the disaggregated effect sizes provided a better construct match with an effect of power or were substantially different in magnitude or sign. In cases where separate effect sizes were reported for different raters of the same construct (e.g. self- and supervisors’ ratings of employees’ work behavior), we averaged effect sizes across raters. For constructs with multiple meta-analyses that satisfied the criteria above, we took the effect size from the most recent meta-analysis, unless the meta-analyses were based on nonoverlapping sets of papers. In these instances, the meta-analytic effect sizes were weighted by the number of studies (*k*) and averaged (see Table S6). This process yielded 126 sex/gender difference meta-analytic effect sizes to be considered for inclusion (these include 59 of the 140 unique sex/gender constructs published by Archer (5)).

### Categorizing constructs to compare power with sex/gender differences

Given that there was not a 1:1 match between most of the power constructs and sex/gender constructs, we created broader categories to categorize the effect-level constructs (called Level I constructs) from the power and sex/gender difference literatures. These higher-level constructs (called Level II constructs) allowed us to compare the power effects with sex/gender differences. To generate these constructs, the authors used the 149 unique Level I constructs identified for inclusion from the power and sex/gender difference searches (i.e. duplicates from both power and sex/gender were removed). An iterative process involving all the authors produced 11 Level II constructs, into which we expected the 149 Level I constructs could be categorized by independent judges. For ease of presentation and to further connect conceptually related power effects and corresponding sex/gender differences, we grouped the Level II constructs into even broader categories called Level III categories. We developed these four level III categories based on how the power and sex/gender literatures were conceptualized in the following sources: (i) the review of power by Galinsky et al. (41), (ii) the meta-synthesis by Archer (5) of sex/gender difference meta-analyses and large-*N* studies, and (iii) a review linking both sex/gender and power to agency and communion (43, 77, 78). See Table S10 for the definitions of Level II constructs and their nesting within the Level III categories of agency, communion, self-evaluation, and cognitive processes.

Next, independent judges categorized Level I into Level II constructs. To facilitate this process, we gave each Level I constructed a definition that would be understandable to an expert in the field of either social psychology or organizational behavior (i.e. field experts). We used ChatGPT-4 to develop these definitions using the following form of a query, “How is <Level I construct> defined in social psychology?”. The last author used these results to write definitions for a trial set of 10 constructs and compared these to definitions in highly cited articles. The ChatGPT-4 generated definitions proved to be accurate, so we utilized ChatGPT-4 to generate definitions for all power and sex/gender Level I constructs. For Level II construct definitions, three co-authors worked iteratively to develop definitions to be used by field experts in categorizing Level I constructs (see Table S10 for these definitions).

Two field experts, along with the last author, served as the independent judges. The two field experts both had a PhD in social psychology/organizational behavior but were not authors of this work and were blind to the goals of this project. Working with the Level I constructs and their definitions and with the Level II constructs and their definitions (and their nesting within Level III categories), the field experts independently categorized Level I into Level II constructs, with the option to use the “other” category provided there was inadequate fit with any of the Level II constructs.

At least two of the three experts agreed on 91.3% of cases; there was consensus on 44.9% (67/149), which included seven constructs assigned to “other”, and two of three experts agreed on 46.3% (69/149). Among the constructs about which the experts did not all agree, 34.1% (28/82) were placed in the same Level III category by all three experts (i.e. they agreed at a broader level), and another 35.4% (29/82) involved at least one expert selecting the “other” category. These results show that there was substantial agreement in categorizing the constructs, and disagreements did not tend to involve Level III category disagreements (e.g. one expert deciding a construct belonged under agency, and another expert deciding it belonged under communion). We sought resolution to the disagreements by reviewing the literature or matching the text of the Level I construct definition with the text of a Level II construct definition; this resulted in resolutions for 62.2% (51/82) of the constructs for which there had not been consensus (see Table S8). Of the remaining 31 constructs, 5 were resolved by other means (e.g. judges’ majority, similarity to another construct with a clear resolution), and 26 could not be categorized (i.e. were assigned to “other” along with the seven constructs that were consensually assigned to “other”). Thus, a total of 33 constructs were excluded from further analysis.

### Extraversion effects

For any constructs that matched power or sex/gender Level I constructs, we adopted the Level II categorization assigned previously; we also excluded any constructs that matched those that had been deemed uncategorizable in our Level II constructs from the power and sex/gender differences. Then, two authors served as judges and independently attempted to categorize all the remaining constructs following the same rules used for power and sex/gender differences. Of the 113 viable constructs that did not have an exact match with a power or sex/gender construct, the judges agreed on 86, including 51 they both assigned to “other” (a large number of other assignments is due to the fact that many constructs were specific either to the workplace or to college undergraduate experiences), and disagreed on only 27. To resolve these disagreements, another author independently categorized these constructs, thus serving as a third judge and “tiebreaker.” Twenty-four of these constructs were assigned to a Level II construct agreed on by two judges, and three constructs about which all three judges disagreed were deemed uncategorizable (i.e. assigned to “other”; see Table S9). Thus, a total of 54 constructs could not be categorized and were excluded from further analysis. As with sex/gender difference effects, in some cases, we recomputed extraversion effects after excluding samples or measures that followed our exclusion rules (see Table S6).

## Supplementary Material

pgae025_Supplementary_Data

## Data Availability

All information needed to reproduce the results is contained in figures and supplementary tables.

## Note

a While most prior psychological research has studied sex/gender in a binary way, binary conceptions of sex/gender are increasingly being challenged by science, as well as society (1). Although we apply the same categorical scheme frequently used in the literature, we also recognize that sex/gender constructs are more continuous in nature.

## References

[1] Hyde JS, Bigler RS, Joel D, Tate CC, van Anders SM. 2019. The future of sex and gender in psychology: five challenges to the gender binary. Am Psychol. 72:171–193. 10.1037/amp0000307 30024214

[2] Hyde JS . 2005. The gender similarities hypothesis. Am Psychol. 60:581–592. 16173891 10.1037/0003-066X.60.6.581

[3] Hyde JS . 2014. Gender similarities and differences. Annu Rev Psychol. 65:373–398. 23808917 10.1146/annurev-psych-010213-115057

[4] Zell E, Krizan Z, Teeter SR. 2015. Evaluating gender similarities and differences using metasynthesis. Am Psychol. 70:10–20. 25581005 10.1037/a0038208

[5] Archer J . 2019. The reality and evolutionary significance of human psychological sex differences. Biol Rev. 94:1381–1415. 30892813 10.1111/brv.12507

[6] Filippin A, Crosetto P. 2016. A reconsideration of gender differences in risk attitudes. Manag Sci. 62:3138–3160.

[7] Voyer D, Voyer S, Bryden MP. 1995. Magnitude of sex differences in spatial abilities: a meta-analysis and consideration of critical variables. Psychol Bull. 117:250–270. 7724690 10.1037/0033-2909.117.2.250

[8] Eagly AH, Carli LL. 1981. Sex of researchers and sex-typed communications as determinants of sex differences in influenceability: a meta-analysis of social influence studies. Psychol Bull. 90:1–20.

[9] Herlitz A, Lovén J. 2013. Sex differences and the own-gender bias in face recognition: a meta-analytic review. Vis Cogn. 21:1306–1336.

[10] Thompson AE, Voyer D. 2014. Sex differences in the ability to recognise non-verbal displays of emotion: a meta-analysis. Cogn Emot. 28:1164–1195. 24400860 10.1080/02699931.2013.875889

[11] Buss DM, Kendrick DT. 1998. Evolutionary social psychology. In: Gilbert DT, Fiske ST, Lindzey G, editors. The handbook of social psychology. New York City (NY): McGraw-Hill. p. 982–1026.

[12] Trivers RL . 1972. Parental investment and sexual selection. In: Campbell B, editor. Sexual Selection and the descent of man: the Darwinian pivot. Chicago (IL): Transaction Publishers. p. 136–179.

[13] Daly M, Wilson M. 1983. Sex, evolution, and behavior. 2nd ed. Belmont (CA): Wadsworth.

[14] Pellegrini AD, Archer J. 2005. Sex differences in competitive and aggressive behavior: a view from sexual selection theory. In: Ellis BJ, Bjorklund DF, editors. Origins of the social mind: evolutionary psychology and child development. New York (NY): The Guilford Press. p. 219–244.

[15] Wilson M, Daly M. 1985. Competitiveness, risk taking, and violence: the young male syndrome. Ethol Sociobiol. 6:59–73.

[16] Eagly AH, Wood W. 2013. The nature–nurture debates: 25 years of challenges in understanding the psychology of gender. Perspect Psychol Sci. 8:340–357. 26172976 10.1177/1745691613484767

[17] Ridgeway CL . 2011. Framed by gender: how gender inequality persists in the modern world. New York (NY): Oxford University Press.

[18] Lorber J . 1994. Paradoxes of gender. New Haven (CT): Yale University Press.

[19] Ely R, Padavic I. 2007. A feminist analysis of organizational research on sex differences. Acad Manag Rev. 32:1121–1143.

[20] West C, Zimmerman DH. 1987. Doing gender. Gender Soc. 1:125–151.

[21] Eagly AH, Wood W. 1999. The origins of sex differences in human behavior: evolved dispositions versus social roles. Am Psychol. 54:408–423.

[22] Buss DM . 1995. Psychological sex differences: origins through sexual selection. Am Psychol. 50:164–168. 7726470 10.1037/0003-066x.50.3.164

[23] Geary DC . 2010. Male, female: the evolution of human sex differences. 2nd ed. Washington (DC): American Psychological Association.

[24] Deaux K, Lafrance M. 1998. Gender. In: Gilbert DT, Fiske ST, Lindzey G, editors. The handbook of social psychology. New York (NY): McGraw-Hill. p. 788–827.

[25] Eagly AH, Wood W. 2012. Social role theory. In: van Lange PAM, Kruglanski AW, Higgins ET, editors. Handbook of theories of social psychology. Thousand Oaks (CA): SAGE Publications Ltd. p. 458–476.

[26] Reis HT, Carothers BJ. 2014. Black and white or shades of gray. Curr Dir Psychol Sci. 23:19–26.

[27] Joel D, et al 2015. Sex beyond the genitalia: the human brain mosaic. Proc Natl Acad Sci U S A. 112:15468–15473. 26621705 10.1073/pnas.1509654112 PMC4687544

[28] Del Giudice M . 2021. Binary thinking about the sex binary: a comment on Joel (2021). Neurosci Biobehav Rev. 127:144–145. 33901499 10.1016/j.neubiorev.2021.04.020

[29] Ridgeway CL, Smith-Lovin L. 1999. The gender system and interaction. Annu Rev Sociol. 25:191–216.

[30] Fiske ST, Berdahl H. 2007. Social power. In: Kruglanski AW, Higgins ET, editors. Social psychology: handbook of basic principles. New York (NY): The Guilford Press. p. 678–692.

[31] Magee JC, Galinsky AD. 2008. Social hierarchy: the self-reinforcing nature of power and status. Acad Manag Ann. 2:351–398.

[32] Ridgeway CL, Diekema D. 1992. Are gender differences status differences? In: Ridgeway CL, editor. Gender, interaction, and inequality. New York (NY): Springer-Verlag. p. 157–180.

[33] U.S. Department of Labor . 2023. Equal pay day 2023: Department of labor initiatives seek to close gender, racial wage gap, increase equity in federal programs. US Department of Labor. https://www.dol.gov/newsroom/releases/osec/osec20230314.

[34] Jochecová K . 2023. EU parliament moves to end gender pay gap—this time for good. Politico. https://www.politico.eu/article/eu-parliament-end-gender-pay-gap/.

[35] Robinson L, James N. 2023. Women's power index. Council on Foreign Relations. https://www.cfr.org/article/womens-power-index.

[36] Hinchliffe E . 2023. Women ceos run 10.4% of Fortune 500 companies. A quarter of the 52 leaders became CEO in the last year. Fortune. https://fortune.com/2023/06/05/fortune-500-companies-2023-women-10-percent/.

[37] Deloitte . 2022. Women in the boardroom, 2022 update. Deloitte. https://www2.deloitte.com/us/en/insights/topics/leadership/women-in-the-boardroom.html.

[38] Lodewick C . 2022. Female executives only control 1% of shares at America's biggest companies—and sometimes less, depending on the day. Fortune. https://fortune.com/2022/06/07/female-executives-compensation-execushe-gender-gap/.

[39] Blumberg RL . 1984. A general theory of gender stratification. Sociol Theory. 2:23–101.

[40] Sidanius J, Pratto F. 1999. Social dominance: an intergroup theory of social hierarchy and oppression. New York (NY): Cambridge University Press.

[41] Galinsky AD, Rucker DD, Magee JC. 2015. Power: past findings, present considerations, and future directions. In: Simpson JA, Dovidio JF, editors. APA Handbook of personality and social psychology. Washington (DC): American Psychological Association. p. 421–460.

[42] Keltner D, Gruenfeld DH, Anderson C. 2003. Power, approach, and inhibition. Psychol Rev. 110:265–284. 12747524 10.1037/0033-295x.110.2.265

[43] Rucker DD, Galinsky AD, Magee JC. 2018. The agentic-communal model of advantage and disadvantage: how inequality produces similarities in the psychology of power, social class, gender and race. In: Olson JM, editor. Advances in experimental social psychology. Cambridge (MA): Academic Press Inc. p. 71–125.

[44] Sidanius J, Levin S, Federico CM, Pratto F. 2001. Legitimizing ideologies: the social dominance approach. In: Jost JT, Major B, editors. The psychology of legitimacy: emerging perspectives on ideology, justice, and intergrouop relations. Cambridge (UK): Cambridge University Press. p. 307–331.

[45] Berger J, Rosenholtz SJ, Zelditch M. 1980. Status organizing processes. Annu Rev Sociol. 6:479–508.

[46] Hall DT . 1972. A model of coping with role conflict: the role behavior of college educated women. Adm Sci Q. 17:471.

[47] Meeker BF, Weitzel-O’Neill PA. 1977. Sex roles and interpersonal behavior in task-oriented groups. Am Sociol Rev. 42:91. 900658

[48] Rudman LA, Glick P. 2021. The social psychology of gender: how power and intimacy shape gender relations. New York (NY): Guilford Press.

[49] Magee JC, Smith PK. 2013. The social distance theory of power. Pers Soc Psychol Rev. 17:158–186. 23348983 10.1177/1088868312472732

[50] Simonsohn U, Nelson LD, Simmons JP. 2014. P-curve: a key to the file-drawer. J Exp Psychol Gen. 143:534–547. 23855496 10.1037/a0033242

[51] Simonsohn U, Nelson LD, Simmons JP. 2014. P-curve and effect size: correcting for publication bias using only significant results. Perspect Psychol Sci. 9:666–681. 26186117 10.1177/1745691614553988

[52] Guinote A . 2017. How power affects people: activating, wanting, and goal seeking. Annu Rev Psychol. 68:353–381. 27687123 10.1146/annurev-psych-010416-044153

[53] Funder DC, Ozer DJ. 2019. Evaluating effect size in psychological research: sense and nonsense. Adv Methods Pract Psychol Sci. 2:156–168.

[54] Martell RF, Lane DM, Emrich C. 1996. Male-female differences: a computer simulation. Am Psychol. 51:157–158.

[55] Abele AE, Uchronski M, Suitner C, Wojciszke B. 2008. Towards and operationalization of fundamental dimensions of agency and communion: trait content ratings in five countries considering valence and frequency of word occurrence. Eur J Soc Pscyhol. 38:1202–1217.

[56] Eagly AH, Crowley M. 1986. Gender and helping behavior: a meta-analytic review of the social psychological literature. Psychol Bull. 100:283–308. 38376350 10.1037/0033-2909.100.3.283

[57] Darley JM, Latané B. 1968. Bystander intervention in emergencies: diffusion of responsibility. J Pers Soc Psychol. 8:377–383. 5645600 10.1037/h0025589

[58] Hershcovis MS, et al 2017. Witnessing wrongdoing: the effects of observer power on incivility intervention in the workplace. Organ Behav Hum Decis Process. 142:45–57.

[59] McCrae RR, Costa PT. 2008. Empirical and theoretical status of the five-factor model of personality traits. In: Boyle GJ, Matthews G, Saklofske DH, editors. The SAGE handbook of personality theory and assessment. Thousand Oaks (CA): Sage Publications Inc. p. 273–294.

[60] DesJardins NML, Srivastava S, Küfner ACP, Back MD. 2015. Who attains status? Similarities and differences across social contexts. Soc Psychol Pers Sci. 6:692–700.

[61] Anderson C, John OP, Keltner D, Kring AM. 2001. Who attains social status? Effects of personality and physical attractiveness in social groups. J Pers Soc Psychol. 81:116–132. 11474718 10.1037//0022-3514.81.1.116

[62] Lodder P, Ong HH, Grasman RPPP, Wicherts J. 2019. A comprehensive meta-analysis of money priming. J Exp Psychol Gen. 148:688–712. 30973262 10.1037/xge0000570

[63] Stajkovic AD, Greenwald JM, Stajkovic KS. 2022. The money priming debate revisited: a review, meta-analysis, and extension to organizations. J Organ Behav. 43:1078–1102.

[64] Anderson C, Spataro SE, Flynn FJ. 2008. Personality and organizational culture as determinants of influence. J Appl Psychol. 93:702–710. 18457498 10.1037/0021-9010.93.3.702

[65] Ensari N, Riggio RE, Christian J, Carslaw G. 2011. Who emerges as a leader? Meta-analyses of individual differences as predictors of leadership emergence. Pers Individ Dif. 51:532–536.

[66] Lippa RA . 2010. Sex differences in personality traits and gender-related occupational preferences across 53 nations: testing evolutionary and social-environmental theories. Arch Sex Behav. 39:619–636. 18712468 10.1007/s10508-008-9380-7

[67] Weisberg YJ, DeYoung CG, Hirsh JB. 2011. Gender differences in personality across the ten aspects of the Big Five. Front Psychol. 2:178. 21866227 10.3389/fpsyg.2011.00178 PMC3149680

[68] DeYoung CG, Quilty LC, Peterson JB. 2007. Between facets and domains: 10 aspects of the Big Five. J Pers Soc Psychol. 93:880–896. 17983306 10.1037/0022-3514.93.5.880

[69] Wilmot MP, Wanberg CR, Kammeyer-Mueller JD, Ones DS. 2019. Extraversion advantages at work: a quantitative review and synthesis of the meta-analytic evidence. J Appl Psychol. 104:1447–1470. 31120263 10.1037/apl0000415

[70] Becker SW, Eagly AH. 2004. The heroism of women and men. Am Psychol. 59:163–178. 15222859 10.1037/0003-066X.59.3.163

[71] Rankin LE, Eagly AH. 2008. Is his heroism hailed and hers hidden? Women, men, and the social construction of heroism. Psychol Women Q. 32:414–422.

[72] Eagly AH . 2022. Women take risks to help others to stay alive. Behav Brain Sci. 45:e135. 35875969 10.1017/S0140525X22000437

[73] Jackman M . 1994. The velvet glove: paternalism and conflict in gender, class, and race relations. Berkeley (CA): University of California Press.

[74] Crenshaw K . 2017. On intersectionality: essential writings. New York (NY): The New Press.

[75] Peterson RA, Brown SP. 2005. On the use of beta coefficients in meta-analysis. J Appl Psychol. 90:175–181. 15641898 10.1037/0021-9010.90.1.175

[76] Simonsohn U, Simmons JP, Nelson LD. 2015. Better P-curves: making P-curve analysis more robust to errors, fraud, and ambitious P-hacking, a reply to Ulrich and Miller (2015). J Exp Psychol Gen. 144:1146–1152. 26595842 10.1037/xge0000104

[77] Hsu N, Badura KL, Newman DA, Speach MEP. 2021. Gender, “masculinity,” and “femininity”: a meta-analytic review of gender differences in agency and communion. Psychol Bull. 147:987–1011.

[78] Rucker DD, Galinsky AD. 2016. The agentic-communal model of power: implications for consumer behavior. Curr Opin Psychol. 10:1–5.

