# Supplementary material for: Are many sex/gender differences really power differences?
Source: PNAS Nexus. 2024 Feb 27;3(2):pgae025. doi: 10.1093/pnasnexus/pgae025 (PMC10898859; doi:10.1093/pnasnexus/pgae025)
Supplement: pgae025_Supplementary_Data [file pgae025_supplementary_data.pdf]

SUPPLEMENT

Table of Contents

SI1. Search terms used for identifying studies on power, gender, or extraversion

SI2. Power experiments included in P-curve analysis and comparison to sex/gender meta-analytic effects.

SI3. Excluded power studies with reason for exclusion

SI4. Constructs relabeled from Archer (2019)

SI5. Constructs relabeled from Wilmot et al. (2019)

SI6. Effect size modification procedures for constructs [with any new labels] from sex/gender and extraversion meta-analyses

SI7. Comparison of extraversion effect sizes and sex/gender differences from meta-analyses

SI8. Level II categorization resolutions for power and sex/gender Level I constructs without consensus

SI9. Level II categorization resolutions for extraversion Level I constructs without consensus

SI10. Complete list of Level I and Level II constructs and definitions

SI11. Key PRISMA guidelines: Actions taken, or reason step not applicable

Table SI1. Search terms used for identifying studies on power, gender, or extraversion

Independent Variable	Reason for Search	Search Strategy
Power	To address right year-of-publication truncation at 2017 for papers identified in reference lists of Galinsky, Rucker, & Magee (2015) and Guinote (2017)	On May 11, 2023, the following search was conducted for years 2017-2023<br><br>Databases: ProQuest Social Science Database including: APA PsycArticles, APA PsycInfo, Applied Social Sciences Index & Abstracts (ASSIA), International Bibliography of the Social Sciences (IBSS), Psychology Database, Research Library: Social Sciences, Social Science Database<br><br>Search terms/logic: ("manipulate authority" OR "manipulate power" OR "manipulate rank" OR "manipulate social power" OR "manipulate powerlessness" OR "manipulated authority" OR "manipulated power" OR "manipulated rank" OR "manipulated social power" OR "manipulated powerlessness" OR "manipulation of authority" OR "manipulation of power" OR "manipulation of rank" OR "manipulation of social power" OR "manipulation of powerlessness" OR "authority was manipulated" OR "power was manipulated" OR "rank was manipulated" OR "social power was manipulated" OR "powerlessness was manipulated" AND at.exact("Article") AND stype.exact("Scholarly Journals" NOT ("Historical Newspapers" OR "Magazines" OR "Trade Journals" OR "Newspapers" OR "Conference Papers & Proceedings" OR "Historical Periodicals" OR "Other Sources" OR "Reports" OR "Wire Feeds" OR "Working Papers")))<br><br>Between May 11 and September 18, 2023, Google Scholar was monitored for newly published papers using similar search terms.
Sex/Gender	To identify sex/gender meta-analytic effects that could be compared to power effects	In Google Scholar: (“meta analysis” OR “meta analytic” OR “meta synthesis”) AND ((gender OR sex) OR (men AND women) OR (male AND female)) AND <Level I power construct label><br><br>After the Level II constructs were finalized, this search was conducted again, except that the final term was replaced with <Level II construct label><br><br>Stopping rule applied: after 100 consecutive records with no hits<br>Last searched: September 18, 2023
Extraversion	To identify extraversion meta-analytic effects that	In Google Scholar: (“meta analysis” OR “meta analytic” OR “meta synthesis”) AND (extraversion OR extroversion OR personality OR neo OR “five factor” OR “big five” OR hexaco) AND <Level I gender construct label>

	could be compared to sex/gender effects	After cycling through all Level I power constructs, this search was conducted again, except that the final term was replaced with <Level I power construct label>, and then again with <Level II construct label><br><br>Stopping rule applied: after 100 consecutive records with no hits<br>Last searched: September 18, 2023

Table SI2. Power experiments included in P-curve analysis and comparison to sex/gender meta-analytic effects

Level I Construct	Source Authors	Source Year	Study #	Dependent Measure	*n*	*d*
**Included in P-curve and in sex/gender effect comparison**						
Abstract thinking	Joshi et al.	2020	5	Abstract communication	267	0.29
Abstract thinking	Miyamoto & Ji	2011	1	Use of adjectives to describe one's partner	35	0.58
Abstract thinking	Miyamoto & Ji	2011	2	Use of taxonomic vs. thematic categorization	37	0.68
Abstract thinking	Schmid & Amodio	2021	2	Recognition of upright stimuli	140	0.46
Abstract thinking	Smith & Trope	2006	1	Number of weak (non-exemplar) items included during categorization	49	0.73
Abstract thinking	Smith & Trope	2006	2	Number of high-level responses	51	0.63
Abstract thinking	Smith & Trope	2006	3	Discriminability	53	0.57
Abstract thinking	Smith & Trope	2006	4	Recognition test (critical lures)	151	0.56
Abstract thinking	Smith & Trope	2006	5	Identification of random pictures	187	0.62
Abstract thinking	Smith & Trope	2006	6	Number of correct responses on Embedded Figures Task	84	0.49
Action orientation	Blader & Chen	2012	5	Make the first offer	208	1.08
Action orientation	Guinote	2007b	1	Time to make a decision	99	0.56
Action orientation	Guinote	2007b	4	Early responses	68	0.50
Action orientation	Guinote	2007b	2	Initiated action time	35	0.99
Action orientation	Hyun & Ku	2020	2	Proactive coping score (preemptively preventing a problem)	143	0.37
Action orientation	Jiang et al.	2014	4	Consumer switching	80	0.49
Action orientation	Jiang et al.	2014	1a	Consumer switching	149	0.38
Action orientation	Jiang et al.	2014	1b	Consumer switching	106	0.42
Action orientation	Jiang et al.	2014	2	Consumer switching	131	0.38
Action orientation	Jiang et al.	2014	3	Consumer switching	100	0.44
Action orientation	Jiang et al.	2014	5	Consumer switching	114	0.73
Action orientation	Magee et al.	2007	3	Make the first offer	132	0.42
Action orientation	Magee et al.	2007	4	Make the first offer	62	0.76
Action orientation	Rucker et al.	2014	3	Propensity to take card in blackjack	81	0.48

Action orientation	Scholl & Sassenberg	2015	1	Prefactual thoughts	42	0.66
Action orientation	Scholl & Sassenberg	2015	2	Prefactual thoughts	56	1.00
Action orientation	Scholl & Sassenberg	2015	3	Prefactual thoughts	101	0.52
Action orientation	Smith & Bargh	2008	2	Movement speed	59	0.58
Action orientation	Galinsky et al.	2003	1	Propensity to take card in blackjack	32	0.76
Action orientation	Galinsky et al.	2003	2	Propensity to remove aversive stimulus	59	0.55
Action orientation	Galinsky et al.	2003	3	Take from commons/give to public good	103	0.54
Affiliation motivation	Case et al.	2015	1	Interest in joining social networking group	140	-0.57
Affiliation motivation	Copeland	1994	only study	Concern with getting along with targets	48	-0.79
Affiliation motivation	Liu et al.	in press	2	Need to belong	349	-0.48
Affiliation motivation	Liu et al.	in press	3	Desire for inclusion of other in self	110	-0.71
Affiliation motivation	Liu et al.	in press	4	Desire for inclusion of other in self	500	-0.28
Affiliation motivation	Liu et al.	in press	5	Need to belong	140	-0.62
Affiliation motivation	Waytz et al.	2015	5	Need to belong	285	-0.25
Affiliation motivation	Waytz et al.	2015	6	Need to belong	404	-0.60
Anger-related emotions	Mooijman et al.	2020	3	Anger	182	-0.55
Anger-related emotions	Petkanopoulou et al.	2019	1	Direct anger expression	193	0.82
Anger-related emotions	Petkanopoulou et al.	2019	2	Anger expression	117	0.41
Anger-related emotions	Struthers et al.	2019	2	Decision to harbor a grudge	181	-0.68
Anxiety-related emotions	Cai & Wu	2017	2	Fear of negative evaluation	136	-0.59
Anxiety-related emotions	Pai et al.	2021	1	State attachment anxiety	525	-0.19
Anxiety-related emotions	Petkanopoulou et al.	2019	1	Negative social appraisals about one's relationship with the target of anger	195	-0.61
Assertiveness	Schaerer et al.	2018	Sample 1	Inclination to assert oneself first in interaction	449	1.12
Assertiveness	Schaerer et al.	2018	Sample 2	Inclination to assert oneself first in interaction	451	0.70
Auditory selective attention	DeWall et al.	2011	1a	Dichotic listening performance	33	0.77
Auditory selective attention	DeWall et al.	2011	2	Dichotic listening performance	75	0.87
Authenticity	Anderson & Berdahl	2002	2	Expression of true attitudes and opinions	130	0.46

Authenticity	Kifer et al.	2013	2a	State authenticity	252	0.24
Authenticity	Kraus et al.	2011	3	Authenticity	130	0.43
Competitiveness	De Dreu & Van Kleef	2004	3	Negotiator demands	217	0.36
Competitiveness	Tost et al.	2012	3	Competitiveness	133	0.63
Competitiveness	Zhong & Li	2023	1a	Various covert competitive behaviors	145	-0.35
Competitiveness	Zhong & Li	2023	1b	Various covert competitive behaviors	160	-0.35
Confidence	Briñol et al.	2007	1	Confidence in own attitudes	80	0.57
Confidence	Briñol et al.	2007	4	Confidence in own thoughts	68	0.61
Confidence	Fast et al.	2012	1	Self-assigned confidence intervals to own decisions	41	0.65
Confidence	Fast et al.	2012	2	Overconfidence	160	0.28
Confidence	Fast et al.	2012	3	Confidence in own decisions	102	0.34
Confidence	Lammers et al.	2017	4	Confidence in high ease of retrieval conditions	202	0.55
Confidence	See et al.	2011	3	Confidence in accuracy of own judgements	160	0.35
Confidence	See et al.	2011	4	Confidence in own responses	126	0.39
Confidence	Tost et al.	2012	3	Confidence in own judgement	199	0.34
Confidence	Tost et al.	2012	4	Confidence in own judgement	202	0.46
Confidence	Wojciszke & Struzynska-Kujalowicz	2007	Pilot Study	Better-than-average effect scale	112	0.63
Cooperativeness	Wang et al.	2019	1	Cooperation	104	-0.64
Cooperativeness	Wang et al.	2019	2	Cooperation with innocent third persons	97	-0.73
Creativity	Duguid & Goncalo	2015	4	Number of ideas generated	99	0.86
Creativity	Gervais et al.	2013	1	Generation of creative recipes	38	0.70
Creativity	Gervais et al.	2013	2	Creativity	114	1.06
Creativity	Gervais et al.	2013	3	Creativity	240	0.50
Creativity	Kim et al.	2023	1	Initial idea novelty	153	0.57
Creativity	Kim et al.	2023	2	Initial idea novelty	121	0.43
Creativity	Sligte et al.	2011	1	Remote Associates Task performance	69	0.46
Dehumanization	Gwinn et al.	2013	2	Attribution of not-very-uniquely human traits to others	99	0.44

Dehumanization	Gwinn et al.	2013	1	Attribution of not-very-uniquely human traits to others	44	0.69
Dehumanization	Lammers & Stapel	2011	2	Attribution of animalistic traits to group	47	0.86
Dehumanization	Lammers & Stapel	2011	3	Attribution of mechanistic traits to target person	33	0.90
Desire for power	Lammers et al.	2016	2	Self-reported desire for power	40	-2.36
Desire for power	Lammers et al.	2016	3a	Self-reported desire for power	98	-2.04
Desire for power	Lammers et al.	2016	3b	Self-reported desire for power	71	-0.94
Desire for power	Lammers et al.	2016	3c	Self-reported desire for power	88	-3.19
Desire for power	Van Dijke & Poppe	2006	1	Increase/decrease influence/dependence	22	-1.21
Desire for status	Rucker & Galinsky	2008	1	Willingness to pay for products with a high association with status	41	-0.75
Desire for status	Rucker & Galinsky	2009	2	Preference for status versus quality	69	-0.63
Desire for status	Rucker & Galinsky	2009	3	Emphasis on status versus performance	43	-1.08
Desire for status	Rucker & Galinsky	2009	4	Preference for status over quality	62	-0.91
Desire for status	Rucker & Galinsky	2009	5	Preference for visibly displayed logos	31	-0.87
Desire for status	Choi et al.	2023	1a	Status motive	256	0.52
Desire for status	Dubois et al.	2010	1	Size of a drawn picture of a coin	40	-0.99
Desire for status	Dubois et al.	2010	2	Size of a drawn picture of a coin	120	-1.03
Desire for status	Dubois et al.	2010	3	Size of a drawn picture of a poker chip	90	-1.28
Desire for status	Dubois et al.	2010	4	Estimate size of token (smaller is more valuable)	66	-0.51
Desire for status	Rucker et al.	2014	2b	Willingness to pay for products with a high association with status	48	-0.77
Desire for status	Rucker et al.	2014	2c	Willingness to pay for products with a high association with status	154	-0.63
Empathic accuracy	Nissan et al.	2015	1	Emotion recognition accuracy	105	-0.77
Empathic accuracy	Schmid Mast et al.	2009	2	Accuracy at inferring others' thoughts and feelings	89	0.41
Empathic accuracy	Schmid Mast et al.	2009	3	Emotion recognition accuracy	96	0.52
Empathic accuracy	Uskul et al.	2016	2	Emotion recognition accuracy	115	-0.73
Entitlement	Anicich et al.	2022	3	Psychological entitlement	233	0.43
Entitlement	Sawaoka et al.	2015	Pilot Study	Entitlement to receiving tokens	39	0.91

Fear	Mooijman et al.	2020	3	Fear	182	0.30
Fear	Zhong & Li	2023	2	Fear of repercussions	436	-0.32
Fear	Zhong & Li	2023	3	Fear of repercussions	353	-0.28
Fear	Zhong & Li	2023	1a	Fear of repercussions	145	-0.35
Fear	Zhong & Li	2023	1b	Fear of repercussions	160	-0.72
Forgiveness	Karremans & Smith	2010	2	Inclination to forgive	88	0.46
Giving social support	Hershcovis et al.	2017	1	Providing support	102	-0.55
Goal conflict	Schmid	2018	3	Experienced goal conflict	101	-0.43
Goal conflict	Schmid	2018	4	Experienced goal incompatibility	221	-0.41
Goal pursuit	Burgmer & Englich	2013	2	Goal-relevant detail in drawings of target	47	0.71
Goal pursuit	DeMarree et al.	2012	1	Economic decision-making task influenced by goal prime	92	0.55
Goal pursuit	DeWall et al.	2011	1b	Number of anagrams solved	89	0.35
Goal pursuit	Guinote	2007a	1	Errors in framed-line task	50	0.55
Goal pursuit	Guinote	2007b	3	Number of new attempts to solve problem	79	0.67
Goal pursuit	Jia et al.	2021	2	Perceived restriction on freedom	212	-0.33
Goal pursuit	Min & Kim	2013	3	Recall information	61	1.47
Goal pursuit	Overbeck & Park	2001	1	Free recall of information from previous email	82	1.30
Goal pursuit	Overbeck & Park	2001	2	Free recall of information from previous email	84	0.82
Goal pursuit	Rucker et al.	2014	1a	Discriminating strong from weak candidate	74	0.55
Goal pursuit	Schmid et al.	2018	1	Performance (accuracy) on learning task	75	0.77
Goal pursuit	Scholl et al.	2018	2	Perceived opportunity	115	1.19
Goal pursuit	Slabu & Guinote	2010	1	Response time to goal relevant words during actional phase of goal pursuit (reversed)	18	1.35
Goal pursuit	Slabu & Guinote	2010	2	Response time to goal relevant words during actional phase of goal pursuit (reversed)	50	1.18

Goal pursuit	Whitson et al.	2013	1	Memory of goal-constraining statements (reversed)	48	0.33
Goal pursuit	Whitson et al.	2013	2	Generation of goal-constraining information (reversed)	38	0.58
Goal pursuit	Guinote	2008	1	Behavioral variation depending on day of the week (when there are presumably different goals)	22	1.59
Gratitude	Anicich et al.	2022	2	Expression of gratitude	261	-0.36
Helping behavior	Hershcovis et al.	2017	1	Intention to confront perpetrator	102	0.95
Helping behavior	Hershcovis et al.	2017	Supplement Study 1	Intention to confront perpetrator	142	0.87
Indirect aggression	Zhong & Li	2023	3	Covert competition scale (items are similar to indirect aggression scales)	352	-0.43
Interpersonal orientation	Anicich et al.	2022	3	Interpersonal orientation	233	-0.54
Loneliness	Waytz et al.	2015	6	Loneliness	403	0.19
Loneliness	Waytz et al.	2015	4	Loneliness	435	0.19
Loneliness	Waytz et al.	2015	5	Loneliness	285	0.35
Loneliness	Waytz et al.	2015	2a	Loneliness	56	0.94
Loneliness	Waytz et al.	2015	2c	Loneliness	82	0.59
Loneliness	Waytz et al.	2015	2b	Loneliness	202	0.45
Loss aversion	Inesi	2010	3	Motivation to avoid negative outcome	85	-0.84
Loss aversion	Inesi	2010	2	Motivation to avoid negative outcome	71	-0.52
Lying	Dubois et al.	2015	5	Lying for self-benefit	81	0.84
Lying	Dubois et al.	2015	6	Lying for self-benefit	64	0.66
Lying	Kim et al.	2017	1	Lying about performance	70	0.49
Lying	Kim et al.	2017	2	Lying about performance	54	0.44
Lying	Kim et al.	2017	3	Lying about performance	39	0.64
Lying	Kim et al.	2017	4	Lying about performance	46	0.52
Lying	Li et al.	2022	2	Self-promotional lying	164	-0.31
Lying	Li et al.	2022	3	Self-promotional lying	281	-0.38
Lying	Li et al.	2022	4	Self-promotional lying	536	-0.20
Mental rotation	Nissan et al.	2015	1	Mental rotation	105	0.46
Metastereotyping	Lammers et al.	2008	1	Metastereotype activation	30	-0.84

Metastereotyping	Lammers et al.	2008	2	Metastereotype activation	97	-0.46
Metastereotyping	Lammers et al.	2008	3	Metastereotype activation	65	-0.81
Motivation to apologize	Guilfoyle et al.	2022	2	Motivation to apologize	128	-0.41
Negative affect	Livingston et al.	2022	only study	Number of negative emotion words	403	-0.88
Negative affect	Lücken & Simon	2005	3	Negative affect (reported as positive but reversed based on more negative items)	102	-0.40
Negative affect	Wojciszke & Struzynska-Kujalowicz	2007	Pilot Study	Global affect	112	-1.95
Negotiation initiation	Magee et al.	2007	1a	Likelihood to negotiate	38	0.66
Negotiation initiation	Magee et al.	2007	1b	Likelihood to negotiate	20	0.91
Norm-based (vs consequence-based) moral judgment	Zheng & Guinote	2022	1	Deontological (norm-based) moral judgements	139	0.34
Objectification	Copeland	1994	only study	Getting to know goal-relevant information about other person	48	0.60
Objectification	Gruenfeld et al.	2008	2	Likelihood of selecting candidate who meets goal	140	0.44
Optimism	Anderson & Galinsky	2006	2	Viewing world as less dangerous	36	0.74
Optimism	Fast et al.	2009	2	Optimism about organization’s performance	30	0.82
Optimism	Weick & Guinote	2010	2	Time predictions for task completion	40	1.57
Optimism	Weick & Guinote	2010	3	Time predictions for task completion	64	0.57
Perception of own (vs others') size	Duguid & Goncalo	2012	1	Underestimates of size of objects	46	0.83
Perception of own (vs others') size	Duguid & Goncalo	2012	2	Overestimate of own height	100	0.34
Perception of own (vs others') size	Duguid & Goncalo	2012	3	Height of avatar selected for self	98	0.61
Perception of own (vs others') size	Körner & Schütz	2023	2	Self-report of own height	114	0.55
Perception of own (vs others') size	Yap et al.	2013	2	Underestimates of size of targets	32	0.90
Perception of own (vs others') size	Yap et al.	2013	1	Underestimates of size of targets	85	0.59

Personal space	Case et al.	2015	2	Seating distance	148	0.51
Perspective-taking	Galinsky et al.	2006	1	Self- vs. other-focused drawing of E	57	-0.55
Perspective-taking	Galinsky et al.	2006	2a	Perceptions of sarcasm (with background knowledge)	42	-0.77
Perspective-taking	Galinsky et al.	2006	2b	Perceptions of sincerity (with background knowledge)	51	-0.68
Perspective-taking	Lammers et al.	2008	4	Self-report perspective-taking	139	-0.59
Perspective-taking	Scholl & Sassenberg	2014	2	Self-focused counterfactuals	80	-0.55
Perspective-taking	Scholl & Sassenberg	2014	4	Self-focused thoughts	51	-0.6
Physical appearance self-esteem	Körner & Schütz	2023	2	Body image state scale	114	0.70
Positive affect	Kennedy & Anderson	2017	2	Positive affect	177	1.12
Positive affect	Wojciszke & Struzynska-Kujalowicz	2007	Main Study	Mood	40	1.10
Positive affect	Woltin & Guinote	2015	3	Mood	96	0.70
Preference for desirability (vs feasibility)	Yang & Zhang	2018	5b	Preference for desirability	176	0.37
Prejudice	Guinote et al.	2010	1	Response time for positive and negative word associations for black and white faces	49	0.85
Prejudice	Guinote et al.	2010	3	Score on IAT	76	0.50
Prejudice	Guinote et al.	2010	2	Proportion of pleasant responses after seeing each White vs. Black prime	44	0.66
Response conflict executive function	Egan & Hirt	2015	2	Latency in Stroop responses	85	0.56
Response conflict executive function	Schmid et al.	2015	1	Flanker test performance	83	0.47
Response conflict executive function	Schmid et al.	2015	2	Flanker test performance	96	0.52
Response conflict executive function	Smith et al.	2008	2	Error rate on incongruent Stroop interference trials	48	0.67
Response conflict executive function	Smith et al.	2008	4	Error rate on incongruent Stroop interference trials	114	0.79

Response conflict executive function	Willis et al.	2011	2	ANTI (adaptation of Flanker) test performance	84	0.45
Revenge	Struthers et al.	2019	2	Decision to seek revenge	181	0.31
Reward sensitivity	Smith & Bargh	2008	1	BAS scale	68	0.03
Risk-taking	Anderson & Galinsky	2006	3	Preference for riskier vs. neutral plan	57	0.63
Risk-taking	Jordan et al.	2011	1	Preference for risk in Asian disease paradigm	193	0.47
Self-esteem	Wojciszke & Struzynska-Kujalowicz	2007	Main Study	Self-esteem	40	0.70
Selfishness	Kopelman	2009	only study	Egocentric fairness decision	204	0.61
Selfishness	Liu et al.	2020	Chinese sample	Taking extra payment for self	100	0.85
Selfishness	Rucker et al.	2011	1	Amount bid on object for self vs. others	120	0.94
Selfishness	Rucker et al.	2011	2	Number of chocolates purchased for oneself	53	0.94
Selfishness	Rucker et al.	2011	3	Amount spent on oneself	160	1.44
Selfishness	Rucker et al.	2011	4	Amount spent on oneself	96	0.87
Selfishness	Rucker et al.	2011	5	Number of chocolates purchased for self	80	0.70
Selfishness	Rus et al.	2012	1	Allocation of points to self	40	0.81
Selfishness	Rus et al.	2012	2	Intent to work with team on weekend (reversed)	43	0.97
Selfishness	Zhong & Li	2023	2	Taking extra payment for self	436	-0.40
Sense of personal control	Cai & Wu	2017	2	Perceived control	136	1.01
Sense of personal control	Fast et al.	2009	1	Preference for self vs. other to roll die of which they predicted the outcome	25	1.21
Sense of personal control	Fast et al.	2009	2	Perceived control	30	2.43
Stereotyping	Overbeck & Park	2001	1	Judgment of targets’ similarity	82	-0.87
Stereotyping	Schmid & Amodio	2017	1	Stereotyping task	83	0.55
Subjective well-being	Kifer et al.	2013	2a	Subjective well-being	252	0.42
Susceptibility to influence	Anderson & Berdahl	2002	2	Partner influence	130	-0.48

Susceptibility to influence	Anderson & Thompson	2004	1	Likelihood of reaching integrative agreement following counterpart's positive affect	92	-0.79
Susceptibility to influence	Galinsky et al.	2008	2	Influence of environmental stimuli in drawing task	75	-0.55
Susceptibility to influence	Galinsky et al.	2008	3	Influence of others' opinions	21	-0.97
Susceptibility to influence	Lammers & Burgmer	2017	internal meta-analysis	Influence of anchors	2207	0.14
Susceptibility to influence	Min & Kim	2013	3	Attitude toward advertised product	57	-1.91
Susceptibility to influence	See et al.	2011	3	Weight placed on advice from others	160	-0.46
Susceptibility to influence	See et al.	2011	4	Weight placed on advice from others	126	-0.54
Susceptibility to influence	Tost et al.	2012	1	Use of advice	71	-1.15
Susceptibility to influence	Tost et al.	2012	2	Receptivity to advice	132	-1.47
Susceptibility to influence	Tost et al.	2012	3	Receptivity to advice from experts	199	-0.91
Susceptibility to influence	Van Kleef et al.	2006	1	Size of concession made	80	-1.15
Susceptibility to influence	Van Kleef et al.	2006	3	Size of offer made following counterpart's emotion expression	65	-0.97
Susceptibility to influence	Van Kleef et al.	2006	4	Likelihood to concede following counterpart's emotion expression	93	-0.70
Susceptibility to influence	Wojciszke & Struzynska-Kujalowicz	2007	Main Study	Partner influence	40	-1.17
Temporal discounting	Zhang & Smith	2018	meta-analysis	Temporal discounting	1772	-0.11
Trust in others	du Plessis et al.	2023	internal meta-analysis	Trust in others	6931	0.26
Trust in others	Inesi et al.	2012	2	Attribution of selfish motives to others (reversed)	98	-0.77

Trust in others	Inesi et al.	2012	3	Attributions of instrumentality to others (reversed)	66	-1.15
Trust in others	Inesi et al.	2012	5	Trust in others	131	-0.41
Trust in others	Schaerer et al.	2021	1	Paranoia (reversed)	300	0.51
Trust in others	Schilke et al.	2015	1	Pre-negotiation trust in counterpart	401	-0.35
Trust in others	Schilke et al.	2015	2	Trust in others	348	-0.29
Trust in others	Schilke et al.	2015	3	Trust in others	452	-0.41
Trust in others	Schilke et al.	2015	4	Trust in others	401	-0.21
Updating executive function: n-back task	Smith et al.	2008	1	Errors on a 2-back test (updating performance)	95	0.46
Visual-spatial working memory	Hadar et al.	2020	1	Recognition of test stimuli from previously presented array	149	0.04
Visual-spatial working memory	Hadar et al.	2020	2	Recognition of test stimuli from previously presented array	100	0.45
Visual-spatial working memory	Hadar et al.	2020	3	Recognition of test stimuli from previously presented array	100	0.63
Voice	Wan & Li	2021	1	Customer voice behavior	62	0.25
Voice	Wan & Li	2021	2	Customer voice behavior	66	2.68
Voice	Wan & Li	2021	3	Voice behavior intention	146	0.86
**Included in P-curve only (excluded from other analyses)**						
Depth of processing	Min & Kim	2013	1	Time looking at advertisement	68	-1.98
Depth of processing	Min & Kim	2013	2	Time looking at advertisement	76	-3.69
Disgust	Mooijman et al.	2020	3	Disgust	182	0.30
Inequity aversion	Hou & Meng	2022	only study	Rejection rate of unfair offers	48	0.50
Inequity aversion	Wang et al.	2019	1	Perceived fairness of outcome distribution	104	0.55
Moral condemnation	Kennedy & Anderson	2017	2	Strength of dissent	177	-0.59
Moral condemnation	Mooijman et al.	2020	4	Moral condemnation	137	-0.41
Moral hypocrisy	Lammers et al.	2010	4	Own behavior vs judgment of others stealing bike	74	0.94
Moral hypocrisy	Lammers et al.	2010	1	Number of lottery tickets claimed for self plus moral judgment of others	61	0.74

Moral hypocrisy	Lammers et al.	2010	3	Own behavior vs judgments of others dodging taxes	72	0.74
Negotiation performance	Pinkley et al.	1995	1	Points claimed in negotiation	231	0.23
Negotiation performance	Schaerer et al.	2015	1a	Magnitude of first offer	288	0.76
Negotiation performance	Schaerer et al.	2015	1b	Magnitude of	289	1.91
Negotiation performance	Schaerer et al.	2015	3	Final negotiated agreement amount	95	0.93
Perceived responsibility	Scholl et al.	2018	2	Perceived responsibility	112	1.28
Preference for choice	Inesi et al.	2011	1a	Preference for choice	40	-0.71
Preference for choice	Inesi et al.	2011	1b	Willingness to drive further for choice	40	-0.66
Role identification	Joshi & Fast	2013	2	Role identification	145	0.87
Source attribution accuracy	Overbeck & Park	2001	1	Source attribution accuracy	82	1.49
Source attribution accuracy	Overbeck & Park	2001	2	Source attribution accuracy	84	0.91

Table SI3. Excluded power studies with reason for exclusion.

Source	Study	Exclusion Rule
M. Alonso-Ferres, I. Valor-Segura, F. Expósito, Elucidating the effect of perceived power on destructive responses during romantic conflicts. *Span. J. Psychol*. **24**, (2021).	all	Romantic relationship sample
C. Anderson, J.L. Berdahl, The experience of power: Examining the effects of power on approach and inhibition tendencies. *J. Pers. Soc. Psychol.* **83**, 1362–1377 (2002).	1	Power not manipulated
C. Anderson, A.D. Galinsky, Power, optimism, and risk-taking. *Eur. J. Soc. Psychol.* **36**, 511–536 (2006).	1	Power not manipulated
C. Anderson, A.D. Galinsky, Power, optimism, and risk-taking. *Eur. J. Soc. Psychol.* **36**, 511–536 (2006).	4	Sexual behavior
C. Anderson, A.D. Galinsky, Power, optimism, and risk-taking. *Eur. J. Soc. Psychol.* **36**, 511–536 (2006).	5	Non-significant result
C. Anderson, L.L. Thompson, Affect from the top down: How powerful individuals' positive affect shapes negotiations. *Organ. Behav. Hum. Decis.* **95**, 125–139 (2004).	2	Inadequate statistics
E.M. Anicich, A.J. Lee, S. Liu, Thanks, but no thanks: Unpacking the relationship between relative power and gratitude. *Pers. Soc. Psychol. Bull.* **48**, 1005-1023 (2022).	1, 4	Power not manipulated
A.J. Barends, R.E. de Vries, M. van Vugt, Power influences the expression of honesty-humility: The power-exploitation affordances hypothesis. *J. Res. Pers.* **82**, (2019).	all	Missing high- or low-power condition
J.A. Bargh, P. Raymond, J.B. Pryor, F. Strack, Attractiveness of the underling: An automatic power → sex association and its consequences for sexual harassment and aggression. *J. Pers. Soc. Psychol.* **68**, 768–781 (1995).	all	Sexual behavior
J.L. Berdahl, P. Martorana, Effects of power on emotion and expression during a controversial group discussion. *Eur. J. Soc. Psychol.* **36**, 497–509 (2006).	1	Group/team design
S.L. Blader, Y.R. Chen, Differentiating the effects of status and power: A justice perspective. *J. Pers. Soc. Psychol.* **102**, 994–1014 (2012).	1-3	Missing high- or low-power condition

S.L. Blader, Y.R. Chen, Differentiating the effects of status and power: A justice perspective. *J. Pers. Soc. Psychol.* **102**, 994–1014 (2012).	4	Non-significant result
S.L. Blader, A. Shirako, Y.R. Chen, Looking out from the top: Differential effects of status and power on perspective taking. *Pers. Soc. Psychol. Bull.* **42**, 723-737 (2016).	1, 2, 5	Missing high- or low-power condition
S.L. Blader, A. Shirako, Y.R. Chen, Looking out from the top: Differential effects of status and power on perspective taking. *Pers. Soc. Psychol. Bull.* **42**, 723-737 (2016).	3, 4	Non-significant result
M.A.S. Boksem, R. Smolders, D. de Cremer, Social power and approach-related neural activity. *Soc. Cogn. Affect. Neurosci.* **7**, 516–520 (2012).	only study	Neuroimaging
P. Briñol, R.E. Petty, C. Valle, D.D. Rucker, A. Becerra, The effects of message recipients' power before and after persuasion: A self-validation analysis. *J. Pers. Soc. Psychol.* **93**, 1040–1053 (2007).	2, 3	No main/simple effect
S. Brion, R. Mo, R.B. Lount Jr, Dynamic influences of power on trust: Changes in power affect trust in others. *J. Trust Res.* **9**, 6-27 (2019).	only study	Power not manipulated
P. Burgmer, B. Englich, Bullseye!. *Soc. Psychol. Pers. Sci.* **4**, 224–232 (2013).	1	Missing high- or low-power condition
W. Cai, A. Guinote, S. Wu, Revisiting the powerful-not-lonely effect across cultures: The mediating role of self-construal and social support. *Curr. Psychol.* **42**, 8824–8832 (2023).	all	Power not manipulated
W. Cai, S. Wu, Powerful people feel less fear of negative evaluation. *Soc. Psychol*. **48**, 85-91 (2017).	1	Power not manipulated
D.R. Carney, A.J. Cuddy, A.J. Yap, Power posing: Brief nonverbal displays affect neuroendocrine levels and risk tolerance. *Psychol. Sci.* **21**, 1363–1368 (2010).	only study	Power posing manipulation
E.W. Carr, P. Winkielman, C. Oveis, Transforming the mirror: power fundamentally changes facial responding to emotional expressions. *J. Exp. Soc. Psychol.* **143**, 997 (2014).	only study	No main/simple effect
S. Chen, C.A. Langner, R. Mendoza-Denton, When dispositional and role power fit: Implications for self-expression and self–other congruence. *J. Pers. Soc. Psychol.* **96**, 710–727 (2009).	all	No main/simple effect

S. Chen, A.Y. Lee-Chai, J.A. Bargh, Relationship orientation as a moderator of the effects of social power. *J. Pers. Soc. Psychol.* **80**, 173–187 (2001).	all	No main/simple effect
S. Chen, O. Ybarra, A.K. Kiefer, Power and impression formation: The effects of power on the desire for morality and competence information. *Soc. Cogn.* **22**, 391–421 (2004).	all	Missing high- or low-power condition
C. Civile, S.S. Obhi, Power, objectification, and recognition of sexualized women and men. *Psychol. Women. Q.* **40**, 199–212 (2016).	1	All women sample
C. Civile, S.S. Obhi, Power, objectification, and recognition of sexualized women and men. *Psychol. Women. Q.* **40**, 199–212 (2016).	2	No main/simple effect
S. Côté, M.W. Kraus, B.H. Cheng, C. Oveis, I. van der Löwe, H. Lian, D. Keltner, Social power facilitates the effect of prosocial orientation on empathic accuracy. *J. Pers. Soc. Psychol.* **101**, 217–232 (2011).	1, 3	Power not manipulated
S. Côté, M.W. Kraus, B.H. Cheng, C. Oveis, I. van der Löwe, H. Lian, D. Keltner, Social power facilitates the effect of prosocial orientation on empathic accuracy. *J. Pers. Soc. Psychol.* **101**, 217–232 (2011).	2	No main/simple effect
K.A. DeCelles, D.S. DeRue, J.D. Margolis, T.L. Ceranic, Does power corrupt or enable? When and why power facilitates self-interested behavior. *J. Appl. Psychol.* **97**, 681–689 (2012).	1	Power not manipulated
K.A. DeCelles, D.S. DeRue, J.D. Margolis, T.L. Ceranic, Does power corrupt or enable? When and why power facilitates self-interested behavior. *J. Appl. Psychol.* **97**, 681–689 (2012).	2	Missing high- or low-power condition
D. de Cremer, E. van Dijk, When and why leaders put themselves first: Leader behavior in resource allocations as a function of feeling entitled. *Eur. J. Soc. Psychol.* **35**, 553–563 (2005).	all	Power not manipulated
C.K. de Dreu, G.A. Van Kleef, The influence of power on the information search, impression formation, and demands in negotiation. *J. Exp. Soc. Psychol.* **40**, 303–319 (2004).	1, 2	No main/simple effect
K.G. DeMarree, L. Loersch, P. Brinol, R.E. Petty, B.K. Payne, D.D. Rucker, From primed construct to motivated behavior: Validation	2-3	Power not manipulated

processes in goal pursuit. *Pers. Soc. Psychol. Bull.* **38**, 1659–1970 (2012).		
C.N. DeWall, R.F. Baumeister, N.L. Mead, K.D. Vohs, How leaders self-regulate their task performance: Evidence that power promotes diligence, depletion, and disdain. *J. Pers. Soc. Psychol.* **100**, 47–65 (2011).	3, 4	Missing high- or low-power condition
C.N. DeWall, R.F. Baumeister, N.L. Mead, K.D. Vohs, How leaders self-regulate their task performance: Evidence that power promotes diligence, depletion, and disdain. *J. Pers. Soc. Psychol.* **100**, 47–65 (2011).	5	No main/simple effect
J.F. Dovidio, S.L. Ellyson, C.F. Keating, K. Heltman, C.E. Brown, The relationship of social power to visual displays of dominance between men and women. *J. Pers. Soc. Psychol.* **54**, 233–242 (1988).	all	No main/simple effect
M.M. Duguid, J.A. Goncalo, Squeezed in the middle: The middle status trade creativity for focus. *J. Pers. Soc. Psychol.* **109**, 589–603 (2015).	5	Leadership/status conflated with power
D. Dubois, D.D. Rucker, A.D. Galinsky, Super size me: Product size as a signal of status. *J. Consum. Res.* **38**, 1047–1062 (2012).	all	Editor published expression of concern about the research
D. Dubois, D.D. Rucker, A.D. Galinsky, Social class, power, and selfishness: When and why upper and lower class individuals behave unethically. *J. Pers. Soc. Psychol.* 108, 436-449 (2015).	1-4	Power not manipulated
D. Dubois, D.D. Rucker, A.D. Galinsky, Dynamics of communicator and audience power: The persuasiveness of competence versus warmth. *J. Consum. Res.* **43**, 68–85 (2016).	all	Editor published expression of concern about the research
A.A. Eaton, P.S. Visser, J.A. Krosnick, S. Anand, Social power and attitude strength over the life course. *Pers. Soc. Psychol. Bull.* **35**, 1646–1660 (2009).	1-3, 5	Power not manipulated
A.A. Eaton, P.S. Visser, J.A. Krosnick, S. Anand, Social power and attitude strength over the life course. *Pers. Soc. Psychol. Bull.* **35**, 1646–1660 (2009).	4	Missing high- or low-power condition
C.R. Ebersole, O.E. Atherton, A.L. Belanger, H.M. Skulborstad, J.M. Allen, J.B. Banks, … B.A. Nosek, Many Labs 3: Evaluating participant	4	Non-significant result

pool quality across the academic semester via replication. *J. Exp. Soc. Psychol.* **67**, 68–82 (2016).		
P.M. Egan, E.R. Hirt, Flipping the switch: Power, social dominance, and expectancies of mental energy change. *Pers. Soc. Psychol. Bull.* **41**, 336-350 (2015).	1	Power not manipulated
P.M. Egan, E.R. Hirt, Flipping the switch: Power, social dominance, and expectancies of mental energy change. *Pers. Soc. Psychol. Bull.* **41**, 336-350 (2015).	3	No main/simple effect
R.M. Emerson, Power-dependence relations: Two experiments. *Sociometry.* **27**, 282–298 (1964).	1	Power not manipulated
R.M. Emerson, Power-dependence relations: Two experiments. *Sociometry.* **27**, 282–298 (1964).	2	Group/team design
N.J. Fast, S. Chen, When the boss feels inadequate: Power, incompetence, and aggression. *Psychol. Sci.* **20**, 1406–1413 (2009).	1, 4	Power not manipulated
N.J. Fast, S. Chen, When the boss feels inadequate: Power, incompetence, and aggression. *Psychol. Sci.* **20**, 1406–1413 (2009).	2, 3	Missing high- or low-power condition
N.J. Fast, D.H Gruenfeld, N. Sivanathan, A.D. Galinsky, Illusory Control. *Psychol. Sci.* **20**, 502–508 (2009).	3	Missing high- or low-power condition
N.J. Fast, N. Halevy, A.D. Galinsky, The destructive nature of power without status. *J. Exp. Soc. Psychol.* **48**, 391–394 (2012).	only study	Dependent measure includes too many disparate behaviors to categorize
N.J. Fast, N. Sivanathan, N.D. Mayer, A.D. Galinsky, Power and overconfident decision-making. *Organ. Behav. Hum. Decis. Proc.* **117**, 249–260 (2012).	4	Power not manipulated
N.J. Fast, N. Sivanathan, N.D. Mayer, A.D. Galinsky, Power and overconfident decision-making. *Organ. Behav. Hum. Decis. Proc.* **117**, 249–260 (2012).	5	No main/simple effect
J. Fischer, P. Fischer, B. Englich, N. Aydin, D. Frey, Empower my decisions: The effects of power gestures on confirmatory information processing. *J. Exp. Soc. Psychol.* **47**, 1146–1154 (2011).	all	Power posing manipulation
T.A. Foulk, I.E. De Pater, M. Schaerer, C. du Plessis, R. Lee, A. Erez, It’s lonely at the bottom (too): The effects of experienced powerlessness on social closeness and disengagement. *Pers. Psychol.* **73**, 363–394 (2020).	all	Missing high- or low-power condition

A.D. Galinsky, J.C. Magee, M.E. Inesi, and D.H Gruenfeld, Power and perspectives not taken. *Psychol. Sci.* **17**, 1068–1074 (2006).	3	Missing high- or low-power condition
A.D. Galinsky, J.C. Magee, D.H Gruenfeld, J.A. Whitson, K.A. Liljenquist, Power reduces the press of the situation: Implications for creativity, conformity, and dissonance. *J. Pers. Soc. Psychol.* **95**, 1450–1466 (2008).	1, 4	Missing high- or low-power condition
A.D. Galinsky, J.C. Magee, D.H Gruenfeld, J.A. Whitson, K.A. Liljenquist, Power reduces the press of the situation: Implications for creativity, conformity, and dissonance. *J. Pers. Soc. Psychol.* **95**, 1450–1466 (2008).	5	Non-significant result
J.C. Georgesen, M.J. Harris, The balance of power: Interpersonal consequences of differential power and expectancies. *Pers. Soc. Psychol. Bull.* **26**, 1239–1257 (2000).	only study	No main/simple effect
P.H. Goh, P. Stoeckli, D. Schoebi, H. Annen, The source of power matters: Positional power as a better predictor of sexual interest perceptions than dispositional power among men within a military context. *Arch. Sex. Behav.* **51**, 1531–1539 (2022).	all	All men sample
G.C. Gonzaga, D. Keltner, D. Ward, Power in mixed-sex stranger interactions. *Cogn. Emot.* **22**, 1555–1568 (2008).	only study	Inadequate statistics
S.A. Goodwin, A. Gubin, S.T. Fiske, V.Y. Yzerbyt, Power can bias impression processes: Stereotyping subordinates by default and by design. *Group Process. Intergr. Relat.* **3**, 227–256 (2000).	1	Missing high- or low-power condition
S.A. Goodwin, A. Gubin, S.T. Fiske, V.Y. Yzerbyt, Power can bias impression processes: Stereotyping subordinates by default and by design. *Group Process. Intergr. Relat.* **3**, 227–256 (2000).	2	Power not manipulated
S.A. Goodwin, A. Gubin, S.T. Fiske, V.Y. Yzerbyt, Power can bias impression processes: Stereotyping subordinates by default and by design. *Group Process. Intergr. Relat.* **3**, 227–256 (2000).	3	Inadequate statistics
S.A. Goodwin, A. Gubin, S.T. Fiske, V.Y. Yzerbyt, Power can bias impression processes: Stereotyping subordinates by default and by design. *Group Process. Intergr. Relat.* **3**, 227–256 (2000).	4	Non-significant result
S.A. Goodwin, D. Operario, S.T. Fiske, Situational power and interpersonal dominance facilitate bias and inequality. *J. Soc. Issues.* **54**, 677–698 (1998).	all	Inadequate statistics

D.H Gruenfeld, M.E. Inesi, J.C. Magee, A.D. Galinsky, Power and the objectification of social targets. *J. Pers. Soc. Psychol.* **95**, 111–127 (2008).	1a, 4, 5	Missing high- or low-power condition
D.H Gruenfeld, M.E. Inesi, J.C. Magee, A.D. Galinsky, Power and the objectification of social targets. *J. Pers. Soc. Psychol.* **95**, 111–127 (2008).	1b	Non-significant result
D.H Gruenfeld, M.E. Inesi, J.C. Magee, A.D. Galinsky, Power and the objectification of social targets. *J. Pers. Soc. Psychol.* **95**, 111–127 (2008).	3	Sexual behavior
J.R. Guilfoyle, C.W. Struthers, E. van Monsjou, A. Shoikhedbrod, N. Eghbali, M. Kermani, Sorry, not sorry: The effect of social power on transgressors' apology and nonapology. *J. Exp. Psychol.: Appl.* **28**, 883-897 (2022).	1, 3	Power not manipulated
J.R. Guilfoyle, C.W. Struthers, E. van Monsjou, A. Shoikhedbrod, N. Eghbali, M. Kermani, Sorry, not sorry: The effect of social power on transgressors' apology and nonapology. *J. Exp. Psychol.: Appl.* **28**, 883-897 (2022).	4	No main/simple effect
A. Guinote, Power affects basic cognition: Increased attentional inhibition and flexibility. *J. Exp. Soc. Psychol.* **43**, 685–697 (2007).	2, 3	Non-significant result
A. Guinote, Power and affordances: When the situation has more power over powerful than powerless individuals. *J. Pers. Soc. Psychol.* **95**, 237–252 (2008).	2, 4, 5	No main/simple effect
A. Guinote, Power and affordances: When the situation has more power over powerful than powerless individuals. *J. Pers. Soc. Psychol.* **95**, 237–252 (2008).	3	Power not manipulated
A. Guinote, Power and affordances: When the situation has more power over powerful than powerless individuals. *J. Pers. Soc. Psychol.* **95**, 237–252 (2008).	6	Non-significant result
A. Guinote, Power increases reliance on bodily information. *Soc. Cogn.* **28**, 110–121 (2010).	all	No main/simple effect
A. Guinote, C.M. Judd, M. Brauer, Effects of power on perceived and objective group variability: Evidence that more powerful groups are more variable. *J. Pers. Soc. Psychol.* **82**, 708–721 (2002).	1	Group/team design

A. Guinote, C.M. Judd, M. Brauer, Effects of power on perceived and objective group variability: Evidence that more powerful groups are more variable. *J. Pers. Soc. Psychol.* **82**, 708–721 (2002).	2	Participants' power not manipulated
A. Guinote, A. Phillips, Power can increase stereotyping. *Soc. Psychol.* **41**, 3–9 (2010).	only study	Power not manipulated
A. Guinote, M. Weick, A. Cai, Does power magnify the expression of dispositions? *Psychol. Sci.* **94**, 956–970 (2012).	all	No main/simple effect
T. Harada, D.J. Bridge, J.Y. Chiao, Dynamic social power modulates neural basis of math calculation. *Front. Hum. Neurosci.* **6**, 350 (2013).	only study	All women sample
M.A. Hecht, M. LaFrance, License or obligation to smile: The effect of power and sex on amount and type of smiling. *Pers. Soc. Psychol. Bull.* **24**, 1332–1342 (1998).	only study	Inadequate statistics
M.S. Hershcovis, L. Neville, T.C. Reich, A.M. Christie, L.M. Cortina, J.V. Shan, Witnessing wrongdoing: The effects of observer power on incivility intervention in the workplace. *Organ. Behav. Hum. Decis. Proc.* **142**, 45-57 (2017).	2, Supplement Study 2	Power not manipulated
M.S. Hershcovis, L. Neville, T.C. Reich, A.M. Christie, L.M. Cortina, J.V. Shan, Witnessing wrongdoing: The effects of observer power on incivility intervention in the workplace. *Organ. Behav. Hum. Decis.* **142**, 45-57 (2017).	3	Leadership/status conflated with power
J.A.D. Hildreth, C. Anderson, Failure at the top: how power undermines collaborative performance. *J. Pers. Soc. Psychol*. **110**, 261–286 (2016).	all	Group/team design
N. Hoogervorst, D. De Cremer, M. van Dijke, D.M. Mayer, When do leaders sacrifice? The effects of sense of power and belongingness on leader self-sacrifice. *Leadersh. Q.* **23**, 883–896 (2012).	1, 2	Power not manipulated
N. Hoogervorst, D. De Cremer, M. van Dijke, D.M. Mayer, When do leaders sacrifice? The effects of sense of power and belongingness on leader self-sacrifice. *Leadersh. Q.* **23**, 883–896 (2012).	3	No main/simple effect
E.S. Howard, W.L. Gardner, L. Thompson, The role of the self-concept and the social context in determining the behavior of power holders: Self-construal in intergroup versus dyadic dispute resolution negotiations. *J. Pers. Soc. Psychol.* **93**, 614–631 (2007).	1, 2	No main/simple effect

E.S. Howard, W.L. Gardner, L. Thompson, The role of the self-concept and the social context in determining the behavior of power holders: Self-construal in intergroup versus dyadic dispute resolution negotiations. *J. Pers. Soc. Psychol.* **93**, 614–631 (2007).	3	Missing high- or low-power condition
M. Hu, D.D. Rucker, A.D. Galinsky, From the immoral to the incorruptible: How prescriptive expectations turn the powerful into paragons of virtue. *Pers. Soc. Psychol. Bull.* **42**, 826–837 (2016).	all	No main/simple effect
L. Huang, A.D. Galinsky, D.H Gruenfeld, L.E. Guillory, Powerful postures versus powerful roles: Which is the proximate correlate of thought and behavior? *Psychol. Sci.* **22**, 95–102 (2011).	all	No main/simple effect
S. Hyun, X. Ku, How does power affect happiness and mental illness? The mediating role of proactive coping. *Cogent Psychol*., **7,** (2020).	1	Power not manipulated
M.E. Inesi, Power and loss aversion. *Organ. Behav. Hum. Decis. Proc.* **112**, 58–69 (2010).	1, 4	Missing high- or low-power condition
M.E. Inesi, S. Botti, D. Dubois, D.D. Rucker, A.D. Galinsky, Power and choice: Their dynamic interplay in quenching the thirst for personal control. *Psychol. Sci.* **22**, 1042–1048 (2011).	2a-b	Power not manipulated
M.E. Inesi, S. Botti, D. Dubois, D.D. Rucker, A.D. Galinsky, Power and choice: Their dynamic interplay in quenching the thirst for personal control. *Psychol. Sci.* **22**, 1042–1048 (2011).	3a	No main/simple effect
M.E. Inesi, S. Botti, D. Dubois, D.D. Rucker, A.D. Galinsky, Power and choice: Their dynamic interplay in quenching the thirst for personal control. *Psychol. Sci.* **22**, 1042–1048 (2011).	3b	Missing high- or low-power condition
M.E. Inesi, D.H Gruenfeld, A.D. Galinsky, How power corrupts relationships: Cynical attributions for others' generous acts. *J. Exp. Soc. Psychol.* **48**, 795–803 (2012).	1	Missing high- or low-power condition
M.E. Inesi, D.H Gruenfeld, A.D. Galinsky, How power corrupts relationships: Cynical attributions for others' generous acts. *J. Exp. Soc. Psychol.* **48**, 795–803 (2012).	4	Romantic relationship sample
Y. Jia, R.S. Wyer Jr, H. Shen, "Will you?" versus "can you?": Verbal framing moderates the effect of feelings of power on consumers' reactions to waiting. *J. Exp Psychol: Appl.* **27**, 213-227 (2021).	1, 4, 5	Non-significant result

Y. Jia, R.S. Wyer Jr, H. Shen, “Will you?” versus “can you?”: Verbal framing moderates the effect of feelings of power on consumers’ reactions to waiting. *J. Exp Psychol: Appl.* **27**, 213-227 (2021).	3	Power not manipulated
J. Jordan, N. Sivanathan, A.D. Galinsky, Something to lose and nothing to gain: The role of stress in the interactive effect of power and stability on risk taking. *Adm. Sci. Q.***56**, 530–558 (2011).	1-3, 5	No main/simple effect
J. Jordan, N. Sivanathan, A.D. Galinsky, Something to lose and nothing to gain: The role of stress in the interactive effect of power and stability on risk taking. *Adm. Sci. Q.* **56**, 530–558 (2011).	4	Power not manipulated
P.D. Joshi, N.J. Fast, I am my (high-power) role: Power and role identification. *Pers. Soc. Psychol. Bull.* **39**, 898–910 (2013).	Pilot Study	Power not manipulated
P.D. Joshi, N.J. Fast, I am my (high-power) role: Power and role identification. *Pers. Soc. Psychol. Bull.* **39**, 898–910 (2013).	1, 3	No main/simple effect
P.D. Joshi, C.J. Wakslak, G. Appel, L. Huang, Gender differences in communicative abstraction. *J. Pers. Soc. Psychol.* **118**, 417-435 (2020).	1-4	Power not manipulated
E. Kacewicz, J.W. Pennebaker, M. Davis, M. Jeon, A.C. Graesser, Pronoun use reflects standings in social hierarchies. *J. Lang. Soc. Psychol.* **33**, 125–143 (2014).	all	Power not manipulated
S.K. Kang, A.D. Galinsky, L.J. Kray, A. Shirako, Power affects performance when the pressure is on: evidence for low-power threat and high-power lift. *Pers. Soc. Psychol. Bull*. **41**, 726–735 (2015).	all	No main/simple effect
J.C. Karremans, P.K. Smith, Having the power to forgive: When the experience of power increases interpersonal forgiveness. *Pers. Soc. Psychol. Bull.* **36**, 1010–1023 (2010).	1, 3	Participants' power not manipulated
D. Keltner, R.J. Robinson, Defending the status quo: Power and bias in social conflict. *J. Pers. Soc. Psychol.* **23**, 1066–1077 (1997).	only study	Power not manipulated
J.A. Kennedy, C. Anderson, Hierarchical rank and principled dissent: How holding higher rank suppresses objection to unethical practices. *Organ. Behav. Hum. Decis. Proc.* **139**, 30-49 (2017).	1	Power not manipulated
J.A. Kennedy, C. Anderson, Hierarchical rank and principled dissent: How holding higher rank suppresses objection to unethical practices. *Organ. Behav. Hum. Decis. Proc.* **139**, 30-49 (2017).	3	Leadership/status conflated with power

Y. Kifer, D. Heller, W.Q.E. Perunovic, A.D. Galinsky, The good life of the powerful: The experience of power and authenticity enhances subjective well-being. *Psychol. Sci.* **24**, 280–288 (2013).	1, 2b	Power not manipulated
G.J. Kilduff, A.D. Galinsky, From the ephemeral to the enduring: Approach-oriented mindsets lead to greater status. *J. Pers. Soc. Psychol.* **105**, 816–831 (2013).	1, 3	Power not manipulated
G.J. Kilduff, A.D. Galinsky, From the ephemeral to the enduring: Approach-oriented mindsets lead to greater status. *J. Pers. Soc. Psychol.* **105**, 816–831 (2013).	2	Group/team design
D. Kipnis, Does power corrupt? *J. Pers. Soc. Psychol.* **24**, 33–41 (1972).	only study	Missing high- or low-power condition
D. Kipnis, J. Castell, M. Gergen, D. Mauch, Metamorphic effects of power. *J. Appl. Psychol.* **61**, 127–135 (1976).	all	Power not manipulated
S.J. Ko, M.S. Sadler, A.D. Galinsky, The sound of power: Conveying and detecting hierarchical rank through voice. *Psychol. Sci.* **26**, 3–14 (2015).	1	Leadership/status conflated with power
S.J. Ko, M.S. Sadler, A.D. Galinsky, The sound of power: Conveying and detecting hierarchical rank through voice. *Psychol. Sci.* **26**, 3–14 (2015).	2	Participants' power not manipulated
R. Körner, A. Schütz, Power, self-esteem, and body image. *Soc. Psychol.*, **54**, 136-150 (2023).	1	Power not manipulated
M.W. Kraus, S. Chen, D. Keltner, The power to be me: Power elevates self-concept consistency and authenticity. *J. Exp. Soc. Psychol*. **47**, 974–980 (2011).	1	Power not manipulated
M.W. Kraus, S. Chen, D. Keltner, The power to be me: Power elevates self-concept consistency and authenticity. *J. Exp. Soc. Psychol*. **47**, 974–980 (2011).	2	Inadequate statistics
J.W. Kunstman, J.K. Maner, Sexual overperception: Power, mating motives, and biases in social judgment. *J. Pers. Soc. Psychol.* **100**, 282–294 (2011).	all	Sexual behavior
J. Lammers, P. Burgmer, Power increases the self-serving bias in the attribution of collective successes and failures. *Eur. J. Soc. Psychol.* **49**, 1087–1095 (2019).	1, 3	Power not manipulated

J. Lammers, P. Burgmer, Power increases the self-serving bias in the attribution of collective successes and failures. *Eur. J. Soc. Psychol.* **49**, 1087–1095 (2019).	2	Non-significant result
J. Lammers, D. Dubois, D.D. Rucker, A.D. Galinsky, Power gets the job: Priming power improves interview outcomes. *J. Exp. Soc. Psychol.* **49**, 776–779 (2013).	1	Non-significant result
J. Lammers, D. Dubois, D.D. Rucker, A.D. Galinsky, Power gets the job: Priming power improves interview outcomes. *J. Exp. Soc. Psychol.* **49**, 776–779 (2013).	2	Observer assessment measure
J. Lammers, D. Dubois, D.D. Rucker, A.D. Galinsky, Ease of retrieval moderates the effects of power: Implications for the replicability of power recall effects. *Soc. Cogn*. **35**, 1-17 (2017).	1	Inadequate statistics
J. Lammers, D. Dubois, D.D. Rucker, A.D. Galinsky, Ease of retrieval moderates the effects of power: Implications for the replicability of power recall effects. *Soc. Cogn*. **35**, 1-17 (2017).	2, 3	Non-significant result
J. Lammers, A.D. Galinsky, E.H. Gordijn, S. Otten, Illegitimacy moderates the effects of power on approach. *Psychol. Sci.* **19**, 558–564 (2008).	all	No main/simple effect
J. Lammers, A.D. Galinsky, E.H. Gordijn, S. Otten, Power increases social distance. *Soc. Psychol. Pers. Sci.* **3**, 282–290 (2012).	all	No main/simple effect
J. Lammers, D.A. Stapel, Power increases dehumanization. *Group Process. Intergr. Relat.* **14**, 113–126 (2011).	1	Power not manipulated
J. Lammers, D.A. Stapel, A.D. Galinsky, Power increases hypocrisy: Moralizing in reasoning, immorality in behavior. *Psychol. Sci.* **21**, 737–744 (2010).	2	Non-significant result
J. Lammers, J.I. Stoker, J. Jordan, M. Pollmann, D.A. Stapel, Power increases infidelity among men and women. *Psychol. Sci.* **22**, 1191–1197 (2011).	only study	Power not manipulated
J. Lammers, J.I. Stoker, F. Rink, A.D. Galinsky, To have control over or to be free from others? The desire for power reflects a need for autonomy. *Pers. Soc. Psychol. Bull.* **42**, 498–512 (2016).	1a-b, 4a-b, 5	Power not manipulated
J. Lammers, J.I. Stoker, D.A. Stapel, Differentiating social and personal power: Opposite effects on stereotyping, but parallel	1	Missing high- or low-power condition

effects on behavioral approach tendencies. *Psychol. Sci.* **20**, 1543–1548 (2009).		
J. Lammers, J.I. Stoker, D.A. Stapel, Differentiating social and personal power: Opposite effects on stereotyping, but parallel effects on behavioral approach tendencies. *Psychol. Sci.* **20**, 1543–1548 (2009).	2	Power not manipulated
J. Lammers, J.I. Stoker, D.A. Stapel, Power and behavioral approach orientation in existing power relations and the mediating effect of income. *Eur. J. Soc. Psychol.* **40**, 543–551 (2010).	only study	Participants' power not manipulated
C.A. Langner, D. Keltner, Social power and emotional experience: Actor and partner effects within dyadic interactions. *J. Exp. Soc. Psychol.* **44**, 848–856 (2008).	1	Romantic relationship sample
C.A. Langner, D. Keltner, Social power and emotional experience: Actor and partner effects within dyadic interactions. *J. Exp. Soc. Psychol.* **44**, 848–856 (2008).	2	Inadequate statistics
K. Laurin, G.M. Fitzsimons, E.J. Finkel, K.L. Carswell, M.R. vanDellen, W. Hofmann, N.M. Lambert, P.W. Eastwick, F.D. Fincham, P.C. Brown,. Power and the pursuit of a partner's goals. *J. Pers. Soc. Psychol.* **110**, 840–868 (2016).	1-4	Romantic relationship sample
K. Laurin, G.M. Fitzsimons, E.J. Finkel, K.L. Carswell, M.R. vanDellen, W. Hofmann, N.M. Lambert, P.W. Eastwick, F.D. Fincham, P.C. Brown, Power and the pursuit of a partner's goals. *J. Pers. Soc. Psychol.* **110**, 840–868 (2016).	5	Group/team design
S. Leach, M. Weick, From grumpy to cheerful (and back): How power impacts mood in and across different contexts. *J. Exp. Soc. Psychol.* **79**, 107–114 (2018).	all	No main/simple effect
G.J. Lelieveld, E. Van Dijk, I. Van Beest, G.A. Van Kleef, Why anger and disappointment affect other's bargaining behavior differently: The moderating role of power and the mediating role of reciprocal and complementary emotions. *Pers. Soc. Psychol. Bull.* **38**, 1209–1221 (2012).	all	Participants' power not manipulated

H. Li, Y.R. Chen, J.A.D. Hildreth, J. A. D. Powerlessness also corrupts: Lower power increases self-promotional lying. *Organ. Sci*. **34**, 1422-1440 (2023).	1	Power not manipulated
Y. Liu, S. Chen, C. Bell, J. Tan, How do power and status differ in predicting unethical decisions? A cross-national comparison of China and Canada. *J. Bus. Ethics* **167**, 745–760 (2020).	Canadian sample	Non-significant result
Z. Liu, M. Luan, H. Li, J.I. Stoker, J. Lammers, Psychological power increases the desire for social distance but reduces the sense of social distance. *J. Exp. Soc. Psychol.*, (in press).	1, 6	Power not manipulated
M. Lücken, B. Simon, Cognitive and affective experiences of minority and majority members: The role of group size, status, and power. *J. Exp. Soc. Psychol.* **41**, 396–413 (2005).	1, 2, 4	Power not manipulated
J.C. Magee, A.D. Galinsky, D.H Gruenfeld, Power, propensity to negotiate, and moving first in competitive interactions. *Pers. Soc. Psychol. Bull.* **33**, 200–212 (2007).	2	Missing high- or low-power condition
J.C. Magee, F.J. Milliken, A.R. Lurie, Power differences in the construal of a crisis: The immediate aftermath of September 11, 2001. *Pers. Soc. Psychol. Bull.* **36**, 354–370 (2010).	only study	Power not manipulated
J.K. Maner, M.T. Gailliot, D.A. Butz, B.M. Peruche, Power, risk, and the status quo: Does power promote riskier or more conservative decision making? *Pers. Soc. Psychol. Bull.* **33**, 451–462 (2007).	all	Missing high- or low-power condition
J.K. Maner, M.P. Kaschak, J.L. Jones, Social power and the advent of action. *Soc. Cogn.* **28**, 122–132 (2010).	only study	Missing high- or low-power condition
J.K. Maner, N.L. Mead, The essential tension between leadership and power: When leaders sacrifice group goals for the sake of self-interest. *J. Pers. Soc. Psychol.* **99**, 482–497 (2010).	all	Missing high- or low-power condition
E.A. Mannix, M.A. Neale, Power imbalance and the pattern of exchange in dyadic negotiation. *Group Decis. Negot.* **2**, 119–133 (1993).	only study	Missing high- or low-power condition
N.L. Mead, J.K. Maner, On keeping your enemies close: Powerful leaders seek proximity to ingroup power threats. *J. Pers. Soc. Psychol.* **102**, 576–591 (2012).	all	Missing high- or low-power condition
Y. Miyamoto, L.J. Li, Power fosters context-independent, analytic cognition. *Pers. Soc. Psychol. Bull.* **37**, 1449–1458 (2011).	3	Power not manipulated

M. Mooijman, Power dynamics and the reciprocation of trust and distrust. *J. Pers. Soc. Psychol.* **125**, 779–802 (2023).	all	Participants' power not manipulated
M. Mooijman, M. Kouchaki, E. Beall, J. Graham, Power decreases the moral condemnation of disgust-inducing transgressions. *Organ. Behav. Hum. Decis. Proc.* **161**, 79–92 (2020).	1, 6	Power not manipulated
M. Mooijman, M. Kouchaki, E. Beall, J. Graham, Power decreases the moral condemnation of disgust-inducing transgressions. *Organ. Behav. Hum. Decis. Proc.* **161**, 79–92 (2020).	2, 5, 7	Missing high- or low-power condition
T. Nissan, O. Shapira, N. Liberman, Effects of Power on Mental Rotation and Emotion Recognition in Women. *Pers. Soc. Psychol. Bull.* **41**, 1425–1437 (2015).	2-4	All women sample
J.R. Overbeck, M.A. Neale, C.L. Govan, I feel, therefore you act: Intrapersonal and interpersonal effects of emotion on negotiation as a function of social power. *Organ. Behav. Hum. Decis. Proc.* **112**, 126–139 (2010).	only study	Non-significant result
J.R. Overbeck, B. Park, When power does not corrupt: Superior individuation processes among powerful perceivers. *J. Pers. Soc. Psychol.* **81**, 549–565 (2001).	3	No main/simple effect
J.R. Overbeck, B. Park, Powerful perceivers, powerless objects: Flexibility of powerholders' social attention. *Organ. Behav. Hum. Decis. Proc.* **99**, 227–243 (2006).	1	Missing high- or low-power condition
J.R. Overbeck, B. Park, Powerful perceivers, powerless objects: Flexibility of powerholders' social attention. *Organ. Behav. Hum. Decis. Proc.* **99**, 227–243 (2006).	2	No main/simple effect
J. Pai, J. Whitson, J. Kim, S. Lee, A relational account of low power: The role of the attachment system in reduced proactivity. *Organ. Behav. Hum. Decis. Proc*. **167**, 28-41 (2021).	2, 4	Power not manipulated
J. Pai, J. Whitson, J. Kim, S. Lee, A relational account of low power: The role of the attachment system in reduced proactivity. *Organ. Behav. Hum. Decis. Proc*. **167**, 28-41 (2021).	3, 5	Missing high- or low-power condition
K. Petkanopoulou, R. Rodríguez-Bailón, G.B. Willis, G.A. van Kleef, Powerless people don't yell but tell: The effects of social power on direct and indirect expression of anger. *Eur. J. Soc. Psychol.* **49**, 533–547 (2019).	3	No main/simple effect

R.L. Pinkley, M.A. Neale, R.J. Bennett, Impact of knowledge regarding alternatives to settlement in dyadic negotiations: Whose knowledge counts? *Organ. Behav. Hum. Decis. Proc.*, **57**, 97-116 (1995).	2	No main/simple effect
N.J. Ratcliff, T.K. Vescio, Benevolently bowing out: The influence of self-construals and leadership performance on the willful relinquishing of power. *J. Exp. Soc. Psychol.* **49**, 978–983 (2013).	all	Group/team design
F. Righetti, L.B. Luchies, S. van Gils, E.B. Slotter, B. Witcher, M. Kumashiro, The prosocial versus proself power holder: How power influences sacrifice in romantic relationships. *Pers. Soc. Psychol. Bull.* **41**, 779–790 (2015).	all	Romantic relationship sample
R. Rodríguez-Bailón, M. Moya, V. Yzerbyt, Why do superiors attend to the negative stereotypic information about their subordinates? Effects of power legitimacy on social perception. *Eur. J. Soc. Psychol*. **30**, 651–671 (2000).	1	No main/simple effect
R. Rodríguez-Bailón, M. Moya, V. Yzerbyt, Why do superiors attend to the negative stereotypic information about their subordinates? Effects of power legitimacy on social perception. *Eur. J. Soc. Psychol*. **30**, 651–671 (2000).	2	Missing high- or low-power condition
D.D. Rucker, A.D. Galinsky, Desire to acquire: Powerlessness and compensatory consumption. *J. Consum. Res.* **35**, 257–267 (2008).	2	No main/simple effect
D.D. Rucker, A.D. Galinsky, Desire to acquire: Powerlessness and compensatory consumption. *J. Consum. Res.* **35**, 257–267 (2008).	3	Missing high- or low-power condition
D.D. Rucker, A.D. Galinsky, Conspicuous consumption versus utilitarian ideals: How different levels of power shape consumer behavior. *J. Exp. Soc. Psychol.* **45**, 549–555 (2009).	1	Participants' power not manipulated
D.D. Rucker, A.D. Galinsky, D. Dubois, Power and consumer behavior: How power shapes who and what consumers value. *J. Consum. Psychol.* **22**, 352–368 (2012).	only study	No main/simple effect
D.D. Rucker, M. Hu, A.D. Galinsky, The experience versus the expectations of power: A recipe for altering the effects of power on behavior. *J. Consum. Res.* **41**, 381–396 (2014).	1b	No main/simple effect

D. Rus, D. van Knippenberg, B. Wisse, Leader power and self-serving behavior: The moderating role of accountability. *Leadersh. Q.* **23**, 13–26 (2012).	3	Power not manipulated
T. Sawaoka, B.L. Hughes, N. Ambady, Power heightens sensitivity to unfairness against the self. Personal. *Soc. Psychol. Bull*. **41**, 1023–1035 (2015).	1, 3, 4	No main/simple effect
T. Sawaoka, B.L. Hughes, N. Ambady, Power heightens sensitivity to unfairness against the self. Personal. *Soc. Psychol. Bull*. **41**, 1023–1035 (2015).	2	Missing high- or low-power condition
M. Schaerer, R.I. Swaab, A.D. Galinsky, Anchors weigh more than power: Why absolute powerlessness liberates negotiators to achieve better outcomes. *Psychol. Sci.* **26**, 170-181 (2015).	2, 4	Missing high- or low-power condition
D. Scheepers, F. de Wit, N. Ellemers, K. Sassenberg, Social power makes the heart work more efficiently: Evidence from cardiovascular markers of challenge and threat. *J. Exp. Soc. Psychol.* **48**, 371–374 (2012).	all	Physiological measures
P.C. Schmid, D.M. Amodio, Power effects on implicit prejudice and stereotyping: The role of intergroup face processing. *Soc. Neurosci.* **12**, 218–241 (2017).	2	Physiological measures
P.C. Schmid, L.M. Hackel, D.M. Amodio, Power effects on instrumental learning: Evidence from the brain and behavior. *Motiv. Sci.* **4**, 206–226 (2018).	2	Non-significant result
P.C. Schmid, M. Schmid Mast, Power increases performance in a social evaluation situation as a result of decreased stress responses. *Eur. J. Soc. Psychol.* **43**, 201–211 (2013).	all	Missing high- or low-power condition
M. Schmid Mast, K. Jonas, J.A. Hall, Give a person power and he or she will show interpersonal sensitivity: The phenomenon and its why and when. *J. Pers. Soc. Psychol.* **97**, 835–850 (2009).	1	Leadership/status conflated with power
M. Schmid Mast, K. Jonas, J.A. Hall, Give a person power and he or she will show interpersonal sensitivity: The phenomenon and its why and when. *J. Pers. Soc. Psychol.* **97**, 835–850 (2009).	4	Power not manipulated
A. Scholl, K. Sassenberg, Where could we stand if I had…? How social power impacts counterfactual thinking after failure. *J. Exp. Soc. Psychol.* **53**, 51–61 (2014).	1	Power not manipulated

A. Scholl, K. Sassenberg, Where could we stand if I had…? How social power impacts counterfactual thinking after failure. *J. Exp. Soc. Psychol.* **53**, 51–61 (2014).	3	No main/simple effect
A. Scholl, K. Sassenberg, N. Ellemers, D. Scheepers, F. De Wit, Highly identified power-holders feel responsible: The interplay between social identification and social power within groups. *British J. Soc. Psychol.* **57**, 112-129 (2018).	1	Power not manipulated
T.W. Schubert, The power in your hand: Gender differences in bodily feedback from making a fist. *Pers. Soc. Psychol. Bull.* **30**, 757–769 (2004).	all	Power not manipulated
T.W. Schubert, Your highness: Vertical positions as perceptual symbols of power. *J. Pers. Soc. Psychol.* **89**, 1–21 (2005).	all	Participants' power not manipulated
T.W. Schubert, The embodied self: Making a fist enhances men's power-related self-conceptions. *J. Exp. Soc. Psychol.* **45**, 828–834 (2009).	all	Power not manipulated
K.E. See, E.W. Morrison, N.B. Rothman, J.B. Soll, The detrimental effects of power on confidence, advice taking, and accuracy. *Organ. Behav. Hum. Decis. Proc.* **116**, 272–285 (2011).	1, 2	Power not manipulated
N. Sivanathan, M.M. Pillutla, J. Keith Murnighan, Power gained, power lost. *Organ. Behav. Hum. Decis. Proc.* **105**, 135–146 (2008).	all	Missing high- or low-power condition
D.J. Sligte, C.K. de Dreu, B.A. Nijstad, Power, stability of power, and creativity. *J. Exp. Soc. Psychol.* **47**, 891–897 (2011).	all	No main/simple effect
P.K. Smith, J.A. Bargh, Nonconscious effects of power on basic approach and avoidance tendencies. *Soc. Cogn.* **26**, 1–24 (2008).	3	Non-significant result
P.K. Smith, N.B. Jostmann, A.D. Galinsky, W.W. van Dijk, Lacking power impairs executive functions. *Psychol. Sci.* **19**, 441–447 (2008).	3	Non-significant result
P.K. Smith, Y. Trope, You focus on the forest when you're in charge of the trees: Power priming and abstract information processing. *J. Pers. Soc. Psychol.* **90**, 578–596 (2006).	7	Neuroimaging
L. Solomon, The influence of some types of power relationships and game strategies upon the development of interpersonal trust. *J. Abnorm. and Soc. Psychol.* **61**, 223-230 (1960).	only study	Missing high- or low-power condition

H. Sondak, M.H. Bazerman, Power balance and the rationality of outcomes in matching markets. *Organ. Behav. Hum. Decis. Proc.* **50**, 1–23 (1991).	only study	Missing high- or low-power condition
E. Stamkou, G.A. van Kleef, A.H. Fischer, M.E. Kret, Are the powerful really blind to the feelings of others? How hierarchical concerns shape attention to emotions. *Pers. Soc. Psychol. Bull.* **42**, 755–768 (2016).	all	No main/simple effect
M. Stel, E.V. Dijk, P.K. Smith, W.W.V. Dijk, F.M. Djalal, Lowering the pitch of your voice makes you feel more powerful and think more abstractly. *Soc. Psychol. Pers. Sci.* **3**, 497–502 (2012).	all	Power not manipulated
C.W. Struthers, C.H. Khoury, C.E. Phills, E. van Monsjou, J.R. Guilfoyle, K. Nash, ... & C. Summers, The effects of social power and apology on victims' post-transgression responses. *J. Exp. Psychol: Appl.*, **25**, 100-116 (2019).	1, 4	Power not manipulated
C.W. Struthers, C.H. Khoury, C.E. Phills, E. van Monsjou, J.R. Guilfoyle, K. Nash, ... & C. Summers, The effects of social power and apology on victims' posttransgression responses. *J. Exp. Psychol: Appl.*, **25**, 100-116 (2019).	3, 5	No main/simple effect
C.J. Torelli, S. Shavitt, Culture and concepts of power. *J. Pers. Soc. Psychol.* **99**, 703–723 (2010).	all	Power not manipulated
A.K. Uskul, S. Paulmann, M. Weick, Social power and recognition of emotional prosody: High power is associated with lower recognition accuracy than low power. *Emotion* **16**, 11 (2016).	1	Participants' power not manipulated
M. Van Dijke, M. Poppe, Striving for personal power as a basis for social power dynamics. *Eur. J. Soc. Psychol.* **36**, 537–556 (2006).	2	No main/simple effect
G.A. van Kleef, C.K. de Dreu, D. Pietroni, A.S. Manstead, Power and emotion in negotiation: Power moderates the interpersonal effects of anger and happiness on concession making. *Eur. J. Soc. Psychol.* **36**, 557–581 (2006).	2	Power not manipulated
G.A. van Kleef, C. Oveis, I. van der Löwe, A. LuoKogan, D. Keltner, Power, distress, and compassion. *Psychol. Sci.* **19**, 1315–1322 (2008).	only study	Power not manipulated
K.J. Van Loo, R.J. Rydell, On the experience of feeling powerful: Perceived power moderates the effect of stereotype threat on	all	All women sample

women's math performance. *Pers. Soc. Psychol. Bull.* **39**, 387–400 (2013).		
T.K. Vescio, M. Snyder, D.A. Butz, Power in stereotypically masculine domains: A social influence strategy x stereotype match model. *J. Pers. Soc. Psychol.* **85**, 1062–1078 (2003).	all	Power not manipulated
Y.N. Wang, Authenticity and relationship satisfaction: Two distinct ways of directing power to self-esteem. *PLOS ONE* **10**, e0146050 (2015).	all	Power not manipulated
M. Weick, A. Guinote, When subjective experiences matter: Power increases reliance on the ease of retrieval. *J. Pers. Soc. Psychol.* **94**, 956–970 (2008).	1a-b, 3	No main/simple effect
M. Weick, A. Guinote, When subjective experiences matter: Power increases reliance on the ease of retrieval. *J. Pers. Soc. Psychol.* **94**, 956–970 (2008).	2, 4	Power not manipulated
A. Weick, A. Guinote, How long will it take? Power biases time predictions. *J. Exp. Soc. Psychol.* **46**, 595–604 (2010).	1	Missing high- or low-power condition
A. Weick, A. Guinote, How long will it take? Power biases time predictions. *J. Exp. Soc. Psychol.* **46**, 595–604 (2010).	4	Power not manipulated
D. Wilkinson, A. Guinote, M. Weick, R. Molinari, K. Graham, Feeling socially powerless makes you more prone to bumping into things on the right and induces leftward line bisection error. *Psychon. Bull. Rev.* **17**, 910–914 (2010).	only study	Neuroimaging
G.B. Willis, A. Guinote, R. Rodríguez-Bailón, Illegitimacy improves goal pursuit in powerless individuals. *J. Exp. Soc. Psychol.* **46**, 416–419 (2010).	all	Missing high- or low-power condition
G.B. Willis, R. Rodríguez-Bailón, J. Lupiáñez, The boss is paying attention: Power affects the functioning of the attentional networks. *Soc. Cogn.* **29**, 166–181 (2011).	1	No main/simple effect
K.A. Woltin, A. Guinote, I can, I do, and so I like: From power to action and aesthetic preferences. *J. Exp. Psychol. Gen*. **144**, 1124–1136 (2015).	1, 2, 4	No main/simple effect
S. Worchel, S.E. Arnold, W. Harrison, Aggression and power restoration: The effects of identifiability and timing on aggressive behavior. *J. Exp. Soc. Psychol.* **14**, 43–52 (1978).	only study	Missing high- or low-power condition

P. Yang, Q. Zhang, How pride influences product evaluation through construal level. *Eur. J. Market*., **52**, 1750-1775 (2018).	1-4b	Power not manipulated
P. Yang, Q. Zhang, How pride influences product evaluation through construal level. *Eur. J. Market*., **52**, 1750-1775 (2018).	5a	No main/simple effect
P. Yang, Q. Zhang, How pride influences product evaluation through construal level. *Eur. J. Market*., **52**, 1750-1775 (2018).	5c	Non-significant result
S.J. Zaccaro, R.J. Foti, D.A. Kenny, Self-monitoring and trait-based variance in leadership: An investigation of leader flexibility across multiple group situations. *J. Appl. Psychol.* **76**, 308–315 (1991).	1	Power not manipulated
M. Zheng, A. Guinote, How power affects moral judgments: The role of intuitive thinking. *Soc. Behav. Personal.* **50**, 19-30 (2022).	2	Missing high- or low-power condition
X. Zheng, M. van Dijke, J.M. Leunissen, L.M. Giurge, D. De Cremer, When saying sorry may not help: Transgressor power moderates the effect of an apology on forgiveness in the workplace. *Hum. Relat.* **69**, 1387-1418 (2016).	1	Power not manipulated
X. Zheng, M. van Dijke, J.M. Leunissen, L.M. Giurge, D. De Cremer, When saying sorry may not help: Transgressor power moderates the effect of an apology on forgiveness in the workplace. *Hum. Relat.* **69**, 1387-1418 (2016).	2, 3	Participants’ power not manipulated
Note. This list is almost certainly incomplete. If a study on power is located but not listed here, and also not listed in Table SI2, then it was not included in this analysis.		

Table SI4. Constructs relabeled from Archer (2019)

**Construct Label in Current Analysis**	**Archer (2019) Label**
Academic dishonesty	Cheating
Achievement learning orientation	Learning orientation (achievement)
Affiliation motivation	Implicit affiliation motivation
Anger-related emotions	Anger (frequency)
Anxiety-related emotions	Social anxiety
Attitudes toward academic dishonesty	Attitudes to cheating
Autocratic (vs. democratic) leadership style	Democratic leadership style (reverse sign)
Care-based moral judgement	Moral orientation (care)
Empathic accuracy	Decoding non-verbal cues
Fear	Fear questionnaires
Impulsivity	Impulsivity (overall)
Negative affect	Negative emotions overall
Norm-based (vs consequence-based) moral judgment	Moral norms vs. consequences
Person orientation	Person-things dimension
Positive affect	Positive emotions
Punishment sensitivity	Harm avoidance
Reaction time	Simple reaction time
Reward sensitivity	Reward sensitivity (overall)
Self-disclosure	Disclosure (to same sex)
Social vocational interests	Social interests
Spatial perception	Visuospatial ability
Susceptibility to influence	Influencing others (reverse sign)
Task-oriented leadership	Leadership (task)
Temporal discounting	Delay of gratification
Visual-spatial working memory	Working memory (spatial)

Table SI5. Constructs relabeled from Wilmot et al. (2019)

**Construct Label in Current Analysis**	**Wilmot et al. (2019) Label**
Change-oriented organizational citizenship behavior	Organizational citizenship behavior: change
Conservation value: conformity	Personal values: conservation: conformity
Conservation value: security	Personal values: conservation: security
Friendship network centrality	Social network roles: expressive: in-degree
Interpersonal counterproductive work behavior	Counterproductive work behavior: interpersonal
Job satisfaction	Job satisfaction (overall)
Openness-to-change value: self-direction	Personal values: openness to change: self-direction
Openness-to-change value: stimulation	Personal values: openness to change: stimulation
Personal-emotional adjustment to college	Adjustment to college: personal emotional
Self-enhancement value: achievement	Personal values: self-enhancement: achievement
Self-enhancement value: power	Personal values: self-enhancement: power
Self-transcendence value: benevolence	Personal values: self-transcendence: benevolence
Social adjustment to college	Adjustment to college: social
Social vocational interests	Vocational interests: social

Table SI6. Effect size modification procedures for constructs [with any new labels] from sex/gender and extraversion meta-analyses.

**Sex/Gender Construct**	**Source**	**Steps Taken to Recalculate Effect Size**	**Original *d***	**Modified *d***
Academic self-efficacy	C. Huang, Gender differences in academic self-efficacy: A meta-analysis. *Eur. J. Soc. Psychol.* **28**, 1-35 (2013).	Excluded studies of child, adolescent, and samples of unknown age. Recomputed average effect size.	.08[a]	.14[a]
Authentic pride<br>Embarrassment<br>Guilt<br>Hubristic pride<br>Shame	N. M. Else-Quest, A. Higgins, C. Allison, L. C. Morton, Gender differences in self-conscious emotional experience: a meta-analysis. *Psychol. Bull*. **138**, 947-981 (2012).	Excluded studies of childhood, adolescence, and late adulthood (i.e., older adults) samples. Recomputed average effect size for each of five constructs.	-.01<br>-.08<br>-.27<br>.09<br>-.29	.02<br>-.04<br>-.28<br>.11<br>-.30
Body appreciation [physical appearance self-esteem]	J. He, S. Sun, H. F. Zickgraf, Z. Lin, X. Fan, Meta-analysis of gender differences in body appreciation. *Body Image* **33**, 90-100 (2020).	Excluded studies of primary, middle, and high school samples (i.e., children and adolescents). Recomputed average effect size.	.27	.23
Counterproductive work behavior targeted at individuals [interpersonal counterproductive work behavior]<br>Organizational citizenship behavior targeted at individuals [interpersonal citizenship behavior]	T. W. Ng, S. S. Lam, D. C. Feldman, Organizational citizenship behavior and counterproductive work behavior: Do males and females differ? *J. Vocat. Behav*. **93**, 11-32 (2016).	Computed average of self-/peers'/supervisor's ratings effect sizes for each of two constructs.	.24/<br>.32/<br>.06<br><br>.00/<br>.10/<br>-.06	.24<br><br><br><br>-.02
Delay discounting	C. P. Cross, L. T. Copping, A. Campbell, Sex differences in impulsivity: A meta-analysis. *Psychol. Bull.* ***137***, 97–130 (2011).<br><br>A. Gaillard, D. J. Fehring, S. L. Rossell, Sex differences in executive	From Cross et al., excluded studies of child and adolescent samples. Computed average of remaining delay discounting effect sizes from Cross et al. and delay discounting studies analyzed by Gaillard et al., treating Hedges' *g* as equivalent to Cohen's *d*.	-.08[b]<br>-.64[c]	-.14

	control: A systematic review of functional neuroimaging studies. *Eur. J. Neurosci*. **53**, 2592-2611 (2021).			
Empathic accuracy	A. E. Thompson, D. Voyer, Sex differences in the ability to recognise non-verbal displays of emotion: A meta-analysis. *Cogn. Emot.* **28**, 1164-1195 (2014).	Excluded effect sizes for children and adolescents. Recomputed average effect size.	-.19	-.22
Face recognition	A. Herlitz, J. Lovén, Sex differences and the own-gender bias in face recognition: A meta-analytic review. *Vis. Cgn.* **21**, 1306-1336 (2013).	Included only the effect size for adults (i.e., excluded children and adolescents). (This happens to be equal to the overall effect size including all age groups.)	-.36[a]	-.36[a]
Loneliness	M. Maes, P. Qualter, J. Vanhalst, W. Van den Noortgate, L. Goossens, Gender differences in loneliness across the lifespan: A meta–analysis. *Eur. J. Pers*. **33**, 642-654 (2019).	Excluded studies of clinical (and mixed clinical/non-clinical), child, adolescent, and elderly (i.e., older adult) samples. Also, excluded measures related to romantic relationships. For studies using multiple measures, included only one effect per study, and selected a measure related to Peer over Family relationships; and Peer, Social, or Friends over other subscales. Computed average of remaining effect sizes.	.07[a]	.12[a]
Mental rotation<br>Spatial perception<br>Spatial visualization	D. Voyer, S. Voyer, M. P. Bryden,. Magnitude of sex differences in spatial abilities: A meta-analysis and consideration of critical variables. *Psychol. Bull.* ***117***, 250–270 (1995).	Included only effect sizes for samples > 18 years of age (i.e., excluded children and adolescents) for each of three constructs.	.56<br>.44<br>.13	.66<br>.48<br>.23

Nightmare frequency	M. Schredl, I. Reinhard, Gender differences in nightmare frequency: A meta-analysis. *Sleep Med. Rev.* **15**, 115-121 (2011).	Excluded children, adolescents, and older adults. Included young adults and middle-aged adults (Archer [2019] only included young adults.) Recomputed average effect size.	-.26[a]	-.20[a]
Organizational citizenship behavior targeted at tasks [task-based organizational citizenship behavior]<br>Voice behavior [voice]	T. W. Ng, S. S. Lam, D. C. Feldman, Organizational citizenship behavior and counterproductive work behavior: Do males and females differ? *J. Vocat. Behav*. **93**, 11-32 (2016).	Computed average of self-/supervisor's ratings effect sizes for each of two constructs.	-.06/<br>-.24<br><br>.06/<br>.00	-.16<br><br><br>.04
Prejudice	N. Dozo, "Gender differences in prejudice: A biological and social psychological analysis," University of Queensland, Australia (2015).	Excluded homosexual prejudice (i.e., a measure related to sexuality) effect size. Recomputed average effect size.	.20	.16
Response conflict (executive function)	C. P. Cross, L. T. Copping, A. Campbell, Sex differences in impulsivity: A meta-analysis. *Psychol. Bull.* **137**, 97–130 (2011).<br><br>A. Gaillard, D. J. Fehring, S. L. Rossell, Sex differences in executive control: A systematic review of functional neuroimaging studies. *Eur. J. Neurosci*. **53**, 2592-2611 (2021).	From Cross et al., excluded studies of response inhibition using child and adolescent samples. From Gaillard et al., included only response inhibition techniques that matched either the power studies on this construct (Flanker, Stroop) or the studies analyzed in Cross et al. (Stroop, Go/No Go, Continuous Performance Task, Stop Signal). Treating Hedges' *g* as equivalent to Cohen's *d*, computed average of those included effect sizes from Cross et al. and Gaillard et al.	.13[b]<br>.01[c]	-.02
Reward sensitivity	C. P. Cross, L. T. Copping, A. Campbell, Sex differences in impulsivity: A meta-analysis. *Psychol. Bull.* **137**, 97–130 (2011).	Excluded studies using TPQ/TCI Reward Dependence because, as Archer (2019) notes in the Supplement, this is clearly a measure of sociability, not reward sensitivity. Recomputed average effect size.	-.56	.19

Resistance to temptation	I. W. Silverman, Gender differences in resistance to temptation: Theories and evidence. *Dev. Rev*. **23**, 219-259 (2003).	Excluded effect sizes for children and adolescents. Recomputed average effect size.	-.06	-.30
Risk-taking	A. Filippin, P. Crosetto, A reconsideration of gender differences in risk attitudes. *Manag. Sci*. **62**, 3138-3160 (2016).	Computed sample-size weighted average of effect sizes for Holt & Laury task (which the authors report), Investment Game, and Eckel & Grossman task.	.17	.31
Self-confidence in physical activity	C. D. Lirgg, Gender differences in self-confidence in physical activity: A meta-analysis of recent studies. *J. Sport Exerc. Psychol*. **13**, 294–310 (1991).	Excluded studies of elementary, junior high, high school students, "mixed", or unknown samples. For studies using multiple measures, included only one effect per study, and chose the most general measure, or smallest effect if there was no difference in generality. Recomputed average effect size.	.40	.47
Self-disclosure	K. Dindia, M. Allen, Sex differences in self-disclosure: A meta-analysis. *Psychol. Bull.* **112**, 106-124 (1992).	Included same-sex and opposite sex interactions. Excluded spousal (i.e., romantic) relationship to target. Recomputed average effect size.	-.37	-.18
Self-esteem	M. Zuckerman, C. Li, J. A. Hall, When men and women differ in self-esteem and when they don't: A meta-analysis. *J. Res. Pers*. **64**, 34-51 (2016).	Excluded studies of child, adolescent, and old-age samples. Recomputed average effect size.	.11[a]	.08[a]
Self-rated overall intelligence<br>Self-rated mathematical/logical intelligence<br>Self-rated spatial intelligence<br>Self-rated verbal intelligence	A. Syzmanowicz, A. Furnham, Gender differences in self-estimates of general, mathematical, spatial and verbal intelligence: Four meta analyses. *Learn. Individ. Differ*. **21**, 493–504 (2011).	Excluded effect sizes for adolescents. Recomputed average effect size for each of four constructs.	.37<br>.44<br><br>.43<br>.07	.37<br>.47<br><br>.50<br>.15

Stress	M. C. Davis, K. A. Matthews, E. W. Twamley, Is life more difficult on Mars or Venus? A meta-analytic review of sex differences in major and minor life events. *Ann. Behav. Med.* **21**, 83-97 (1999).	Included only stress appraisal (excluded stress exposure), and only effect sizes for young adults and adults (i.e., excluded children and adolescents). Also, excluded "unusual samples," some of which were clinical.	-.18	-.17
Temporal discounting	J. L. Doidge, D. B. Flora, M. E. Toplak, A meta-analytic review of sex differences on delay of gratification and temporal discounting tasks in ADHD and typically developing samples. *J. Atten. Disord*. **25**, 540-561 (2021).	Selected exclusively "typically developing" participants and not those with ADHD. Excluded effect sizes for children and adolescents. Recomputed average effect size.	-.01[a]	-.03[a]
Trust in others<br><br>Trustworthiness	O.R. van den Akker, M.A. van Assen, M. Van Vugt, J.M. Wicherts, Sex differences in trust and trustworthiness: A meta-analysis of the trust game and the gift-exchange game. *J. Econ. Psychol.* **81**, 102329 (2020).	Excluded one old-age sample and one study that involved power as an independent variable. Computed k-weighted average of effect sizes for trust game / bilateral gift exchange game.	.22[a]/<br>.15[a]<br>-.04[a]/<br>.33[a]	.22[a]<br><br>.02[a]
Visual-spatial working memory (general)<br>Visual-spatial working memory (location only)	D. Voyer, S. D. Voyer, J. Saint-Aubin, Sex differences in visual-spatial working memory: A meta-analysis. *Psychon. Bull. Rev*. **24**, 307-334 (2017).	Excluded children, adolescents, and old-age samples. Recomputed average effects size for each of two constructs.	.21<br><br>-.34	.30<br><br>-.28
**Extraversion Construct**				
Abstract reasoning<br>Mental rotation<br>Reaction time<br>Spatial visualization	K. C. Stanek, D. S. Ones, Meta-analytic relations between personality and cognitive ability. *Proc. Natl. Acad. Sci. U.S.A.* **120**.	Excluded adolescent and old-age samples. Included Extraversion measures from only the following: Eysenck Personality Inventory/Questionnaire, Big Five Inventory/Questionnaire, Ten Item	NA[d]<br>NA[d]<br>.02<br>NA[d]	.12<br>-.18<br>.04<br>-.06

		Personality Inventory, NEO, International Personality Item Pool (if multiple measures reported for same sample, NEO or Big Five was included and others excluded). If multiple measures of outcome variable reported, they were averaged. Abstract reasoning includes only Raven's progressive matrices measures. Mental rotation and spatial visualization include only measures included in Voyer, Voyer, & Bryden's (1995) sex/gender difference meta-analysis of spatial abilities. Recomputed average effect size from observed *r* for each of four constructs.		
Anger-related emotions<br>Caring emotions<br>Fear<br>Playful emotions<br>Sadness<br>Seeking emotions	D. Marengo, K. L. Davis, G. Ö. Gradwohl, C. Montag, A meta-analysis on individual differences in primary emotional systems and Big Five personality traits. *Sci. Rep*. **11**, 7453 (2021).	Excluded clinical samples. Recomputed average effect size separately for each of five constructs.	-.10<br>.56<br>-.58<br>1.32<br>-.47<br>.72	-.10<br>.56<br>-.57<br>1.27<br>-.48<br>.68
Prosocial behavior	I. Thielmann, G. Spadaro, D. Balliet, Personality and prosocial behavior: A theoretical framework and meta-analysis. *Psychol. Bull.* **146**, 30-90 (2020).	Removed trust game effect sizes (which were used for "Trust in others" and "Trustworthiness"). Recomputed average effect size.	.02	.00
Resilience	A. Oshio, K. Taku, M. Hirano, G. Saeed, Resilience and big five personality traits: A meta-analysis. *Pers. Individ. Differ.* **127**, 54-60 (2018).	Excluded adolescent and clinical samples. Recomputed average effect size.	.93	.95

Revenge	E. Mullet, F. Neto, S. Rivière, "Personality and its effects on resentment, revenge, forgiveness, and self-forgiveness" in *Handbook of Forgiveness*, E. L. Worthington, Jr., Ed (Routledge, 2005), pp. 159-181.	Excluded clinical samples. Converted any standardized regression coefficients to effect sizes (r) based on https://www.psychometrica.de/effect_size.html. Recomputed average effect size.	-.14	-.05
Stress	J. Luo, B. Zhang, M. Cao, B. W. Roberts, The stressful personality: A meta-analytical review of the relation between personality and stress. *Pers. Soc. Psychol. Rev*. **27**, 128-194 (2023).	Excluded physiological measures. Recomputed average effect size.	-.22	-.22

Note. Details for all recalculations are available from the last author.
[a] Hedges' *g*
[b] Effect size reported in Cross et al. (2011)
[c] Effect size reported in Gaillard et al. (2021)
[d] Stanek and Ones (2023) do not report effect sizes for these three measures because they view them as measures of higher-order constructs. The authors report effect sizes for the following constructs that include the measures we extracted: fluid induction (including progressive matrices measures of abstract reasoning), *d* = .00; visualization (including mental rotation measures), *d* = -.03; flexibility of closure (including spatial visualization measures), *d* = -.04.

Table S17. Comparison of extraversion effect sizes and sex/gender differences from meta-analyses

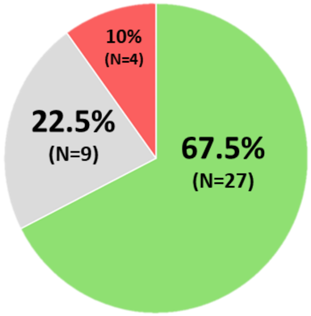

Level III Category: **Agency**

Sex/Gender Difference Consistent with Extraversion Effect

No Sex/Gender Difference

Sex/Gender Difference Inconsistent with Extraversion Effect

Level II Construct	Level I Construct	Extraversion Meta-Analytic Effect Size[a]	Results in relation to sex/gender differences[b]	
**Dominance**	Self-enhancement value: power[1]	0.47	C	**Consistent Effect = 3**<br>**No Effect = 4**<br>**Inconsistent Effect = 3**
	Hubristic pride[2]	0.12	C	
	Narcissism[3]	0.65	C	
	Revenge[4]	-0.05[c]	N	
	Aggression[5]	0.07	N	
	Employee entitlement[6]	0.02	N	
	Interpersonal counterproductive work behavior[1]	0.04	N	
	Anger-related emotions[7]	-0.10[c]	I	
	Forcing conflict resolution style[8]	-0.35	I	
	Forgiveness[9]	0.18	I	
**Goal Approach & Disinhibition**	Self-enhancement value: achievement[1]	0.47	C	**Consistent Effect = 20**<br>**No Effect = 3**<br>**Inconsistent Effect = 1**
	Proactive personality[10]	0.72	C	
	Change-oriented behavior[1]	0.41	C	
	Change-oriented organizational citizenship behavior[1]	0.20	C	
	Employee engagement (overall)[1]	0.68	C	
	Demonstrating effort[1]	0.28	C	
	Adaptive performance[1]	0.16	C	
	Job search self-regulation[1]	0.32	C	
	Procrastination[1]	-0.22	C	

	Goal orientation: learning[1]	0.49	C	
	Voice[1]	0.37	C	
	Promotion regulatory focus[1]	0.63	C	
	Self-leadership[11]	0.52	C	
	Openness-to-change value: self-direction[1]	0.24	C	
	Career adaptability[1]	0.65	C	
	Contextual performance[1]	0.37	C	
	Job crafting[1]	0.39	C	
	Job search intensity[1]	0.10	C	
	Performance motivation: expectancy[1]	0.14	C	
	Performance motivation: goal-setting[1]	0.26	C	
	Academic procrastination[1]	0.08	N	
	Goal orientation: performance prove[1]	0.06	N	
	Reaction time[12]	0.04	N	
	Study habits[1]	-0.22	I	
**Risk-Seeking**	Risk propensity[13]	0.43	C	**Consistent Effect = 4**<br>**No Effect = 2**<br>**Inconsistent Effect = 0**
	Engagement in high-risk sports[14]	0.39[d]	C	
	Openness-to-change value: stimulation[1]	0.58	C	
	Seeking emotions[7]	0.68[c]	C	
	Academic dishonesty[1]	0.08	N	
	Conservation value: security[1]	-0.08	N	

Level III Category: **Communion**

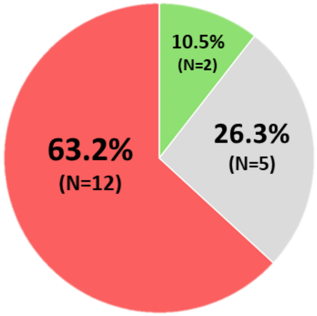

Sex/Gender Difference Consistent with Extraversion Effect

No Sex/Gender Difference

Sex/Gender Difference Inconsistent with Extraversion Effect

**Interpersonal sensitivity**	Conservation value: conformity[1]	-0.26	**C**	**Consistent Effect = 1**<br>**No Effect = 4**<br>**Inconsistent Effect = 7**
	Self-transcendence value: benevolence[1]	-0.08	**N**	
	Prosocial behavior[15]	0.00[c]	**N**	
	Trust in others[15]	0.06	**N**	
	Trustworthiness[15]	0.00	**N**	
	Cooperativeness[16]	0.26	**I**	
	Emotional expressiveness[1]	0.41	**I**	
	Caring emotions[7]	0.56[c]	**I**	
	Interpersonal sensitivity[1]	0.14	**I**	
	Transformational leadership: individualized consideration[1]	0.24	**I**	
	Interpersonal citizenship behavior[1]	0.14	**I**	
	Social vocational interests[1]	0.58	**I**	
**Sociability**	Social adjustment to college[1]	0.56	**I**	**Consistent Effect = 0**<br>**No Effect = 0**<br>**Inconsistent Effect = 5**
	"Getting along" performance[1]	0.14	**I**	
	Friendship network centrality[1]	0.18	**I**	
	Person orientation[17]	0.82	**I**	
	Playful emotions[7]	1.27	**I**	
**Prejudice & Dehumanization**	*Prejudice*[18]	0.14	**C**	**Consistent Effect = 1**<br>**No Effect = 1**<br>**Inconsistent Effect = 0**
	Social dominance orientation[18]	-0.06	**N**	

Level III Category: **Self-Evaluations**

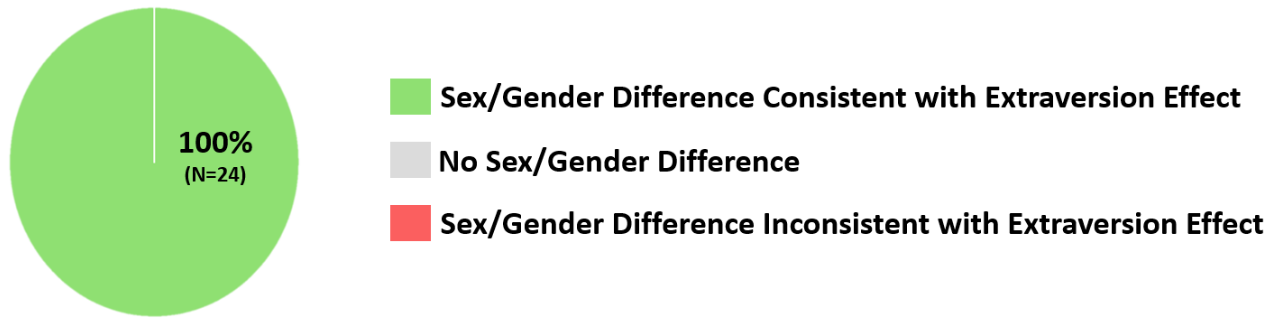

**Positive Views of Self**	Generalized self-efficacy[19]	0.72	**C**	
	Academic self-efficacy[1]	0.28	**C**	

	Career optimism[20]	0.68	C	Consistent Effect = 7<br>No Effect = 0<br>Inconsistent Effect = 0
	Performance motivation: self-efficacy[1]	0.49	C	
	Self-rated overall intelligence[21]	0.22	C	
	Physical appearance self-esteem[22]	0.49	C	
	Authentic pride[2]	0.87	C	
**Well-Being**	Psychological well-being[23]	0.85	C	Consistent Effect = 17<br>No Effect = 0<br>Inconsistent Effect = 0
	Loneliness[24]	-0.87	C	
	Positive affect[23]	0.98	C	
	Negative affect[23]	-0.43	C	
	Fear[7]	-0.57[c]	C	
	Happiness[1]	0.87	C	
	Sadness[7]	-0.48[c]	C	
	Rumination[25]	-0.18	C	
	Life satisfaction[23]	0.68	C	
	Stress[26]	-0.22[c]	C	
	Resilience[27]	0.95[c]	C	
	Job satisfaction[1]	0.39	C	
	Career satisfaction[1]	0.41	C	
	Academic satisfaction[1]	0.14	C	
	Burnout: emotional exhaustion[1]	-0.30	C	
	Burnout: depersonalization[1]	-0.37	C	
	Personal-emotional adjustment to college[1]	0.20	C	

Level III Category: **Cognitive Processes**

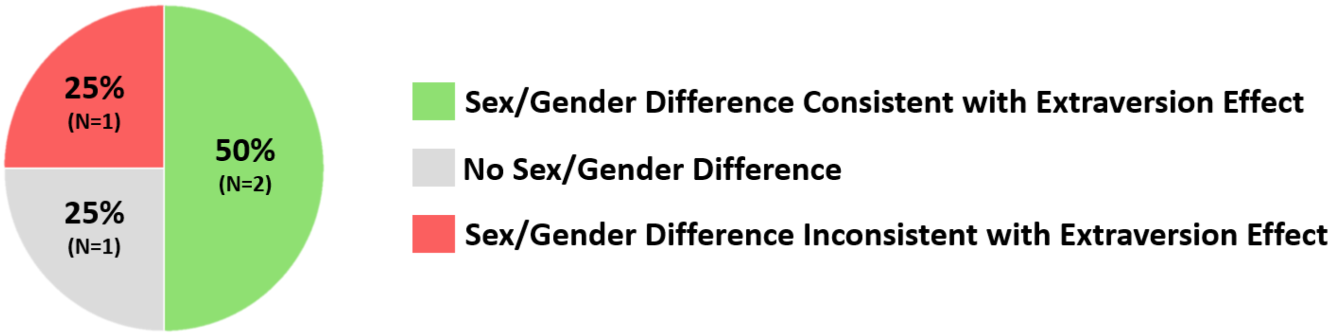

**Spatial Ability/ Performance**	*Spatial visualization*[12]<br>*Mental rotation*[12]	-0.06[c]<br>-0.18[c]	**N**<br>**I**	**Consistent Effect = 0**<br>**No Effect = 1**<br>**Inconsistent Effect = 1**
**Creative Performance**	*Creativity*[1]	0.26[c]	**C**	**Consistent Effect = 1**<br>**No Effect = 0**<br>**Inconsistent Effect = 0**
**Abstract Cognition**	*Abstract reasoning*[12]	0.12[c]	**C**	**Consistent Effect = 1**<br>**No Effect = 0**<br>**Inconsistent Effect = 0**

Note. An italicized Level I construct denotes a match with a sex/gender difference. These are used to compute the extraversion-sex/gender correlation.

[a] Effect sizes are Cohen's d, or conversions from *r* to *d*, except where noted.

[b] C = Consistent effect; N = No effect; I = Inconsistent effect

[c] Effect size recalculated from original source in order to follow inclusion/exclusion criteria (see Table SI6 for procedure)

[d] Hedges' *g*

Extraversion References

1. M. P. Wilmot, C. R. Wanberg, J. D. Kammeyer-Mueller, D. S. Ones, Extraversion advantages at work: A quantitative review and synthesis of the meta-analytic evidence. *Journal of Applied Psychology* **104**, 1447–1470 (2019).

2. L. R. Dickens, R. W. Robins, Pride: A meta-analytic project. *Emotion* **22**, 1071–1087 (2022).

3. P. Muris, H. Merckelbach, H. Otgaar, E. Meijer, The malevolent side of human nature: A meta-analysis and critical review of the literature on the dark triad (narcissism, machiavellianism, and psychopathy). *Perspect Psychol Sci* **12**, 183–204 (2017).

4. E. Mullet, F. Neto, S. Rivière, "Personality and its effects on resentment, revenge, forgiveness, and self-forgiveness" in *Handbook of Forgiveness*, (Routledge, 2005), pp. 159–182.

5. C. S. Hyatt, A. Zeichner, J. D. Miller, Laboratory aggression and personality traits: A meta-analytic review. *Psychology of Violence* **9**, 675–689 (2019).

6. A. Bedi, But I deserve it! A meta-analytic review of employee entitlement. *Journal of Managerial Issues: JMI* **33**, 191–209 (2021).

7. D. Marengo, K. L. Davis, G. Ö. Gradwohl, C. Montag, A meta-analysis on individual differences in primary emotional systems and Big Five personality traits. *Sci Rep* **11**, 7453 (2021).

8. H. D. Tehrani, S. Yamini, Personality traits and conflict resolution styles: A meta-analysis. *Personality and Individual Differences* **157**, 109794 (2020).

9. D. Balliet, Conscientiousness and forgivingness: A meta-analysis. *Personality and Individual Differences* **48**, 259–263 (2010).

10. J. P. Thomas, D. S. Whitman, C. Viswesvaran, Employee proactivity in organizations: A comparative meta-analysis of emergent proactive constructs. *Journal of Occupational and Organizational Psychology* **83**, 275–300 (2010).

11. M. Harari, E. Williams, S. Castro, K. Brant, Self-leadership: A meta-analysis of over two decades of research. *Journal of Occupational and Organizational Psychology* **94**, 1–34 (2021).

12. K. C. Stanek, D. S. Ones, Meta-analytic relations between personality and cognitive ability. *Proc. Natl. Acad. Sci. U.S.A.* **120**, e2212794120 (2023).

13. S. Highhouse, Y. Wang, D. C. Zhang, Is risk propensity unique from the Big Five factors of personality? A meta-analytic investigation. *Journal of Research in Personality* **98**, 104206 (2022).

14. D. McEwan, P. Boudreau, T. Curran, R. E. Rhodes, Personality traits of high-risk sport participants: A meta-analysis. *Journal of Research in Personality* **79**, 83–93 (2019).

15. I. Thielmann, G. Spadaro, D. Balliet, Personality and prosocial behavior: A theoretical framework and meta-analysis. *Psychological Bulletin* **146**, 30–90 (2020).

16. S. Sharma, W. P. Bottom, H. A. Elfenbein, On the role of personality, cognitive ability, and emotional intelligence in predicting negotiation outcomes: A meta-analysis. *SSRN Journal* **3**, 293–336 (2013).

17. A. Woodcock, W. Graziano, S. Branch, I. Ngambeki, D. Evangelou, Person and thing orientations: Psychological correlates and predictive utility. *Social Psychological and Personality Science* **4**, 117–124 (2013).

18. C. Sibley, J. Duckitt, Personality and prejudice: A meta-analysis and theoretical review. *Pers. Soc. Psychol. Rev.* **12**, 248–79 (2008).

19. U. Barańczuk, The Five-Factor Model of personality and generalized self efficacy: A meta-analysis. *Journal of Individual Differences* **42**, 183–193 (2021).

20. X. Lin, Y. Luan, K. Zhao, T. Zhao, G. Zhao, The antecedents and outcomes of career optimism: A meta-analysis. *CDI* **27**, 409–432 (2022).

21. M. C. Howard, J. E. Cogswell, The "other" relationships of self-assessed intelligence: A meta-analysis. *Journal of Research in Personality* **77**, 31–46 (2018).

22. J. Linardon, Z. McClure, T. L. Tylka, M. Fuller-Tyszkiewicz, Body appreciation and its psychological correlates: A systematic review and meta-analysis. *Body Image* **42**, 287–296 (2022).

23. J. Anglim, S. Horwood, L. D. Smillie, R. J. Marrero, J. K. Wood, Predicting psychological and subjective well-being from personality: A meta-analysis. *Psychological Bulletin* **146**, 279–323 (2020).

24. S. Buecker, M. Maes, J. J. A. Denissen, M. Luhmann, Loneliness and the Big Five personality traits: A meta–analysis. *Eur J Pers* **34**, 8–28 (2020).

25. U. Barańczuk, The Five-Factor Model of personality and emotion regulation: A meta-analysis. *Personality and Individual Differences* **139**, 217–227 (2019).

26. J. Luo, B. Zhang, M. Cao, B. W. Roberts, The stressful personality: A meta-analytical review of the relation between personality and stress. *Pers Soc Psychol Rev* **27**, 128–194 (2023).

27. A. Oshio, K. Taku, M. Hirano, G. Saeed, Resilience and Big Five personality traits: A meta-analysis. *Personality and Individual Differences* **127**, 54–60 (2018).

Table SI8. Level II categorization resolutions for power and sex/gender Level I constructs without consensus

Level I Construct Label	Independent Judges' Level II Categorizations			Level II Construct Final	Resolution Reason	Reference
Academic dishonesty	GAD	RSK	RSK	RSK	"In sum, we showed that people with higher risk-taking propensities tend to behave more dishonestly…To the best of our knowledge, this is the first study to investigate the relationship between risk-taking propensity and cheating using a behavioral approach." (Xu et al., 2019, p. 567)	Z. X. Xu, Y. Wang, M. Zhu, H. K. Ma, Is risk-taking propensity associated with unethical behaviors? An experimental study. *Ethics Behav*. **29**, 557-571 (2019).
Affiliative speech	SEN	SOC	SOC	SOC	The authors' definition of "affiliative language" indicates its function in maintaining relationships, "Affiliative language was defined as language affirming the speaker's relationship with the listener, including statements of support, active understanding, agreement, and acknowledgment." (Park et al., 2016, p. 2)	Park, G. et al. Women are warmer but no less assertive than men: Gender and language on Facebook. *PLOS ONE*. 1-26 (2016).
Aggression	DOM	SEN	DOM	DOM	Archer (2019) placed aggression within a category "Aggression, violence, and dominance" (Table 3, p. 1386)	J. Archer, The reality and evolutionary significance of human psychological sex differences. *Biol. Rev.* **94**, 1381-1415 (2019).
Assertive speech	DOM	GAD	DOM	DOM	"As proposed, men's dominant status in society and their traditional task orientation are enacted through their use of self-assertive language strategies such as directive and instrumental speech." (Leaper & Ayres, 2007, p. 329)	C. Leaper, M. M. Ayres, A meta-analytic review of gender variations in adults' language use: Talkativeness, affiliative speech, and assertive speech. *Pers. Soc.*

						Psychol. Rev. **11**, 328-363 (2007).
Assertiveness	DOM	GAD	DOM	DOM	"A close examination of the literature reveals extensive crossover between dominance and assertiveness." (Williams & Tiedens, 2016, p. 166)	M. J. Williams, L. Z. Tiedens, The subtle suspenson of backlash: A meta-analysis of penalties for women's implicit and explicit dominance behavior. *Psychol. Bull.* **142**, 165-197 (2016).
Auditory selective attention	OTH	GAD	GAD	GAD	"Attention can be regarded as one of the main 'battlefields' of self-regulation, as stimulus-driven influences and goal-directed processing often compete for limited attentional resources." (Hofmann et al., 2012, p. 175)	W. Hofmann, B. J. Schmeichel, A. D. Baddeley, Executive functions and self-regulation. *Trends. Cogn.* **16**, 174-180 (2012).
Competitiveness	GAD	GAD	DOM	DOM	Archer (2019) placed competitiveness within a category "Aggression, violence, and dominance" (Table 3, p. 1386)	J. Archer, The reality and evolutionary significance of human psychological sex differences. *Biol. Rev.* **94**, 1381-1415 (2019).
Cooperativeness	GAD	SEN	SEN	SEN	"Communion’ manifests itself in empathy and understanding, in cooperation and caring for others" (Abele et al., 2008, p. 1204)	A. E. Abele, M. Uchronski, C. Suitner, B. Wojciszke, Towards an operationalization of the fundamental dimensions of agency and communion: Trait content ratings in five countries considering valence and frequency of word occurrence. *Eur. J. Soc. Psychol*. **38**, 1202-1217 (2008).
Desire for status	DOM	GAD	DOM	DOM	Desmichel & Rucker (in press) argue that conspicuous consumption is more	P. Desmichel, D. D. Rucker, Dominance versus prestige

					important in dominance than prestige hierarchies	hierarchies: How social hierarchy base shapes conspicuous consumption. *J. Consum. Res*. (in press).
Disgust	OTH	SEN	OTH	OTH	No clear resolution. Majority coded it as Other.	
Dream recall	ABS	OTH	OTH	OTH	Although there might be some connection between dream recall and creativity and some kinds of abstract associations (Fitch & Armitage, 1989), it cannot be easily and confidently classified.	T. Fitch, R. Armitage, Variations in cognitive style among high and low frequency dream recallers. *Pers. Individ. Differ.* **10**, 869-875 (1989).
Embarrassment	OTH	POS	SEN	SEN	An embarrassment-related experience, such as blushing, "is most commonly caused by undesirable social attention." (Keltner & Anderson, 2000, p. 191). Furthermore, “Embarrassment leads to increased forgiveness, trust, and liking." (Keltner & Anderson, 2000, p. 191)	D. Keltner, C. Anderson, Saving face for Darwin: The functions and uses of embarrassment. *Curr. Dir. Psychol*. **9**, 187-192 (2000).
Engineering interests	OTH	SPA	OTH	OTH	Though spatial abilities seem to be linked to engineering (Wai et al., 2009), interest in engineering is not close enough to a measure of spatial ability.	J. Wai, D. Lubinski, C. P. Benbow, Spatial ability for STEM domains: Aligning over 50 years of cumulative psychological knowledge solidifies its importance. *J. Educ. Psychol.* **101**, 817–835 (2009).
Entitlement	POS	DOM	DOM	DOM	Although it does involve a positive self-evaluation, entitlement is more than that, connected to selfishness and aggression (which are under Dominance) “we demonstrated that individuals who are high in entitlement and involved in romantic relationships	W. K. Campbell, A. M. Bonacci, J. Shelton, J. J. Exline, B. J. Bushman, Psychological entitlement: Interpersonal consequences and validation of a self-report

					display a pattern of selfishness on a range of variables including more dismissing attachment, less overall accommodation, less empathy, perspective taking and respect, greater game playing, and less selflessness. Finally, in Study 9, we demonstrated that PES was linked positively to aggression" (Campbell et al., 2004, p. 42)	measure. *J. Pers. Assess.* **83**, 29-45 (2004).
Episodic memory	SPA	OTH	OTH	OTH	Herlitz and Rehnman (2008) discuss how only some episodic memory is visuospatial, so it is superordinate to spatial ability/performance.	A.Herlitz, J. Rehnman, Sex differences in episodic memory. *Curr. Dir. Psychol.* **17**, 52-56 (2008).
Exercise Motivation	GAD	WEL	OTH	OTH	No clear resolution. Move to Other.	
Face recognition	SEN	SPA	SEN	SEN	Archer (2019) placed face recognition within the category "Social relations" (Table 3, pp. 1386-1387) which is closer to Interpersonal Sensitivity than to Spatial Ability/Performance	J. Archer, The reality and evolutionary significance of human psychological sex differences. *Biol. Rev.* **94**, 1381-1415 (2019).
Fear	POS	WEL	WEL	WEL	Our definition of fear, "feeling of being unable to adjust to harm from specific stimulus/situation should it occur," is closely tied well-being, specifically, to the extent to which one experiences "high environmental mastery"	
Forcing conflict resolution style	GAD	DOM	DOM	DOM	Also known as "dominating conflict style", which involves trying to directly influence others (Tehrani & Yamini, 2020)	H. D. Tehrani, S. Yamini, Personality traits and conflict resolution styles: A meta-analysis. *Pers. Individ. Differ*. **157**, 1-10 (2020).

Forgiveness	SOC	SEN	DOM	DOM	Archer (2019) placed forgiveness within a category "Aggression, violence, and dominance" (Table 3, p. 1386)	J. Archer, The reality and evolutionary significance of human psychological sex differences. *Biol. Rev.* **94**, 1381-1415 (2019).
Giving social support	SEN	SOC	SEN	SEN	Giving social support is related to being able to understand and read individuals' needs, "the analysis suggests that young people are thoughtful, careful and sensitive in the way that they engage with online support. Participants were highly aware of their own privacy and emotional safety needs as well as those of others. They described sensitive and nuanced responses to distress, demonstrating a high level of concern for those with whom they engaged online." (Gibson & Trnka, 2020, p. 245)	K. Gibson, S. Trnka, Young people's priorities for support on social media: "It takes trust to talk about these issues". *Comput. Hum. Behav.* **102**, 238-247 (2020).
Gratitude	SEN	SOC	SOC	SOC	Gratitude can maintain relationships, "the momentary psychological impact from hearing an expression of gratitude forecasted change in the target's relationship satisfaction over 6 months." (Algoe et al., 2013, p. 608)	S. B. Algoe, B. L. Fredrickson, S. L. Gable, The social functions of the emotion of gratitude via expression. *Emotion* **13**, 605-609 (2013).
Guilt	POS	WEL	SEN	SEN	"Both shame and guilt involve affective reactions to evaluations by other people and external (although possibly internalized) standards, and so both imply some form of social sensitivity." (Leith & Baumeister, 1998, p. 2)	K. P. Leith, R. F. Baumeister, Empathy, shame, guilt, and narratives of interpersonal conflicts: Guilt-prone people are better at perspective taking. *J. Pers. Soc. Psychol.* **66**, 1-37 (1998).

Hubristic pride	POS	DOM	DOM	DOM	Grijalva & Zhang (2016) categorize "arrogant" under Agency (Table 1, p. 8), and arrogance is a feature of hubristic pride (Tracy, Mercadente, & Hohm, 2023)	E. Grijalva, L. Zhang, Narcissism and self-insight: A review and meta-analysis of narissists' self-enhancement tendencies. *Pers. Soc. Psychol. Bull.* **42**, 3-24 (2015).<br><br>J. L. Tracy, E. Mercadante, I. Hohm, Pride: The emotional foundation of social rank attainment. *Annu. Rev. Psychol.* **74**, 519-545 (2023).
Impulsivity	GAD	GAD	RSK	GAD	No clear resolution. Majority coded it as Goal Approach & Disinhibition	
Inequity aversion	PRD	SEN	SEN	OTH	These are cases of inequity aversion that contain strong components of both self- and other-interest, so there is no clear resolution between Agency and Communion. Move to Other.	
Interpersonal counterproductive work behavior	SEN	SEN	DOM	DOM	A large portion of the effects in Ng et al. (2016) involve aggression, abusive supervision, and incivility. These include, or are similar to, other constructs in Dominance.	T. W. Ng, S. S. Lam, D. C. Feldman, Organizational citizenship behavior and counterproductive work behavior: Do males and females differ? *J. Vocat. Behav*. **93**, 11-32 (2016).
Interpersonal organizational citizenship behavior	SOC	SEN	SEN	SEN	This is helping behavior within organizations, and helping behavior was coded Interpersonal Sensitivity by all coders.	
Interpersonal orientation	SOC	SEN	SOC	SOC	Our definition of interpersonal orientation involves "concern with relational dynamics," which is important for maintaining social	

					connections, implicit our definition of sociability, "extent to which one engages in social interactions, forms and maintains social connections, and participates in social activities."	
Interrupting	SEN	DOM	DOM	DOM	"Paraverbal behaviors associated with dominance include ... interruptions (Street & Buller, 1987)." (Williams & Tiedens, 2016, p. 166)	M. J. Williams, L. Z. Tiedens, The subtle suspenson of backlash: A meta-analysis of penalties for women's implicit and explicit dominance behavior. *Psychol. Bull.* **142**, 165-197 (2016).
Justice-based moral judgement	GAD	SEN	OTH	OTH	No clear resolution. Majority coded it as Other.	
Leadership effectiveness	OTH	GAD	OTH	OTH	"The present results replicate the findings of earlier studies: Transformational leadership added to the prediction of subordinates' ratings of leader effectiveness and satisfaction beyond that of transactional leadership" (Hater & Bass, 1988, p. 700). Transformational leadership includes individualized consideration, which is communal, whereas transactional leadership is more agentic. Thus, it involves high agency and high communion, which cannot be classified.	J. J. Hater, B. M. Bass, Superiors' evaluations and subordinates' perceptions of transformational and transactional leadership. *J. Appl. Psychol*. **73**, 695 (1988).
Loneliness	POS	SOC	WEL	WEL	The discrepancy between "the desired and actual social relationships in terms of companionship, connectedness, or intimacy," in our definition of loneliness is closely tied to one's well-being, specifically the "extent to which one	

					experiences high life satisfaction, high ratio of positive to negative affect, positive relations..."	
Loss aversion	GAD	RSK	RSK	RSK	"The test of loss aversion was straightforward. The translation of all outcomes into the positive domain eliminated the experience of loss, which we consider the most important factor in inducing risk aversion." (Thaler et al., 1997, p. 657)	R. H. Thaler, A. Tversky, D. Kahneman, A. Schwarz, The effect of myopia and loss aversion on risk taking: An experimental test. *Q. J. Econ.* **112**, 647-771 (1997).
Lying	GAD	RSK	RSK	RSK	"Ethical risk taking pertains to behaviors (e.g., cheating, lying, and illegal activity) that are generally judged to be immoral." (Sevi & Shook, 2021, p. 964)	B. Sevi, N. J. Shook, The relation between disgust sensitivity and risk-taking propensity: A domain specific approach. *Judgm. Decis. Mak.* **16**, 950-968 (2021).
Meta-stereotyping	PRD	PRD	OTH	PRD	"Another seemingly counterintuitive predictor of metastereotyping is prejudice; to the extent that individuals are themselves prejudiced toward outgroups, they likely believe others are similarly prejudiced toward their own group (see Vorauer & Kumhyr, 2001)." (Finkelstein et al., 2015, p. 27)	L. M. Finkelstein, E. B. King, E. C. Voyles, Age metastereotyping and cross-age workplace interactions: A meta view of age stereotypes at work. *Work Aging Retire*. **1**, 26-40 (2015).
Moral condemnation	OTH	SEN	OTH	OTH	No clear resolution. Majority coded it as Other.	
Moral hypocrisy	OTH	SEN	OTH	OTH	No clear resolution. Majority coded it as Other.	
Morningness-eveningness	OTH	OTH	OTH	OTH		
Narcissism	SEN	DOM	DOM	DOM	"Narcissism was located as a vector cutting between the high-agency and low-communion axes" (Paulhus, 2001, p. 228) Also, "This lends support to the	G. Seidman, P. E. Shrout, V. Zeigler-Hill, Untangling the associations that narcissistic admiration and narcissistic

					notion that narcissism may exist in both high agency/low communion and high agency only forms" (Seidman et al., 2020, p. 11)	rivalry have with agency, communion, and romantic commitment. *J Res Pers*. **89**, 507-512 (2020).
Negotiation performance	ABS	GAD	OTH	OTH	Assertive behavior helps achieve better outcomes, but so does interpersonal sensitivity (Schweinsberg et al., 2022). Theory and evidence point to taking either agency or communion too far is not as productive as balancing the two (Amanatullah et al., 2008). Since it is both agency and communion, move to Other.	M. Schweinsberg, S. Thau, M. M. Pillutla, Negotiation impasses: Types, causes, and resolutions. *J. Manag.* **48**, 49-76 (2022).<br><br>E. T. Amanatullah, M. W., Morris, J. R. Curhan,. Negotiators who give too much: Unmitigated communion, relational anxieties, and economic costs in distributive and integrative bargaining. *J. Pers. Soc. Psychol.* **95**, 723-738 (2008).
Network brokerage	OTH	SOC	OTH	OTH	No clear resolution. Majority coded it as Other.	
Nightmare frequency	OTH	WEL	OTH	WEL	"Nightmare frequency had more significant correlations with the measures of psychological well-being than did bad-dream frequency." (Zadra & Donderi, 2000, p. 273)	A. Zadra, D. C. Donderi, Nightmares and bad dreams: their prevalence and relationship to well-being. *J. Abnorm. Psychol*. **109**, 273-281 (2000).
Norm-based (vs consequence-based) moral judgement	OTH	SEN	SEN	SEN	Norm-based judgment requires inferring what other people think is right vs wrong. "A third family of models [norm-based] proposes that people judge an action wrong by considering how many other people judge it wrong—that is, whether there	S. Levine, M. Kleiman-Weiner, L. Schulz, J. Tenenbaum, F. Cushman, The logic of universalization guides moral judgment. *Proc. Natl. Acad. Sci. U.S.A.* **117**, 26158-26169 (2020).

					is a norm against it." (Levine et al., 2020, p. 26160)	
Objectification	GAD	DOM	GAD	GAD	"The process of objectification is thought to involve a kind of instrumental fragmentation in social perception, the splitting of a whole person into parts that serve specific goals and functions for the observer." (Gruenfeld et al., 2008, p. 111)	D. H. Gruenfeld, M. E. Inesi, J. C. Magee, A. D. Galinsky, Power and the objectification of social targets. *J. Pers. Soc. Psychol*. **95**, 111-127 (2008).
Organizational counterproductive work behavior	OTH	DOM	OTH	OTH	No clear resolution. Majority coded it as Other.	
Pain threshold	OTH	GAD	OTH	OTH	No clear resolution. Majority coded it as Other.	
Pain tolerance	OTH	GAD	OTH	OTH	No clear resolution. Majority coded it as Other.	
Peer attachment	SEN	SOC	SOC	SOC	"Therefore, findings obtained from self-report measures, such as the IPPA, supported theoretical assumptions of the attachment theory, showing that not only do secure individuals demonstrate an ability to tolerate negative affect while maintaining constructive engagement with others, but they also are able to display positive emotions that enhance social interaction and social competence." (Gorrese & Ruggieri, 2012, p. 651)	A. Gorrese, R. Ruggieri, R, Peer attachment: A meta-analytic review of gender and age differences and associations with parent attachment. *J. Youth Adolesc*. **41**, 650-672 (2012).
Perceived responsibility	WEL	DOM	OTH	OTH	No clear resolution. Majority coded it as Other.	
Perception of own (vs. others') size	POS	POS	DOM	DOM	"Agency and body esteem weight were the only significant predictors of self-reported height in men." (Pozzebon et al., 2012) p. 2692 and "Dominance	J. A. Pozzebon, B. A. Visser, A. F. Bogaert, Do you think you're sexy, tall, and thin? The prediction of self-rated

					arises relationally and is not an individual-level trait, but is often correlated to individual traits such as resource-holding potential and physical size." (Zeng et al., 2022, p. 2)	attractiveness, height, and weight. *J. Appl. Soc. Psychol*. **42**, 2671-2700 (2012).<br><br>T. Chen Zeng, J. T. Cheng, J. Henrich, Dominance in humans. *Philos. Trans. R. Soc*. **377**, 1-12 (2022).
Updating (executive function)	GAD	ABS	GAD	GAD	"Operation span (see Box 1) and n-back measures, for instance, have been found to reflect primarily the updating function (working memory), consistent with the high task demands of maintaining and updating task-relevant information." (Hofmann et al., 2012, p. 174), and Table 1 (p. 175) discusses the importance of working memory (updating) for self-regulation of goal pursuit.	W. Hofmann, B. J. Schmeichel, A. D. Baddeley, Executive functions and self-regulation. *Trends. Cogn.* **16**, 174-180 (2012).
Person orientation	SOC	SEN	SOC	SOC	Several items in Graziano et al. (2011) represent the core of person-orientation, which include behaviors related to sociability, i.e., "Strike up a conversation with a homeless person on a street," "Listen with caring interest to an old person who sits next to you on a bus," "Make the first attempt to meet a new neighbor." (Table 1, p. 30)	W. G. Graziano, M. M. Habashi, A. Woodcock, Exploring and measuring differences in person-thing orientations. *Pers. Individ. Differ*. **51**, 28-33 (2011).
Personal space	OTH	SOC	DOM	SOC	"Participants then provided an implicit behavioral measure of affiliative motivation (how closely participants sat to an anticipated social partner)" (Case et al., 2015, p. 380)	C. R. Case, K. E. Conlon, J. K. Maner, Affiliation-seeking among the powerless: Lacking power increases social affiliative motivation.

						Eur. J. Soc. Psychol. **45**, 378-385 (2015).
Preference for choice	GAD	RSK	ABS	OTH		
Preference for feasibility (vs desirability)	GAD	RSK	ABS	ABS	Construal Level Theory predicts feasibility considerations are more likely to "guide near-future preferences," whereas desirability considerations are more likely to "guide distant-future preferences." (Trope & Liberman, 2003, p. 410). Along this line of reasoning, they identify the "near future" as being "construed in terms of more complex, concrete representations," whereas the "distant future" is "construed in terms of relatively simple, abstract representations." (Trope & Liberman, 2003, p. 412)	Y. Trope, N. Liberman, Temporal construal. *Psychol. Rev*. **110**, 403-421 (2003).
Prejudice	SEN	PRD	PRD	PRD	Our definition of prejudice and dehumanization includes, "extent to which one has strong negative attitudes toward social groups/categories, stereotypes outgroup members, and/or neglects others' attributes that define their individuality or humanness". "Prejudice" is an anchor construct of the category, "Prejudice and dehumanization"	
Punishment sensitivity	RSK	GAD	GAD	GAD	“The BIS, according to Gray [1990], is sensitive to signals of punishment, nonreward, and novelty. It inhibits behavior that may lead to negative or painful outcomes.” (Carver & White, 1994, p. 319)	C. S. Carver, T. L. White, Behavioral inhibition, behavioral activation, and affective responses to impending reward and punishment: The BIS/BAS

						scales. *J. Pers. Soc. Psychol.* ***67***, 319–333 (1994).
Reaction time	OTH	GAD	GAD	GAD	"Lack of agency: Slow, aimless" (Abele et al., 2008, p. 1204)	A. E. Abele, M. Uchronski, C. Suiter, B. Wojciszke, Towards an operationalization of the fundamental dimensions of agency and communion: Trait content ratings in five countries considering valence and frequency of word occurrence. *Eur. J. Soc. Psychol.* **38**, 1202-1217 (2008).
Resistance to temptation	GAD	RSK	GAD	RSK	These are all unethical behavior situations. The measures are almost all academic dishonesty, one stealing, and one keeping more than deserved. Other dishonesty variables coded as Risk-seeking.	
Response conflict (executive function)	GAD	GAD	GAD	GAD	"Inhibition is typically assessed with versions of the Stroop [14] or stop-signal task [4] in which participants have to inhibit or override a prepotent response." (Hofmann et al., 2012, p. 174), and Table 1 (p. 175) discusses the importance of behavioral (response) inhibition for self-regulation of goal pursuit.	W. Hofmann, B. J. Schmeichel, A. D. Baddeley, Executive functions and self-regulation. *Trends. Cogn.* **16**, 174-180 (2012).
Scholastic achievement	OTH	GAD	GAD	OTH	No clear resolution. Move to Other	
Self-compassion	POS	WEL	WEL	WEL	"Self-compassion appears to foster well-being among all people, regardless of gender, age, or culture (e.g., Akin, 2010; Allen & Leary, 2014; Arimitsu,	L. M. Yarnell, R. E. Stafford, K. D. Neff, E. D. Reilly, M. C. Knox, M. Mullarkey, Meta-analysis of gender differences

					2014; Choi, Lee, & Lee, 2014; Neff et al., 2008)." (Yarnell et al., 2015, p. 501)	in self-compassion. *Self Identity* **14**, 499-520 (2015).
Self-esteem	WEL	POS	POS	POS	"It appears, then, that those who are highly certain of their positive self-views and who also consider these views especially important are especially likely to enjoy the benefits of high self-esteem." (Pelham & Swann, 1989, p. 676)	B. W. Pelham, W. B. Swann, From self-conceptions to self-worth: On the sources and structure of global self-esteem. *J. Pers. Soc. Psychol.* **57**, 672-680 (1989).
Selfishness	GAD	DOM	DOM	DOM	Our definition of dominance includes "assertive and direct expression of one's own needs, opinions, and desires", which is reflected in the construct selfishness.	
Sense of personal control	POS	WEL	POS	POS	The measures from these studies are less about autonomy (which could be connected to well-being) and more about effectance (which is closer to a positive self-evaluation): "…the "impact" framework, suggests that perceived control depends on how much people believe that important outcomes are contingent upon, rather than independent of, their behavior (Rotter, 1966)." (Brockner et al., 2004, p. 78)	J. Brockner, G. Spreitzer, A. Mishra, W. Hochwarter, L. Pepper, J. Weinberg, Perceived control as an antidote to the negative effects of layoffs on survivors' organizational commitment and job performance. *Adm. Sci. Q.* **49**, 76-100 (2004).
Shame	POS	WEL	POS	POS	Our definition of shame refers to "feeling of self being negatively evaluated; self-conscious emotion," which would indicate low levels of positive views of self, i.e., "extent to which one has positive perceptions and/or evaluations globally of the self or of specific attributes of the self"	

Smiling	SOC	WEL	SOC	SOC	There exist gender-based norms for smiling, which involve the appropriateness of smiling as a social behavior, "Ekman and Friesen (1975) coined the term display rules to refer to the set of guidelines learned by individuals through socialization that dictate the socially appropriate management of facial expression. Display rules might specify that a particular facial expression be intensified, deintensified, neutralized, or masked with another emotion." (LaFrance et al., 2003, p. 307)	M. LaFrance, M. A. Hecht, E. L. Paluck, The contingent smile: A meta-analysis of sex differences in smiling. *Psychol. Bull.* **129**, 305-334 (2003).
Social dominance orientation	DOM	PRD	PRD	PRD	"For both analyses considered, SDO measured in 1996 significantly predicted prejudice and discrimination in 2000, net of levels of these same constructs in 1996." (Kteily et al., 2011, p. 213)	N. S. Kteily, J. Sidanius, S. Levin, Social dominance orientation: Cause or 'mere effect'? evidence for SDO as a causal predictor of prejudice and discrimination against ethnic and racial outgroups. *J. Exp. Soc. Psychol.* **47**, 208-214 (2011).
Social vocational interests	SOC	SEN	SEN	SEN	Includes elements of both interpersonal sensitivity and sociability. Preference for environments that: encourage people to see themselves as liking to help others, understanding others, cooperative, and reward people for the display of social values - these would be interpersonal sensitivity; stimulate people to engage in social activities and foster social competencies and	J. L. Holland, *Making vocational choices: A theory of careers.* (Englewood Cliffs, NJ: Prentice-Hall, 1973).

					encourage people to see themselves as sociable - these would be sociability. Could go under either category since both are under communion; chose interpersonal sensitivity based on majority of coders.	
Source attribution accuracy	SEN	OTH	OTH	OTH	No clear resolution. Majority coded it as Other.	
Susceptibility to influence	OTH	DOM	DOM	DOM	Our definition of dominance includes "extent to which one takes control and attempts to influence others, including simply assertive and direct expression of one's own needs, opinions, and desires"	
Systemizing quotient	SPA	ABS	OTH	OTH	No clear resolution. Majority coded it as Other.	
Talkativeness	SOC	SOC	DOM	DOM	"Paraverbal behaviors associated with dominance include talk time (Cashdan, 1998; Dovidio, Brown, et al., 1988; Kalma, 1991; Kimble & Musgrove, 1988; Leaper & Ayres, 2007; Van de Sande, 1980)" (Williams & Tiedens, 2016, p. 166)	M. J. Williams, L. Z. Tiedens, The subtle suspenson of backlash: A meta-analysis of penalties for women's implicit and explicit dominance behavior. *Psychol. Bull.* **142**, 165-197 (2016).
Task-oriented leadership	ABS	GAD	GAD	GAD	Grijalva & Zhang (2015) list Leadership and Task-performance as agentic and define them around goal completion. (Table 1, p. 8)	E. Grijalva, L. Zhang, Narcissism and self-insight: A review and meta-analysis of narcissists' self-enhancement tendencies. *Pers. Soc. Psychol. Bull.* **42**, 3-24 (2015).
Tentative speech	OTH	DOM	DOM	DOM	"Paraverbal behaviors associated with dominance include ... a lack of hesitations in speech (Fragale, 2006; Norton-Ford & Hogan, 1980)" (Williams & Tiedens, 2016, p. 166)	M. J. Williams, L. Z. Tiedens, The subtle suspenson of backlash: A meta-analysis of penalties for women's implicit and explicit

						dominance behavior. *Psychol. Bull.* **142**, 165-197 (2016).
Thing orientation	OTH	SPA	OTH	OTH	No clear resolution. Majority coded it as Other.	
Touch initiation	OTH	GAD	DOM	OTH	No clear resolution. Majority coded it as Other.	
Trust in others	SEN	SEN	SOC	SEN	No clear resolution, but both interpersonal sensitivity and sociability are under Communion. Majority coded it as Interpersonal Sensitivity.	
Verbal fluency	CRE	CRE	OTH	OTH	Verbal fluency is a measure of verbal, not creative, ability: "The two verbal abilities, however, that textbooks and review articles typically refer to when claiming the existence of a female advantage are verbal fluency (sometimes also called "word fluency") and verbal memory (Andreano & Cahill, 2009; Halpern, 2012; Hamson et al., 2016; Hyde, 2014; Kimura, 2000; Miller & Halpern, 2014)." (Hirnstein et al., 2023, p. 67).)	M. Hirnstein, J. Stuebs, A. Moè, M. Hausmann, Sex/gender differences in verbal fluency and verbal-episodic memory: a meta-analysis. *Perspect. Psychol. Sci*. **18**, 67-90 (2023).
Verbal working memory	SPA	ABS	OTH	OTH	Voyer et al. (2021) is the source for this measure, which they distinguish from a visuospatial working memory measure from another paper. This verbal working memory measure is not clearly connected with spatial ability or abstraction.	D. Voyer, J. Saint Aubin, K. Altman, G. Gallant, Sex differences in verbal working memory: A systematic review and meta-analysis. *Psychol. Bull.* **147**, 352 (2021).
Verbal-episodic memory	SPA	OTH	OTH	OTH	Hirnstein et al. (2023) deliberately excluded visuospatial episodic memory from their meta-analysis of two verbal	M. Hirnstein, J. Stuebs, A. Moè, M. Hausmann, Sex/gender differences in verbal fluency and verbal-

					abilities. Verbal-episodic memory is a verbal, not spatial, ability.	episodic memory: a meta-analysis. *Perspectives on Psychological Science*. **18**, 67-90 (2023).
Vividness of visual imagery	SPA	SPA	OTH	OTH	No clear resolution. Move to Other	
Working memory	OTH	ABS	GAD	OTH	Working memory is too broad to fit into any one category. "The term working memory refers to a brain system that provides temporary storage and manipulation of the information necessary for such complex cognitive tasks as language comprehension, learning, and reasoning." (Baddeley, 1992 from the Abstract)	A. Baddeley, Working memory. *Science* **255**, 556-559 (1992).

Note. Includes Level I power and sex/gender constructs for which there was no consensus among judges. Resolutions were determined by consulting literature on the topic and our construct definitions. DOM = Dominance; GAD = Goal Approach & Disinhibition; RSK = Risk-seeking; SEN = Interpersonal Sensitivity; SOC = Sociability; PRD = Prejudice & Dehumanization; POS = Positive Views of the Self; WEL = Well-being; SPA = Spatial Ability/Performance; CRE = Creative Performance; ABS = Abstract Cognition; OTH = Other. Where Level II Construct Final = OTH, construct was excluded from further analysis.

Table SI9. Level II categorization resolutions for extraversion Level I constructs without consensus

**Level I Construct Label**	**Definition Used by Judges**	**Independent Judges' Level II Categorizations**			**Level II Construct Final**
Academic satisfaction	Satisfaction with one's academic experience	POS	WEL	WEL	WEL
Antisocial behavior	Criminal and antisocial behavior (e.g., stealing, stalking, bullying), delinquency, and conduct disorder	PRD	OTH	RSK	OTH
Career adaptability	Preparation for, control over, curiosity about, and self-efficacy about one's career opportunities	POS	GAD	GAD	GAD
Career satisfaction	Satisfaction with one's career	POS	WEL	WEL	WEL
Change-oriented behavior	Behaviors that enhance effectiveness based on initiative and changes to the work situation	OTH	GAD	GAD	GAD
Conservation value: conformity	Value behavioral restraint likely to upset or harm others and violate social expectations	OTH	SEN	SEN	SEN
Contextual performance	Discretionary behaviors that in aggregate, promote effective organizational functioning	GAD	OTH	GAD	GAD
Counterproductive work behavior: cyberloafing	Using technology to idle instead of work	GAD	OTH	OTH	OTH
Emotional expressiveness	Nonverbal behaviors that involve expressing and/or encoding emotions	SEN	OTH	SEN	SEN
Entrepreneurial intentions	Expressed behavioral intention to become an entrepreneur	GAD	OTH	OTH	OTH
Job search success: job search intensity	Frequency and scope of job search behaviors, and resources devoted toward that search	OTH	GAD	GAD	GAD
Organizational citizenship behavior: change	Behaviors intended to enhance the organization by bringing about proactive and positive changes	OTH	GAD	GAD	GAD

Performance rating leniency	Self-reported measures of leniency in performance ratings	GAD	OTH	OTH	OTH
Resilience	Capacity, processes, and/or outcomes of successful adaptation in the context of significant threats to functioning or development	OTH	WEL	GAD	OTH
Roles in expressive social networks: indegree	Number of ties received from others in socio-emotional social networks	SOC	OTH	SOC	SOC
Roles in instrumental social networks: indegree	Number of ties received from others in instrumental social networks	SOC	OTH	OTH	OTH
Self-transcendence value: universalism	Value tolerance, concern, and appreciation for the welfare of humankind and nature	OTH	PRD	OTH	OTH
Study attitudes	Positive attitude toward studying	OTH	GAD	OTH	OTH
Study habits	Regular engagement in sound study behaviors and routines	OTH	GAD	GAD	GAD
Transactional leadership: contingent reward	Behaviors intended to exchange tangible or nontangible support and resources with followers based on their efforts and performance	GAD	OTH	OTH	OTH
Transactional leadership: management by exception	Behaviors involving setting performance standards and monitoring deviations, acting on an as-needed basis	DOM	OTH	OTH	OTH
Transactional leadership: passive leadership	Behaviors involving passive leadership and intervening only when problems become serious	OTH	DOM	OTH	OTH
Transformational leadership: charisma	Followers identify with a leader's vision and leader fosters optimism via action	DOM	OTH	POS	OTH

Transformational leadership: intellectual stimulation	Behaviors exhorting followers to reframe problems, develop novel ideas, or approach issues in new ways	DOM	OTH	OTH	OTH
Work-life balance: family interference with work	Degree to which family role participation interfered with work role responsibilities	GAD	OTH	OTH	OTH
Work-life balance: work-nonwork negative spillover	Degree to which work (nonwork) role participation is worsened by participating in the other role	GAD	OTH	OTH	OTH
Work-life balance: work-nonwork positive spillover	Degree to which work (nonwork) role participation is improved by participating in the other role	GAD	OTH	OTH	OTH

Note. Includes only Level I constructs for which there was no consensus among judges. Resolutions were determined by majority categorization; otherwise (i.e., all three judges differed), the construct was deemed uncategorizable (i.e., "Other"). DOM = Dominance; GAD = Goal Approach & Disinhibition; RSK = Risk-seeking; SEN = Interpersonal Sensitivity; SOC = Sociability; PRD = Prejudice & Dehumanization; POS = Positive Views of the Self; WEL = Well-being; SPA = Spatial Ability/Performance; CRE = Creative Performance; ABS = Abstract Cognition; OTH = Other. Where Level II Construct Final = OTH, constructs was excluded from further analysis.

Table SI10. Complete list of Level I and Level II constructs and definitions

**Level I Construct**	**Definition**
absenteeism[a]	absence from or lateness to work
abstract reasoning	progressive matrices, a non-verbal test
abstract thinking	cognitions about central and superordinate features of events, situations, and targets
abusive supervision perceptions[a]	perception that supervisor engages in hostile verbal and nonverbal (e.g., physical) behaviors
academic attendance[a]	postsecondary class attendance
academic performance: postsecondary[a]	grades or grade point average
academic procrastination	postponing, delaying, or putting off academic tasks or responsibilities
academic satisfaction	satisfaction with one's academic experience
academic self-efficacy	confidence in own ability to achieve in academic contexts
academic success[a]	grades or grade point average, and attendance
accidents: occupational[a]	safety incidents resulting in worker injury or property damage at work
accidents: vehicular[a]	vehicular accidents or injuries taking place in traffic
achievement learning orientation[a]	aware of implications of academic demands; competitive, confident
action orientation	likelihood of active (vs. passive) response; less deliberation
adaptive performance	incumbent proficiency in altering their performance behavior in response to the demands of a new task, event, situation, or an environmental constraint
adjustment to college: academic[a]	degree to which one has adapted to the academic demands of college
adjustment to college: institutional attachment[a]	degree to which one identifies with and has become emotionally attached to university community
adjustment to college: overall[a]	degree to which one has adapted to college in general
adjustment to college: personal-emotional	degree to which one experiences stress, anxiety, and/or physical reactions to college demands
adjustment to college: social	degree to which one has integrated themselves into the social environment in college
affiliation motivation	capacity for deriving pleasure from being with others and experiencing social separation as aversive
affiliative speech	speech that affirms or positively engages others
aggression	attempt to cause harm to another person; can be direct or indirect
anger-related emotions	feelings of displeasure, hostility and desire to aggress

antisocial behavior[a]	criminal and antisocial behavior (e.g., stealing, stalking, bullying), delinquency, and conduct disorder
anxiety-related emotions	diffuse feelings of apprehension, unease and fear, including social anxiety
applicant attraction to organization[a]	attitudinal attraction towards a prospective employer
assertive speech	clear and direct verbal communication
assertiveness	proactive and direct expression of one's own interests and opinions
attitudes to cheating	favorable attitudes toward cheating
auditory selective attention	ability to attend to target or focal sounds while filtering out distracting sounds
authentic pride	positive feeling from a sense of accomplishment or achievement attributable to the self; self-conscious emotion
authenticity	behaving in ways that reflects one's true values, beliefs, and emotions
autocratic leadership style	opposite of democratic leadership style; don't allow subordinates to participate in decision-making
burnout	low energy, negative affect, and/or perceptions of depleted emotional resources due to work stress
caring emotions	affect that reflects nurturing, being drawn to young children and pets, feeling softhearted toward animals and people in need, feeling empathy, liking to care for the sick, feeling affection for and liking to care for others, as well as liking to be needed by others
care-based moral judgment	guided by concern with avoiding harm to individuals and maintaining relationships; typically in contrast to justice-based moral judgment
career adaptability	preparation for, control over, curiosity about, and self-efficacy about one's career opportunities
career decision-making difficulties[a]	cognitive and affective difficulties in making career-related decisions
career optimism	extent to which people believe their future will be prosperous and favorable
career satisfaction	satisfaction with one's career
change-oriented behavior	behaviors that enhance effectiveness based on initiative and changes to the work situation
competitiveness	desire to outperform others and behavior consistent with that goal
confidence	strength of conviction in one's beliefs
conservation value: conformity	value behavioral restraint likely to upset or harm others and violate social expectations
conservation value: security	value personal and social security
conservation value: tradition[a]	value maintaining cultural and religious traditions and customs
contextual performance	discretionary behaviors that in aggregate, promote effective organizational functioning
cooperativeness	desire to increase collective welfare and behaviors that coordinates with others toward that goal
counterproductive work behavior: cyberloafing[a]	using technology to idle instead of work

creativity	product creation, innovation, novel idea generation, divergent thinking, or number of patents; performance at developing something original and potentially useful
dehumanization	seeing and thinking of others in terms that neglect their individuality and humanness
demonstrating effort	hard work, extra effort, and willingness to work long hours under adverse conditions
depth of processing[b]	level of cognitive engagement, ranging from superficial features to deep meaning
desire for power	preference for having influence and control over others
desire for status	preference for conspicuous markers of status (e.g., visibly valuable goods)
disgust[b]	strong negative emotion in response to something that is literally or morally repulsive or contaminating
dream recall[c]	ability to remember dreams
embarrassment	emotion that arises from realization that one has violated a social norm or made a mistake; self-conscious emotion
emotional expressiveness	nonverbal behaviors that involve expressing and/or encoding emotions
emotional intelligence	noncognitive, especially emotion-related, capabilities, competencies and skills that influence one's ability to succeed in coping with environmental demands and pressures
empathic accuracy	ability to accurately recognize non-verbal emotion displays
empathy quotient	drive to identify the thoughts and emotions of others and respond appropriately
employee engagement: overall	attitudes about the investment of energies in the experience or performance of work
engagement in high-risk sports	leisure physical activities where the most likely outcome of a mismanaged mistake or accident is severe injury or death
engineering interests[c]	interest in designing, building, and testing products and systems, and in application of math and science
entitlement	extent to which an one feels deserving of positive outcomes
entrepreneurial intentions[a]	expressed behavioral intention to become an entrepreneur
episodic memory[c]	memory of events in one's experience encoded in a particular temporal-spatial context
excitement-seeking	desire for novel or intense experiences, even if risks are involved
expatriate adjustment: general[a]	degree to which one has adapted to everyday life experiences in the new culture (e.g., food, weather)
expatriate adjustment: interactional[a]	degree to which one has adapted to interacting with host-nationals
expatriate adjustment: overall[a]	degree to which one has adapted to expatriate life overall
expatriate adjustment: work[a]	degree to which one has adapted to foreign work roles
face recognition	ability to perceive and remember faces
fear	feeling of being unable to adjust to harm from specific stimulus/situation should it occur
forcing conflict resolution style	attempting to attain one's goals at the sacrifice of others' goals; selfish, aggressive, and argumentative; same as dominating style

forgiveness	prosocial motivational changes that occur after a person has incurred a transgression
generalized self-efficacy	beliefs about one's ability to deal with a broad variety of challenging or difficult situations
"getting along" performance	showing interpersonal skill, sharing credit, etc
giving social support	offering social-emotional support in person or on social network sites
goal conflict	experiencing incompatible goals
goal orientation: learning	goal orientation to seek opportunities to learn and grow in performance settings
goal orientation: performance avoidance[a]	goal orientation to avoid disapproval and negative judgments about one's competence in performance settings
goal orientation: performance prove	goal orientation to prove one's competence and gain favorable judgments in performance settings
goal pursuit	maintaining cognition and behavior consistent with one's goals; including attention to goal-relevant information, action taken toward a goal, trying different ways to get to the goal, etc.
gratitude	positive emotion showing thankfulness to another person; includes perceptions of the cost of the favor to the giver and perceptions of the value of the favor to the receiver
guilt	feeling that something one has done is negatively evaluated; self-conscious emotion
happiness	feeling joy and satisfaction
helping behavior	voluntary behavior intended to benefit or assist others
hubristic pride	inflated sense of superiority and arrogance; affective component of narcissism; self-conscious emotion
impulsivity	tendency to act spontaneously and without deliberation
inequity aversion[b]	avoidance of inequitable outcomes, even when the self is advantaged
interpersonal citizenship behavior	behavior intended to maintain and enhance the organization, mainly directed at other individuals
interpersonal counterproductive work behavior	intentional employee acts that harm other individuals and/or their productivity (e.g., aggression toward co-workers, using unprofessional language with customers)
interpersonal citizenship behavior	acts performed by employees for the benefit of other individuals
interpersonal orientation	concern with relational dynamics
interrupting	tendency to interrupt others
irresponsible behavior[a]	poor attendance, disciplinary actions, counterproductive behavior, failure to follow direction, absenteeism, or substance use
job complexity[a]	complexity of one's job
job crafting	behaviors intended to actively change the perceived characteristics of one's job
job satisfaction	pleasure or contentment in one's work or job roles

job search self-regulation	attitudes and behaviors directed at exploring and clarifying job search goals, as well as planning and self-regulating actions to implement them
job search success: employment quality[a]	extrinsic factors and subjective attitudes about a new job vis-à-vis a prior position
job search success: employment status[a]	whether a job seeker had found a new job or not after a given period
job search success: job search intensity	frequency and scope of job search behaviors, and resources devoted toward that search
justice-based moral judgment[c]	guided by depersonalized principles of fairness and individualism; typically in contrast to care-based moral judgment
language ability[c]	performance in language-based courses
leader-member exchange[a]	quality of exchange relationship with a manager or supervisor
leadership effectiveness[c]	effectiveness as a leader
life satisfaction	evaluation of one's life overall
line angle judgment	one component of visuospatial ability; ability to accurately perceive and compare orientations of lines and angles
loneliness	subjective perception of a discrepancy between the desired and actual social relationships in terms of companionship, connectedness, or intimacy
loss aversion	greater emphasis on avoiding losses than approaching equivalent gains
lying	one type of unethical behavior; dishonesty that benefits self, others, or both (in experimental games)
mental rotation	cognitive process through which one rotates an image or object in their mind
metastereotyping	beliefs about the stereotypes that members of outgroups hold about the ingroup
moral condemnation[b]	disapproval of others' behavior as immoral
moral hypocrisy[b]	imposition of strict moral standards on others but less strict moral standards on oneself
moral sensitivity	awareness of how one's behavior affects others
morningness-eveningness[c]	degree to which one feels alert and awake in morning (vs evening)
motivation to apologize	drive to express remorse, take responsibility, and seek reconciliation in the aftermath of wrongdoing
narcissism	grandiosity, need for admiration, and lack of empathy
negative affect	unpleasant emotions and moods
negotiation initiation	tendency to start negotiating intentionally and voluntarily
negotiation performance[bc]	outcomes of negotiation
network brokerage[c]	likelihood of being connected to disconnected people
nightmare frequency	frequency of having nightmares

norm-based (vs consequence-based) moral judgment	morality judged based on consistency with norms (deontology) vs what the consequences are (utilitarianism)
object location memory	a component of spatial memory; ability to recall the location of objects in a specific environment
objectification	seeing a person not in terms of their own goals but in terms of how they could satisfy one's own goals
occupational interests[c]	masculine vs feminine occupational preferences
occupational stress	stress from the demands of work
openness-to-change value: self-direction	value autonomy of thought and action
openness-to-change: stimulation	value excitement, novelty, and challenge in life
optimism	tendency to expect positive events and outcomes
change-oriented organizational citizenship behavior	behaviors intended to enhance the organization by bringing about proactive and positive changes
organizational citizenship behavior: global[a]	behaviors intended to maintain and enhance organizational context that supports task performance
organizational citizenship behavior: organizational[a]	behaviors intended to maintain and enhance the organization, mainly directed at the organization
organizational commitment: affective[a]	emotional attachment to an organization
organizational commitment: continuance[a]	evaluation of costs and benefits associated with leaving an organization
organizational commitment: global[a]	overall attachment to an organization
organizational commitment: normative[a]	felt obligation to remain with an organization
organizational counterproductive work behavior[c]	intentional employee acts that undermine the organization and its business interests (e.g., damaging company property, badmouthing the organization to outsiders)
overall job performance[a]	proficiency at one's job
overall verbal abilities[c]	capacity to understand and use language effectively
pain threshold[c]	minimum intensity of stimulus at which one feels pain
pain tolerance[c]	maximum amount of pain one can endure before stopping stimulus or seeking relief
peer attachment	trust, communication, and non-alienation in peer relationships

perceived responsibility[b]	self-perceptions that others' situation is attributable to the self
perception of own (vs. others') size	subjective estimate of one's size relative to others' size
performance motivation: expectancy	motivation to perform a task based on expectations that performance will result in desired outcomes
performance motivation: goal-setting	motivation to perform a task based on the level and/or difficulty of its goals
performance motivation: self-efficacy	motivation to perform a task based on one's perceived ability to complete the task
performance rating leniency[a]	self-reported measures of leniency in performance ratings
person orientation	extent of attention and response to people (i.e., interest in people)
personal initiative	proactive work behaviors oriented toward overcoming work difficulties and pushing to achievement of desirable work goals
personal space	preferences for personal space
personnel data: commendable behavior[a]	commendations, or absence of disciplinary actions, demotions, and involuntary discharge
personnel data: overall[a]	level changes in job positions, salary, turnover/tenure, and productivity
personnel data: productivity[a]	productivity (e.g., units sold)
personnel data: promotions[a]	number of promotions received
personnel data: salary[a]	salary level or compensation
personnel data: status change[a]	level changes in job positions
perspective-taking	ability to consider and understand others' thoughts, feelings, and beliefs; opposite of self-focus
physical appearance self-esteem	subjective evaluation of worth based on body image and attractiveness
playful emotions	having fun vs. being serious, playing social games with physical contact, humor, and laughter, and being generally happy and joyful
positive affect	pleasant emotions and moods
preference for choice[b]	subjective value derived from choosing one option over others; preference for larger choice sets
preference for feasibility (vs desirability)	a kind of pragmatic thinking, preferring things that can be done over the best thing, if it is more difficult to accomplish
prejudice	negative attitudes toward social groups or categories
proactive personality	tendency for individuals to control situational forces and actively incite change in their environments
procrastination	postponing, delaying, or putting off tasks or decisions
prosocial behavior	behavior that positively weighs others' outcomes in one's decisions

punishment sensitivity	strength of effect of punishment
reaction time	time to respond to a stimulus
regulatory focus: prevention[a]	sensitivity to and self-regulation around negative stimuli or goals at work
regulatory focus: promotion	sensitivity to and self-regulation around posiitve stimuli and goals at work
resilience	capacity, processes, and/or outcomes of successful adaptation in the context of significant threats to functioning or development
resistance to temptation	ability to inhibit a prohibited response
response conflict (executive function)	executive function involving suppressing prepotent or ongoing behaviors that are no longer appropriate or relevant; important for cognitive flexibility in stopping what one is doing when it is not aiding goal progress
reward sensitivity	strength of responsiveness and attraction to rewards
risk propensity	general tendency to differentially focus on the potential positive outcomes, over the potential negative outcomes, of engaging in behavior with an uncertain consequence
risk-taking	behavior or decision-making that may result in negative outcomes
risky impulsivity	risk-taking without prior thought
friendship network centrality	number of ties received from others in socio-emotional social networks
role identification[b]	process of seeing oneself in a role and aspects of the role in the self
roles in instrumental social networks: indegree[a]	number of ties received from others in instrumental social networks
rumination	repetitively and passively focusing on symptoms of distress and on the possible causes and consequences of those symptoms
sadness	feeling helpless to address loss
safety performance[a]	using safety equipment, showing regard for safety procedures, and following workplace safety programs
scholastic achievement[c]	teacher-assigned school scores/grades
seeking emotions	affect that reflects feeling curious, feeling like exploring, striving for solutions to problems and puzzles, positively anticipating new experiences, and a sense of being able to accomplish almost anything
self-compassion	tendency to be caring rather than judgmental of oneself; to understand that all humans, including the self, are imperfect; to not exaggerate or overgeneralize negative aspects of the self beyond the specific situation in which they arise
self-confidence in physical activity	confidence in own ability at physical activities
self-disclosure	revealing and discussing personal information
self-enhancement value: achievement	value personal success and demonstrating competence

self-enhancement value: hedonism[a]	value pleasure and self-gratification
self-enhancement value: power	value social status, dominance, and control over people and resources
self-esteem	subjective evaluation of one's worth as a person
selfishness	concern with one's own personal profit or pleasure; self-serving or egocentric preferences and interpretations, including of fairness
self-leadership	process through which employees influence themselves to achieve self-direction and self-motivation for work-relevant cognition, attitudes, and behaviors
self-rated intelligence	subjective evaluation of one's intelligence; can be general or domain-specific
self-transcendence value: benevolence	value preservation and enhancement of the welfare of in-group members
self-transcendence value: universalism[a]	value tolerance, concern, and appreciation for the welfare of humankind and nature
sensation-seeking	desire for novel or intense experiences, even if risks are involved
sense of personal control	belief that one has influence over outcomes and events in their lives
shame	feeling of self being negatively evaluated; self-conscious emotion
situational judgment tests: behavioral tendency[a]	applicants' likely behavioral responses to given work-related situations
situational judgment tests: knowledge[a]	applicants' evaluations of the effectiveness of possible responses to work-related situations
smiling	universal expression of positive emotion, authentic or socially prescribed
social dominance orientation	degree of preference for inequality among social groups
social vocational interests	career interests in activities involving working with and helping others
source attribution accuracy[b]	accuracy at recalling who said what
spatial visualization	one component of visuospatial ability; mentally manipulating and transforming objects and shapes
spatial working memory	capacity to temporarily store and manipulate spatial information, such as relationships between objects
stereotyping	beliefs about attributes of a social group ascribed to individual members of the group
stress	psychological response when the demands of the situation are perceived to exceed one's ability to cope
study attitudes[a]	positive attitude toward studying
study habits	regular engagement in sound study behaviors and routines
subjective well-being	high life satisfaction combined with high positive affect and low negative affect
susceptibility to influence	susceptibility to be influenced by others
systemizing quotient[c]	drive to analyze and construct systems by understanding the governing rules

talkativeness	quantity of verbal communication
task-based organizational citizenship behavior	individuals' efforts to improve their effectiveness in performing their work tasks, including displaying greater conscientiousness, persevering on tasks until they are completed, and acquiring additional work skills
task-oriented leadership	organizing activities to perform tasks
technical performance[a]	proficiency in performing activities formally recognized as part of the job, which contribute to the organization's technical core
temporal discounting	devaluing future rewards compared to immediate rewards; includes delay of gratification and delay discounting
tentative speech	hesitation and uncertainty in language
thing orientation[c]	extent of attention and response to objects (i.e., interest in things)
time judgment[c]	judgments of duration
touch initiation[c]	being the first to touch in a dyadic interaction
transactional leadership: contingent reward[a]	behaviors intended to exchange tangible or nontangible support and resources with followers based on their efforts and performance
transactional leadership: management by exception[a]	behaviors involving setting performance standards and monitoring deviations, acting on an as-needed basis
transactional leadership: passive leadership[a]	behaviors involving passive leadership and intervening only when problems become serious
transformational leadership: charisma[a]	followers identify with a leader's vision and leader fosters optimism via action
transformational leadership: individualized consideration	behaviors providing followers with opportunities for growth and development, coaching, and personalized consulting
transformational leadership: intellectual stimulation[a]	behaviors exhorting followers to reframe problems, develop novel ideas, or approach issues in new ways
trust in others	belief that another person will act with benevolence, reliability, and integrity; the opposite of cynicism
trustworthiness[b]	behavior reflects positive intentions and goodwill toward others
turnover[a]	voluntary quitting and discharge
turnover intentions[a]	intention to turnover from an employer
updating (executive function)	executive function involving monitoring for adverse or unexpected outcomes within tasks, including conflicts between options, commission of errors and updating working memory; important for cognitive flexibility in processing feedback and recognizing that what one is doing is not facilitating goal progress
verbal fluency[c]	ability to generate as many words as possible that fulfill a certain criterion, normally under time restrictions

verbal working memory[c]	capacity to temporarily store and manipulated verbal information, such as letters, syllables, words, nonwords, digits, or nameable objects
verbal-episodic memory[c]	verbal content in memory of events in one's experience encoded in a particular temporal-spatial context
visual-spatial working memory	processes involved in the storage of spatial or visual information over a limited period of time
vividness of visual imagery[c]	level of detail, clarity, and sensory richness of mental images that a person can generate in their mind
voice	discretionary expressions of opinions, beliefs, or attitudes
voluntary turnover[a]	voluntary quitting
withdrawal behavior[a]	avoidance of or disengagement from work environment, tasks, or the organization
working memory[c]	cognitive system for temporarily holding and manipulating information necessary for completing tasks
work-life balance: family interference with work[a]	degree to which family role participation interfered with work role responsibilities
work-life balance: work interference with family[a]	degree to which work role participation interferes with family role responsibilities
work-life balance: work-nonwork negative spillover[a]	degree to which work (nonwork) role participation is worsened by participating in the other role
work-life balance: work-nonwork positive spillover[a]	degree to which work (nonwork) role participation is improved by participating in the other role
workplace harassment perceptions[a]	perception that coworkers engage in bullying, victimization, and other hostile behaviors
**Level III Construct**<br>**Level II Construct**	
Agency	
Dominance	extent to which one takes control and attempts to influence others, including simply assertive and direct expression of one's own needs, opinions, and desires
Goal Approach & Disinhibition	extent to which one seeks out and attempts to make progress toward goals, including selecting and perceiving others and situations instrumentally; is proactive and oriented toward taking action; is responsive to rewards; and/or lacks restraint or regard of rules or norms for behavior
Risk-Seeking	extent to which one behaves or makes decisions such that there is a significant possibility of negative outcomes
Communion	
Interpersonal Sensitivity	extent to which one has high motivation, attention, and/or accuracy related to understanding others' thoughts, needs, and feelings

Sociability	extent to which one engages in social interactions, forms and maintains social connections, and participates in social activities
Prejudice & Dehumanization	extent to which one has strong negative attitudes toward social groups/categories, stereotypes outgroup members, and/or neglects others' attributes that define their individuality or humanness
Self-Evaluation	
Positive Views of the Self	extent to which one has positive perceptions and/or evaluations globally of the self or of specific attributes of the self
Well-Being	extent to which one experiences high life satisfaction, high ratio of positive to negative affect, positive relations, high autonomy, high environmental mastery, high personal growth, high purpose in life, and/or high self-acceptance
Cognitive Processes	
Spatial Ability/Performance	extent to which one demonstrates ability to perceive, reason about, and mentally manipulate visual information
Creative Performance	extent to which one generates novel and different ideas and/or develops novel and useful products
Abstract Cognition	extent to which one thinks about central and superordinate features of events, situations, and targets, and demonstrates ability to interpret and reason about patterns

[a] Extraversion effect construct could not be categorized
[b] Power effect construct could not be categorized
[c] Sex/gender difference construct could not be categorized

Table SI11. Key PRISMA guidelines: Actions taken, or reason step not applicable.

**PRISMA Guideline**	**Action Taken/Reason Excluded**
(5) Inclusion/exclusion criteria for studies and how studies were grouped for syntheses	Our diagram has inclusion/exclusion rules.<br>For grouping of studies, our goal was to match power and sex/gender difference constructs as much as possible. Therefore, we first grouped power experiments into Level 1 constructs based on iterations between i) measures, ii) constructs/concepts that the measures operationalized, and iii) outcomes that had been meta-analyzed in the sex/gender difference literature
(6) Specify all databases searched for studies. Specify the date when each database was last searched.	See Table SI1
(7) Present the full search strategies, including filters, for all source databases.	See Table SI1
(8) Specify methods used to decide on a study's inclusion/exclusion, including how many screeners per record, whether they worked independently, and any automation tools used	For inclusion/exclusion of power studies, we involved four total screeners, two of whom reviewed each record but not independently. The last author made all final decisions in consultation with the rest of the author team.<br>For inclusion/exclusion of sex/gender meta-analyses, one screener was involved because we were selecting only the most recent meta-analysis for each construct that involved non-clinical adult samples, i.e., the criteria were objective and observable
(9) Specify methods used to collect data from papers, including how many reviewers, whether they worked independently, any processes for obtaining or confirming data from study investigators, and any automation tools used	To collect power experiments, we involved four total screeners, two of whom reviewed each record but not independently.<br>To collect sex/gender and extraversion meta-analyses, one screener (the last author) selected all outcome variables in the most aggregated form unless there was a substantively different sex/gender or extraversion effect when the construct was disaggregated (e.g., indirect vs. direct aggression)
(10a) List and define all outcomes for which data were sought.	From the power experiments, we collected data for all Level I and Level II constructs (see also Table SI1)
(10b) List and define all other variables for which data were sought (e.g., participant characteristics).	Excluded. This is for moderator analyses, which we did not conduct.

(11) Specify the methods used to assess risk of bias in the included studies	Excluded because our inclusion/exclusions criteria of power studies specified that we only select significant effects (*P*-curve analysis versus meta-analysis)
(12) For each outcome, specify effect measure used in the synthesis or presentation of results	Cohen's d, except where noted.
(13a) Describe processes used to decide which studies were eligible for each synthesis.	If a study treated power as the independent variable, or if a meta-analysis included a sex/gender difference effect size, and it satisfied our inclusion/exclusion criteria, it was included in the comparison of sex/gender differences to power effects. If a meta-analysis included an effect of extraversion, it was included in the comparison of extraversion effects to sex/gender differences.
(13b) Describe methods to prepare the data for presentation or synthesis.	Generally assumed equal *n* per condition or used inferential statistic and *df*; if an effect size could not be obtained, study was removed.
(13c) Describe methods to tabulate or visually display results of individual studies and syntheses.	For power experiments, we visually displayed the data using the *P*-curve website.
(13d) Describe methods to synthesize results and rationale for choice	See description in paper of the creation of Level I constructs nested within Level II constructs nested within Level III categories.
(13e) Describe methods used to explore heterogeneity among results.	Excluded. This is not a meta-analysis.
(13f) Describe sensitivity analyses.	Excluded. This is not a meta-analysis.
(14) Describe methods used to assess risk of bias due to missing results.	Excluded. This is not a meta-analysis.
(15) Describe methods used to assess confidence in the body of evidence for an outcome	Excluded. This is not a meta-analysis.
